# White matter microstructure is differentially impacted by cerebral amyloid angiopathy, neurofibrillary tangles, and neuritic plaque co‐pathology

**DOI:** 10.1002/alz.70637

**Published:** 2025-10-14

**Authors:** Alexandra Santos, Francisco C. Almeida, Kathryn Gauthreaux, Charles N. Mock, Walter A. Kukull, John F. Crary, Tiago Gil Oliveira

**Affiliations:** ^1^ Life and Health Sciences Research Institute (ICVS), School of Medicine University of Minho Gualtar Portugal; ^2^ ICVS/3B's—PT Government Associate Laboratory Gualtar Portugal; ^3^ Department of Neuroradiology Centro Hospitalar Universitário do Porto Porto Portugal; ^4^ Department of Epidemiology, National Alzheimer's Coordinating Center University of Washington Seattle Washington USA; ^5^ Neuropathology Brain Bank & Research Core, Department of Pathology, Nash Family Department of Neuroscience, Department of Artificial Intelligence & Human Health, Friedman Brain Institute, Ronald M. Loeb Center for Alzheimer's Disease Icahn School of Medicine at Mount Sinai New York New York USA; ^6^ Department of Neuroradiology Hospital de Braga ULS Braga Braga Portugal

**Keywords:** Alzheimer's disease, cerebral amyloid angiopathy, co‐pathology, diffusion tensor imaging, magnetic resonance imaging, neuritic plaques, neurofibrillary tangles

## Abstract

**INTRODUCTION:**

White matter (WM) is affected by and serves as a pathway to neurofibrillary tangle (NFT) propagation in Alzheimer's disease (AD). Cerebral amyloid angiopathy (CAA) associates with neuritic plaques (NPs) to exacerbate NFT accumulation. We aim to study how these co‐pathologies affect WM integrity.

**METHODS:**

We performed a cross‐sectional study of *ante mortem* diffusion tensor imaging (DTI) data according to participants’ *post mortem* NFT, NP, and CAA neuropathology, from the National Alzheimer's Coordinating Center dataset.

**RESULTS:**

We found asymmetric DTI changes in several WM regions between Braak NFT stages II and IV and V/VI, and across CAA pathological burden, with increased mean, radial, and axial diffusivities. CAA‐NFT co‐pathology effects were observed mainly in the splenium of the corpus callosum. DTI metrics were associated with cognitive function and hippocampal volumes.

**DISCUSSION:**

Our results suggest that WM integrity is differentially impacted by AD neuropathology, with CAA and NFTs influencing each other's effects on WM microstructure.

**Highlights:**

- Diffusion tensor imaging (DTI) changes were observed in several white matter (WM) regions between advanced Braak stages and across cerebral amyloid angiopathy (CAA).
- CAA demonstrated a greater WM impact on the right hemisphere, while neurofibrillary tangles (NFTs) had greater impact on the left.
- CAA–NFT concurrent effects were mainly noticed in the splenium of the corpus callosum.
- WM DTI metrics were associated with cognition and hippocampal volumes.

## 1 BACKGROUND

Alzheimer's disease (AD) is primarily characterized by the accumulation of extracellular neuritic plaques (NPs), consisting of amyloid beta (Aβ) surrounded by dystrophic neurites, and neurofibrillary tangles (NFTs), which are mainly composed of abnormally aggregated tau.^1^ In addition to the progressive accumulation of these pathologies in gray matter (GM), AD autopsy cases show white matter (WM) damage, as described by axonal, myelin, and oligodendrocytes loss.^2^, ^3^

Diffusion tensor imaging (DTI)^4^ is the most used diffusion magnetic resonance imaging (MRI) technique to non‐invasively study WM structure. By quantifying the displacement distribution of the Gaussian diffusivity along three main directions, microstructural metrics are derived, namely, fractional anisotropy (FA), radial diffusivity (RD), axial diffusivity (AxD), and mean diffusivity (MD). Such metrics are sensitive to changes in tissue architecture,^5^ and particularly in regions where there is a predominant fiber direction, they correlate with axonal and myelin content,^6^, ^7^ and therefore serve as a proxy of WM integrity.

Several studies have consistently shown changes in DTI metrics early in the AD continuum. Decreased FA in the fornix was observed in participants with mild cognitive impairment (MCI) and correlated with their cognitive performance and hippocampal volume.^8^, ^9^ In MCI and AD participants, reductions in FA and increases in MD were observed across several WM regions, including the cingulum, corpus callosum, corona radiata, fronto‐occipital, and longitudinal fasciculus.^10^, ^11^, ^12^, ^13^ Some of these changes were reported to coincide with regions that also exhibited increased RD and AxD.^12^, ^13^ These early alterations in DTI appear to follow a non‐linear pattern, as intermediate amyloid positron emission tomography (PET) levels correlated with increased FA and decreased RD in several regions,^14^, ^15^ which moved in the opposite direction with higher Aβ burden.

The progressive deterioration of WM appears to be both an inherent consequence of and a pathway for the spread of AD pathology. Using an epidemic model applied to DTI‐derived connectivity data, regional tau accumulation was predicted in a PET imaging study. These findings demonstrated that tau pathology preferentially propagates through highly connected regions of intrinsic brain networks, seeding from the entorhinal cortex, and is further accelerated by the presence of Aβ.^16^ Consistent with these findings, specific patterns of diffusivity alterations in highly connected WM tracts, including an increase in MD, driven largely by RD, were associated with tau PET accumulation. In contrast, no such connectivity pattern was found with amyloid PET accumulation.^17^ Further emphasizing the concept of tau spreading through connected networks, the diffusivity in the hippocampal cingulum bundle predicted tau accumulation in the directly connected posterior cingulate cortex, but only in Aβ‐positive individuals.^18^ Cortical tau levels were more associated with and better predicted WM damage than Aβ load.^19^ In agreement, higher *post mortem* Braak NFT stages^20^, ^21^ were associated with *ante mortem* higher MD and lower FA in medial temporal and parietal WM regions, contrary to NP severity.^22^ Therefore, while Aβ exacerbates tau‐associated WM damage, tau accumulation seems to have a more direct and stronger correlation with WM integrity.

Additional neuropathological changes frequently coexist and interact with NPs and NFTs in AD, further accelerating the progression of dementia. For instance, cerebral amyloid angiopathy (CAA), defined by the deposition of Aβ in the walls of small and medium‐sized cerebral blood vessels, is strongly associated with AD NPs.^23^ Emerging evidence demonstrates that in the presence of abundant NPs, CAA exacerbates NFT accumulation, thereby accelerating cognitive decline.^24^, ^25^ Participants with CAA have a higher risk of developing amyloid‐related imaging abnormalities (ARIAs) associated with anti‐Aβ immunotherapy.^26^ CAA has also been associated with WM alterations, which correlated with worse cognitive performance.^27^, ^29^

This study aims to clarify the distinct impact of NFTs and NPs on WM integrity, particularly in advanced AD stages often complicated by co‐pathologies, namely CAA. We investigated how the *ante mortem* DTI metrics were affected by the severity of *post mortem* NFTs, NPs, and CAA, and how they correlated with clinical dementia scores and hippocampal volumes.

**RESEARCH IN CONTEXT**

1. **Systematic review**: Cerebral amyloid angiopathy (CAA) co‐pathology is highly prevalent in Alzheimer's disease (AD), exacerbating neurofibrillary tangle (NFT) accumulation and contributing to cognitive dysfunction. While several studies demonstrate an impact of CAA in white matter (WM) integrity, whether it has an effect in WM integrity in AD has not been studied.
2. **Interpretation**: Our study shows that CAA severity alters WM microstructure in AD. These effects were influenced by the NFT burden and vice versa, suggesting that the presence of CAA co‐pathology must be considered a significant potentiator of disease progression.
3. **Future directions**: It would be relevant to explore the NFT and CAA WM effects longitudinally in a larger AD dataset, to more specifically address how the severity of co‐pathology correlates with disease evolution, and to further investigate potential disease heterogeneity.

## 2 METHODS

### 2.1 Participants

Data were obtained from the National Alzheimer's Coordinating Center (NACC),^29^, ^30^ a repository for data collected at Alzheimer's Disease Research Centers (ADRCs) across the United States. The ADRCs gather neuropathological assessments from participants who consented to autopsy, as well as standardized clinical data via the Uniform Data Set (UDS).^31^ Using the March 2024 NACC data freeze, participants were categorized according to the Braak stage (none, II–IV, V/VI) for NFT degeneration, Consortium to Establish a Registry for Alzheimer's Disease (CERAD) scores for the density of NPs (0: none, 1: sparse, 2: moderate, 3: frequent), and CAA severity (0: none, 1: mild, 2: moderate, 3: severe). Details on brain tissue preparation and staining within the NACC neuropathology dataset have been previously described.^30^

Inclusion was restricted to participants who had: undergone both T1‐weighted (T1w) and DTI MRI; subsequent neuropathological assessment at autopsy, with Braak, CERAD, and CAA data available; a maximum interval of 4 years between MRI and autopsy; UDS data on the Clinical Dementia Rating (CDR) Global scale (0: no impairment, 0.5: questionable impairment, 1: mild impairment, 2: moderate impairment, 3: severe impairment). The MRI acquisition and clinical UDS closest to death were selected. From this cohort, our study sample of 26 participants was obtained after excluding participants with a CERAD score and a Braak stage of none.

### 2.2 MRI data acquisition and pre‐processing

The MRI data provided by the NACC were acquired at six ADRCs on 1.5T (*N* = 1) and 3T (*N* = 25) scanners, from Siemens (*N* = 3) or GE (*N* = 23) manufacturers (Table S1 in supporting information). For our imaging analyses, we used T1w and DTI acquisitions. DTI data were acquired using different parameters, depending on the site‐specific protocol. Specifically, images were collected with either *b* = 1000 or 1300 s/mm^2^, using 25 to 80 diffusion‐weighted directions and 1 to 12 non‐diffusion‐weighted (*b* = 0) images (Table S1). More MRI scanner specifications and details about structural and diffusion acquisition protocols are available in Table S1 and at www.alz.washington.edu.

Hippocampal volumes were obtained from the volumetric T1w images using FreeSurfer software (version 7.0, https://surfer.nmr.mgh.harvard.edu/). Subcortical segmentation was done using the Aseg atlas.^32^ Left and right hippocampal volumes were normalized to the estimated total intracranial volume of each participant, to account for differences in head size.

The DTI preprocessing pipeline first included principal component analysis denoising^33^ and Gibbs ringing correction using the Diffusion in Python (DIPY) package.^34^ Brain masks were computed using the FMRIB software library (FSL, version 6.0.1, http://www.fmrib.ox.ac.uk/fsl/) bet tool. The echo‐planar imaging distortions were then corrected using Advanced Normalization Tools (ANTs)^35^ non‐linear registration of the DTI *b* = 0 volume to the T1w image, constrained only in the phase encoding direction. The estimated correction was then warped back to each DTI volume in native space. The distortion‐corrected images were further corrected for eddy currents and motion^36^ using FSL's eddy. The diffusion tensor was then estimated in each voxel using DIPY, and maps of FA, MD, RD, and AxD were derived for each participant.

### 2.3 DTI region of interest analysis

The FA map of each participant was registered to the Johns Hopkins University (JHU)—International Consortium of Brain Mapping (ICBM) FA template^37^ using ANTs non‐linear registration. Subsequently, the WM regions of interest (ROIs) from the JHU‐ICBM‐DTI‐48 WM atlas (https://identifiers.org/neurovault.image:1401) were warped into the participant's space using reverse transformation. For each participant, FA, MD, RD, and AxD values were extracted for all the JHU‐defined WM ROIs in the native space. To avoid the inclusion of non‐WM tissue, a threshold was applied to all the maps, excluding regions with FA values < 0.2.

ROI segmentation quality was assessed using two criteria: visual inspection, in which the transformed ROIs were overlaid on each subject's FA map to assess anatomical plausibility and spatial correspondence; and value checking, in which ROIs yielding missing values were identified, likely reflecting imperfect mapping into native space due to registration limitations or absence after FA thresholding. ROIs that failed at least one of the two criteria were considered poorly segmented and excluded from further analysis. As a result, we analyzed data from the following 35 WM regions: genu, body, and splenium of the corpus callosum; left and right fornix; left and right anterior, posterior, and retrolenticular limbs of the internal capsule; left and right external capsule; left and right anterior, superior, and posterior portions of the corona radiata; left and right cingulum (cingulate gyrus and hippocampal portions separately); left and right superior longitudinal fasciculus; left and right corticospinal tract; left and right posterior thalamic radiation; left and right sagittal stratum; left and right superior fronto‐occipital fasciculus; and left and right tapetum.

For visualization purposes, only the JHU WM atlas ROIs showing statistically significant group differences were displayed, overlaid on the MNI152 T1 template. Because the WM JHU atlas is defined in Montreal Neurological Institute (MNI) space, and was used throughout the analysis, no additional transformations were required for plotting. This allowed for clear anatomical localization of the significant regions using a standard reference template. The brain figures were created using the Nilearn Python package.

### 2.4 Statistical analysis

All statistical analyses were performed using RStudio (R version 4.4.1). Data normality was evaluated using the Shapiro–Wilk test. To evaluate potential differences between different Braak stages in terms of demographic and other neuropathologic characteristics, Kruskal–Wallis test and Fisher tests were performed, as appropriate. To correct for the effect of covariates, linear regression models were fitted with regional DTI metrics or hippocampal volumes as the dependent variables, and age, sex, b‐value, and either the MRI‐to‐death or MRI‐to‐clinical visit interval as independent variables, depending on the specific analysis. Residuals from these regression models were extracted and used for both group comparisons and correlation analyses. Braak stage, CAA severity, or the presence of Lewy body (LB) pathology were included as additional covariates in the linear models, according to the analysis, to test the effects independently of the co‐pathology. The Wilcoxon rank‐sum test was applied to assess differences in DTI metrics across WM regions between the Braak stages II to IV and V/VI groups, as well as between CERAD scores 2 and 3. Comparisons involving the CERAD score 1, respectively within its corresponding CERAD analyses, were not conducted due to very small sample sizes. Results were adjusted for multiple comparisons using the false discovery rate (FDR) method with the Benjamini–Hochberg (BH) approach. For comparisons of DTI metrics across WM regions among different CAA groups, the Kruskal–Wallis test was used to evaluate overall group differences. When significant differences were detected (*P* < 0.05), a Dunn post hoc test with BH FDR correction was used for pairwise comparisons. Correlation analyses between the regional DTI metrics and CDR scores, as well as with hippocampal volumes, were performed using the Spearman rank correlation test. FDR adjustments with the BH method were applied to control for multiple comparisons.

## 3 RESULTS

### 3.1 Demographics and neuropathological characteristics

The demographics and neuropathological data categorized according to the Braak stages are summarized in Table 1. Of the 26 participants, 17 had Braak stage V/VI and 9 had Braak stage II to IV. There were no statistically significant differences among Braak groups in terms of age (*P* = 0.36, Wilcoxon rank sum test), sex (*P* = 0.14, Fisher test), or years of education (*P* = 0.91, Wilcoxon rank sum test). There were statistically significant differences in CERAD scores among Braak stages (*P* = 0.036, Fisher test). While the Braak II to IV group consisted mainly of participants with moderate NPs (67%), participants with Braak V/VI were almost equally distributed between moderate (47%) and frequent (53%) CERAD scores, demonstrating that the NFT burden progression is accompanied by an increase in NP accumulation. The proportions of CAA severity were not significantly different among Braak stages (*P* = 0.72, Fisher test). However, there was a trend for the proportion of Braak V/VI to increase with increasing CAA severity (Figure S1 in supporting information).

**TABLE 1:** Demographic and neuropathological characteristics of participants grouped by Braak stage.

	Braak II–IV *N* = 9^a^	Braak V/VI *N* = 17^a^	*P* value^b^
**Age at MRI**	81.67 ± 6.16	77.12 ± 8.58	0.36
**Females**	4/9 (44%)	2/17 (12%)	0.14
**Years of education**	15.67 ± 2.92	15.47 ± 2.92	0.91
**CERAD**			0.036
Sparse	2/9 (22%)	0/17 (0%)	
Moderate	6/9 (67%)	8/17 (47%)	
Frequent	1/9 (11%)	9/17 (53%)	
**CAA**			0.72
None	3/9 (33%)	3/17 (18%)	
Mild	4/9 (44%)	6/17 (35%)	
Moderate	1/9 (11%)	3/17 (18%)	
Severe	1/9 (11%)	5/17 (29%)	

Abbreviations: CAA, cerebral amyloid angiopathy; CERAD, Consortium to Establish a Registry for Alzheimer's Disease; MRI, magnetic resonance imaging.

^a^ Mean ± standard deviation; n/N (%).

^b^ Wilcoxon rank sum test; Fisher exact test.

### 3.2 WM integrity is differentially affected by NFTs and NPs

**FIGURE 1:**
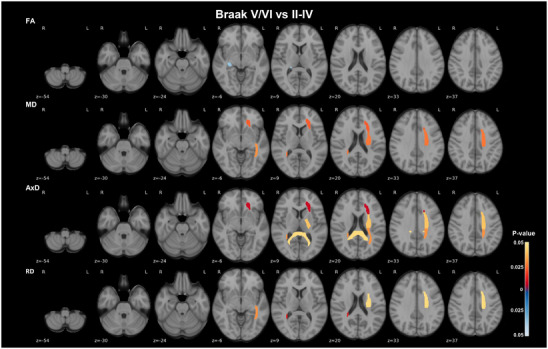
Asymmetric regional changes in WM integrity across advanced Braak stages in AD. Axial views of WM ROIs with differences in DTI metrics linear model residuals for age, sex, b‐value, and MRI‐to‐death interval, between Braak stages II to IV and V/VI, overlaid on the MNI 152 T1 template are shown. The JHU‐ICBM‐DTI‐48 WM atlas was used to extract regional DTI metrics for each participant. Comparisons revealed statistically significant group differences largely affecting the left hemisphere: corona radiata, sagittal stratum, posterior limb of internal capsule, but also the right hemisphere fornix and tapetum, and the splenium of the corpus callosum. Only statistically significant comparisons (*P* < 0.05 according to the Wilcoxon test) are shown. The color bars show the *P* values and the direction of the statistical differences, with red denoting higher values in Braak V/VI relative to Braak II‐IV, and blue denoting the reverse. AD, Alzheimer's disease; AxD, axial diffusivity; DTI, diffusion tensor imaging; FA, fractional anisotropy; ICBM, International Consortium of Brain Mapping; JHU, Johns Hopkins University; MD, mean diffusivity; MNI, Montreal Neurological Institute; MRI, magnetic resonance imaging; RD, radial diffusivity; ROI, regions of interest; WM, white matter

**FIGURE 2:**
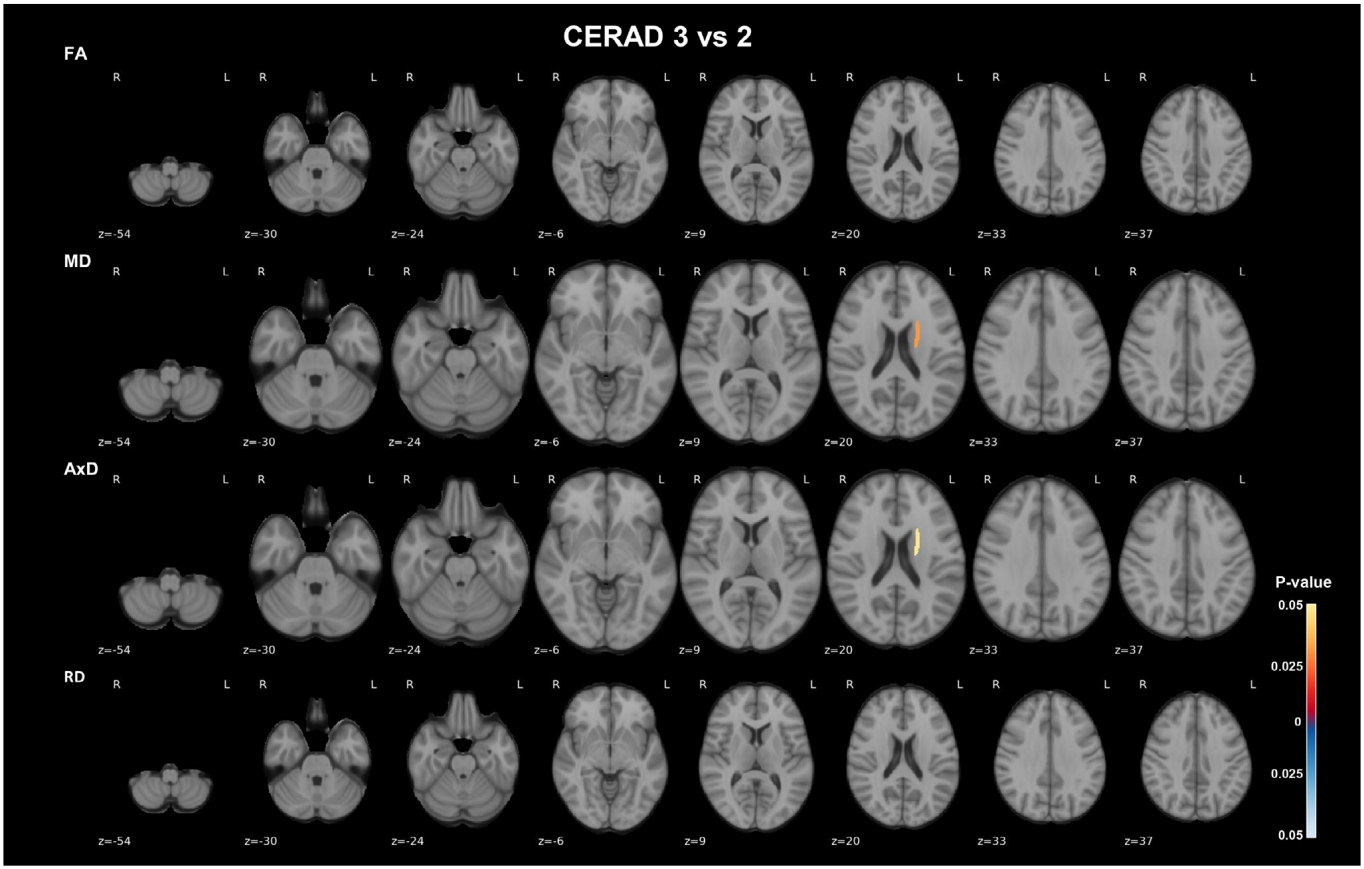
Left frontal‐occipital fasciculus diffusivity increases with higher CERAD scores. Axial views of WM ROIs with differences in DTI metrics linear model residuals for age, sex, b‐value, and MRI‐to‐death interval, between CERAD scores 2 and 3, overlaid on the MNI 152 T1 template are depicted. The JHU‐ICBM‐DTI‐48 WM atlas was used to extract regional DTI metrics for each participant. Comparisons revealed statistically significantly higher AxD and MD in the left hemisphere superior frontal‐occipital fasciculus from CERAD score 3 group, compared to CERAD score 2. Only statistically significant comparisons (*P* < 0.05 according to the Wilcoxon test) are shown. The color bars show the *P* values and the direction of the statistical differences, with red denoting higher values in CERAD 3 relative to CERAD 2, and blue denoting the reverse. AxD, axial diffusivity; CERAD, Consortium to Establish a Registry for Alzheimer's Disease; DTI, diffusion tensor imaging; FA, fractional anisotropy; ICBM, International Consortium of Brain Mapping; JHU, Johns Hopkins University; MD, mean diffusivity; MNI, Montreal Neurological Institute; MRI, magnetic resonance imaging; RD, radial diffusivity; ROI, region of interest; WM, white matter

Statistical analyses of regional DTI metrics across groups, categorized based on each neuropathological condition, are summarized in Table S2 in supporting information. ROI analysis detected widespread statistically significant changes in DTI metrics between Braak stages II to IV and V/VI, affecting mostly WM regions of the left hemisphere. From Braak II to IV to V/VI, there was a marked increase in diffusivity in the left anterior corona radiata, as shown by statistically significant higher MD, and predominantly AxD (*P* = 0.006, Wilcoxon test), and in the left superior and posterior corona radiata, sagittal stratum, and posterior limb of internal capsule detected as statistically significant increase in either one or more of MD, AxD, and RD (*P* < 0.04, Wilcoxon test). In the right hemisphere, a statistically significant lower FA in the fornix (*P* = 0.034, Wilcoxon test), and higher MD, AxD, and largely RD (*P* = 0.011, Wilcoxon test) in the anatomically adjacent tapetum, were shown in Braak stage V/VI. A statistically significant increase in AxD, though to a lesser extent, was also observed in the splenium of the corpus callosum (*P* = 0.045, Wilcoxon test; Figure 1; Table S2 and Figure S2 in supporting information). Participants with CERAD 3 showed a statistically significant increase in AxD and predominantly MD in the superior fronto‐occipital fasciculus compared to those with CERAD 2 (*P* < 0.05, Wilcoxon test; Figure 2; Table S2 and Figure S3 in supporting information). No statistical comparisons were performed with CERAD 1, due to the very small sample size. However, a stepwise increase in diffusivity appears to occur along NP burden progression (Figure S3). None of the statistically significant differences between Braak stages II to IV and V/VI, and between CERAD 2 and 3 were detectable after correction for FDR (Table S2), likely due to the limited statistical power associated with the small sample size.

### 3.3 CAA severity impacts WM integrity in AD

**FIGURE 3:**
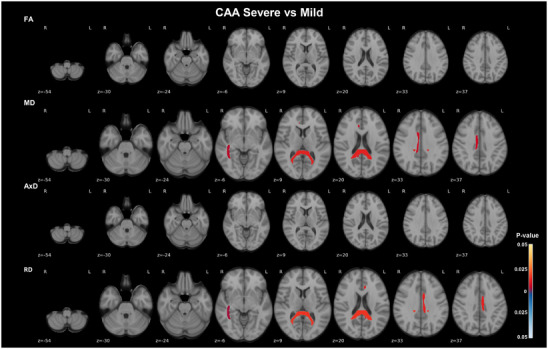
CAA severity is associated with asymmetric alterations in WM integrity in AD. Axial views of WM ROIs with differences in DTI metrics linear model residuals for age, sex, b‐value, and MRI‐to‐death interval, between severe and mild CAA, overlaid on the MNI 152 T1 template are shown. The JHU‐ICBM‐DTI‐48 WM atlas was used to extract regional DTI metrics for each participant. Comparisons revealed statistically significant group differences in the right sagittal stratum, cingulum, and in the splenium of the corpus callosum. Only statistically significant comparisons (*P* < 0.05) identified using the Kruskal–Wallis test followed by Dunn post hoc test are presented. The color bars show the *P* values and the direction of the statistical differences, with red denoting higher values in severe CAA relative to mild CAA, and blue denoting the reverse. AxD, axial diffusivity; CAA, cerebral amyloid angiopathy; DTI, diffusion tensor imaging; FA, fractional anisotropy; ICBM, International Consortium of Brain Mapping; JHU, Johns Hopkins University; MD, mean diffusivity; MNI, Montreal Neurological Institute; MRI, magnetic resonance imaging; RD, radial diffusivity; ROI, regions of interest; WM, white matter

Statistically significant differences were observed in the cingulum, right sagittal stratum, and in the splenium of the corpus callosum across groups defined by CAA severity. These differences were primarily reflected in increased MD and RD in severe CAA, predominantly compared to mild CAA (*P* < 0.016, Kruskal–Wallis followed by Dunn post hoc test; Figure 3; Table S2 and Figure S4 in supporting information). These statistically significant differences remained after applying FDR corrections, except for those observed in the splenium of the corpus callosum and left cingulum (Table S2).

To determine whether the observed differences between CAA groups were influenced by NFT burden, we accounted for the effect of Braak stage by including it in the linear model alongside the previously used covariates. From mild to severe CAA, no regions remained statistically significant after correcting for Braak pathology. However, we found statistically significant alterations in the genu of the corpus callosum AxD, mainly between none to severe CAA (*P* = 0.033, Kruskal–Wallis followed by Dunn post hoc test, FDR adjusted; Table S3 in supporting information). Interestingly, Wilcoxon tests showed that the left anterior corona radiata was the only region where AxD remained statistically significant, despite not surviving FDR corrections, between Braak II to IV and V/VI after correcting for the linear effect of CAA (*P* = 0.025, Wilcoxon test; Table S3 and Figure S5 in supporting information). These results suggest that the splenium of the corpus callosum appeared as a highly vulnerable region to the combined effects of NFTs and CAA co‐pathology, as significant changes were observed in both CAA and NFT analyses, but disappeared after correction for the co‐pathology. Furthermore, the effects of CAA and NFT burden on DTI metrics across multiple WM ROIs were strongly inter‐connected, with CAA having a greater impact on the right hemisphere and NFTs on the left hemisphere (Figures 1 and 3; Figure S5).

### 3.4 WM DTI metrics correlate with cognition and hippocampal volumes

**FIGURE 4:**
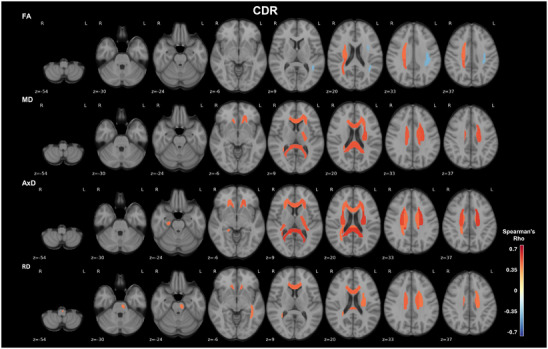
Regional DTI metrics are positively associated with the CDR scores in AD participants. Axial views of WM ROIs with correlations between DTI metrics linear model residuals for age, sex, b‐value, and MRI‐to‐clinical visit interval, and CDR global scores, overlaid on the MNI 152 T1 template are shown. The JHU‐ICBM‐DTI‐48 WM atlas was used to extract regional DTI metrics for each participant. Only statistically significant correlations (*P* < 0.05) identified using Spearman correlation test are presented. The color bars show the Spearman correlation coefficients (rho), with red and blue scales indicating positive and negative correlations, respectively. AD, Alzheimer's disease; AxD, axial diffusivity; CDR, Clinical Dementia Rating; DTI, diffusion tensor Imaging; FA, fractional anisotropy; ICBM, International Consortium of Brain Mapping; JHU, Johns Hopkins University; MD, mean diffusivity; MNI, Montreal Neurological Institute; MRI, magnetic resonance imaging; RD, radial diffusivity; ROI, regions of interest; WM, white matter

To evaluate whether the observed alterations in WM integrity were associated with the severity of dementia, we analyzed the correlations between CDR scores and age‐, sex‐, b‐value‐, and MRI‐to‐visit interval‐corrected DTI metrics. We found statistically significant positive correlations between diffusion metrics and CDR across multiple ROIs, with the strongest associations observed predominantly with AxD in the splenium of the corpus callosum and left superior corona radiata (rho > 0.5, *P* = 0.005, Spearman correlation), but also with their MD (rho > 0.47, *P* < 0.016, Spearman correlation; Figure 4; Table S4 in supporting information). Interestingly, these ROIs were among the ones previously identified as exhibiting neuropathology‐related diffusivity changes in Braak stages V/VI. Significant CDR correlations were also observed with diffusivities in the body and genu of the corpus callosum; anterior, right posterior, and superior corona radiata; left sagittal stratum; posterior limb and retrolenticular part of internal capsule; corticospinal tract; right tapetum; and hippocampus cingulum (Figure 4; Table S4). Interestingly, FA in the right posterior and superior corona radiata was statistically significantly positively correlated with CDR (rho > 0.40, *P* < 0.05, Spearman correlation). This correlation goes in the opposite direction to the ones observed in other tracts, such as the statistically significant negative correlation between CDR and FA in the left superior longitudinal fasciculus (rho = –0.401, *P* = 0.042, Spearman correlation; Table S4). This might be explained by a higher prevalence of crossing fibers in the corona radiata, where possibly a fiber loss occurring along CDR progression may paradoxically lead to FA increases. The significance of the correlations was lost in all the ROIs after FDR corrections.

**FIGURE 5:**
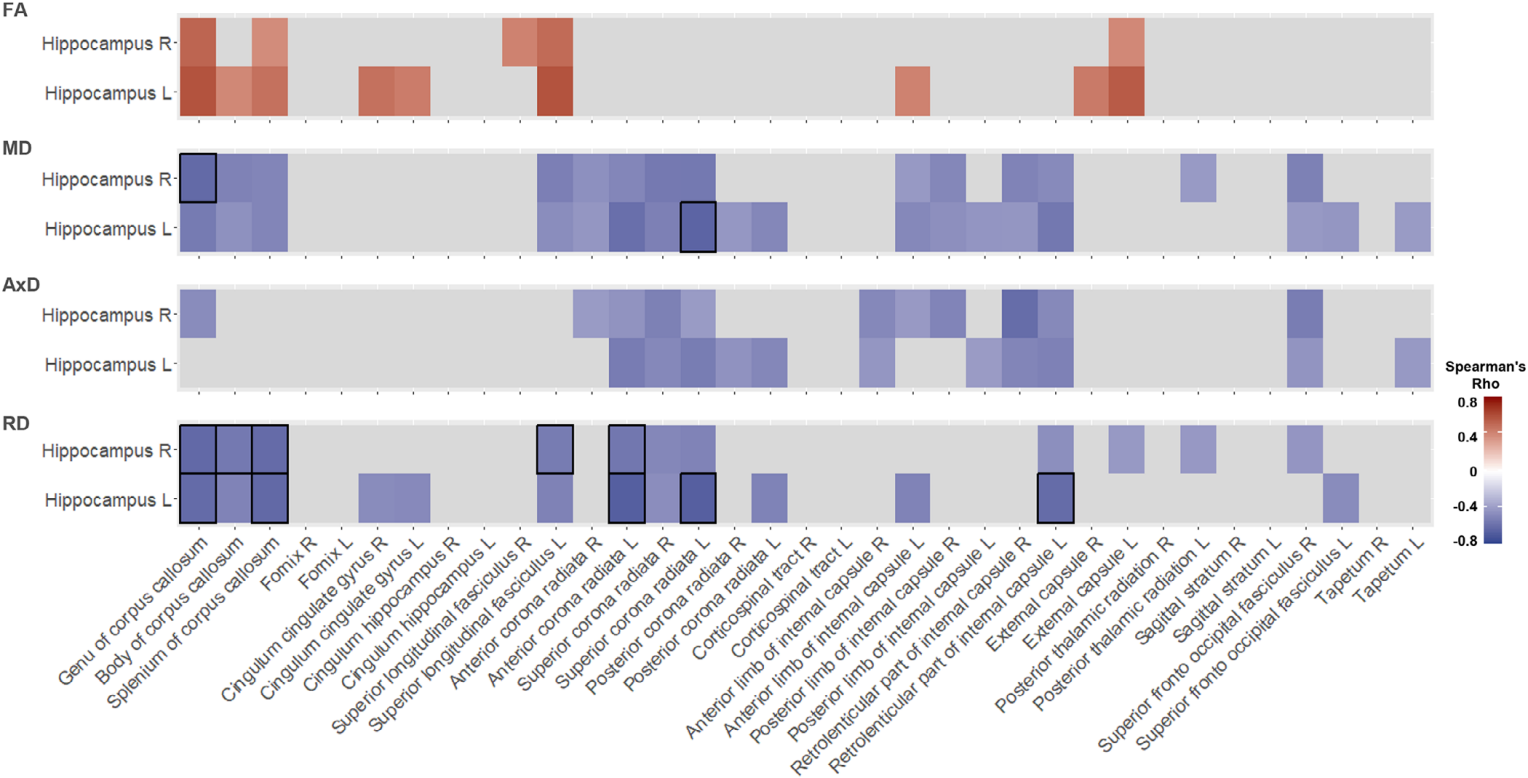
WM RD and MD are negatively correlated with hippocampal volumes. Heatmaps represent the correlations between the hippocampal volumes (*y* axis) linear model residuals for age, sex, and MRI‐to‐death interval and DTI metrics (*x* axis) linear model residuals for age, sex, b‐value, and MRI‐to‐death interval, in the ROIs from the JHU‐ICBM‐DTI‐48 WM atlas. The color bars show the Spearman correlation coefficients (rho), with red and blue scales indicating positive and negative correlations, respectively. Only significant correlations are shown, with FDR‐corrected ones outlined by a black border. AxD, axial diffusivity; DTI, diffusion tensor imaging; FA, fractional anisotropy; FDR, false discovery rate; ICBM, International Consortium of Brain Mapping; JHU, Johns Hopkins University; MD, mean diffusivity; MRI, magnetic resonance imaging; RD, radial diffusivity; ROI, regions of interest; WM, white matter

We also examined the association between WM integrity and hippocampal volumes, a recognized imaging indicator of AD pathology. Our analysis revealed statistically significant correlations between both left and right hippocampal volumes and DTI metrics across several WM ROIs. However, only some correlations involving RD and MD remained significant after applying FDR corrections. Statistical significance was limited to RD and MD in ROIs of the left hemisphere, predominantly observed in the left anterior and superior corona radiata RD correlation with the left hippocampal volume (rho = –0.65, *P* < 0.015, Spearman correlation with FDR correction), and to the genu and splenium of the corpus callosum (Figure 5). Together, these results further support the previous analyses, demonstrating that regional alterations in WM microstructure are associated with brain atrophy in AD.

## 4 DISCUSSION

The present study investigated the association between NFTs, NPs, and CAA severity with WM integrity in a cohort with pathologically defined AD. Using a ROI analysis of WM, we identified differences in DTI metrics between neuropathology‐defined groups. While NFTs and CAA severity demonstrated distinct influences on WM microstructure, their impact appears to be shaped by the presence of each other.

We found changes across multiple WM tracts between Braak stages II to IV and V/VI, most notable in the corona radiata, sagittal stratum, and tapetum. The microstructural changes detected in the fornix and sagittal stratum, which connects the occipital, temporal, and frontal lobes through fibers coming from the inferior longitudinal and fronto‐occipital fasciculus, and optic radiation,^38^ are in line with the progression of tau pathology into the neocortex at Braak stage V/VI. Furthermore, the substantial diffusivity differences observed in the corona radiata are possibly related with the progressive impairment of motor functions predominantly observed in later stages of AD.^39^ Our findings among Braak stages were also extended to the splenium of the corpus callosum. WM changes were primarily observed as increased diffusivity at Braak stage V/VI, reflected by higher MD, RD, and AxD metrics. Interestingly, these alterations were almost all detected in the left hemisphere, consistent with previous studies reporting hemispheric predominance of tau deposition in AD.^40^, ^41^ From participants with moderate to frequent NPs, WM alterations were limited to increased MD and AxD in the superior frontal‐occipital fasciculus, which was also only detected in the left hemisphere. Further supporting the concept of a possible asymmetry of the neuropathology‐influenced WM changes, we found that hippocampal volumes were mostly correlated with diffusivities in left hemisphere WM ROIs, besides the corpus callosum. Asymmetric patterns of tau accumulation have been shown to depend on which hemisphere the pathology starts in and become more pronounced as the disease progresses,^16^ resulting in worse pathological burden^40^ and heterogeneity of behavior^42^ and cognitive disease phenotypes, with left tau and Aβ deposition predominance being associated with worse language deficits.^41^, ^43^ Furthermore, an electroencephalography‐based study reported a decrease in the left inferior temporal and frontal networks activity in AD. However, they demonstrated that higher activation of right frontal networks was associated with better performance on intrinsic memory tasks only in AD and not in controls, suggesting that compensatory mechanisms may be activated to mitigate the reduced activity in left networks in AD.^44^

Previous diffusion MRI studies have identified similar WM diffusivity patterns in AD.^11^, ^14^, ^16^, ^17^, ^45^ A stronger association between Braak stages at autopsy and *ante mortem* WM integrity, compared to CERAD scores, was likewise demonstrated in prior research. Namely, while no differences were detected among the groups with non/sparse and moderate/frequent NPs, MD in the fornix and cingulum was positively corelated with Braak staging.^22^ By combining tau PET imaging with advanced diffusion MRI in amyloid‐positive participants, a study demonstrated that the neurite density index particularly in the uncinate fasciculus, cingulum, and temporal WM tracts was consistently and significantly negatively associated with tau accumulation across all Braak stages, suggesting a decrease in axonal density. In contrast, negative associations in regions such as the splenium of the corpus callosum, fornix, and the superior fronto‐occipital fasciculus emerged only at Braak stages III/IV and V/VI, suggesting that, in contrast to the other tracts, these are affected only later in the progression of the disease.^45^

Our study complements previous research examining NFT effects on WM integrity in amyloid‐positive participants by including CAA in the analysis. We found alterations in DTI diffusivity metrics across CAA groups, predominantly affecting the right sagittal stratum, but also the splenium of the corpus callosum and cingulum, in severe CAA. This posterior predominance of CAA WM effects has also been observed in previous studies, showing higher posterior/anterior ratios of WM hyperintensities,^46^, ^47^ and greater WM atrophy in posterior occipital regions^48^ in participants with CAA. Importantly, we observed that the effects of CAA severity in the WM integrity were highly coupled to NFT burden, predominantly in the splenium of the corpus callosum. However, both CAA and NFTs demonstrated preferentially driving the effects in the right and left hemispheres, respectively. CAA is commonly identified in both early‐ and late‐onset AD,^49^ and was shown to worsen and interact with both Aβ and tau pathologies to synergistically prompt cognitive decline,^24^, ^25^, ^50^ particularly in Braak stages IV to VI.^50^ Furthermore, tau pathology was recently demonstrated to be strongly associated with CAA‐related vascular markers.^51^ A histology study examining cortical regions of the parietal lobe in an AD cohort found no associations between WM lesions and CAA.^52^ However, a potential indirect effect of CAA on WM integrity through tau pathology, which was strongly associated with WM lesions, was not tested. Indeed, CAA‐mediated cognitive impairment was proven to be associated with increased WM peak skeletonized MD,^27^, ^28^, ^53^ highlighting the importance of considering the influence of CAA co‐pathology on WM degeneration in AD.

One of the most concerning side effects associated with the monoclonal antibodies targeting Aβ is an increased risk of developing ARIAs, especially ARIAs manifested as edema (ARIA‐E). CAA was shown to be a major risk for the development of ARIA‐E after anti‐Aβ therapies,^26^ which by removing Aβ from the cortical NPs, and/or from the small vessels, may overload the perivascular clearance pathways, activate inflammatory cascades, and exacerbate CAA.^23^, ^54^, ^55^, ^56^ Interestingly, a study reporting a case of aducanumab administration observed that, although the development of ARIA‐E occurred bilaterally in posterior parieto‐occipital lobes it was worse on the right hemisphere,^56^ which is aligned with the present findings showing WM differences along CAA severity predominantly in the right posterior WM ROIs. Considering the increased burden of NFTs associated with a higher CAA severity,^24^, ^25^ CAA may have strong implications for AD treatment, as the potential exacerbation of CAA after anti‐Aβ therapies may eventually further enhance disease progression. Therefore, this implies that, while the exclusion of CAA cases from anti‐Aβ therapies is safer,^57^ these may be participants who need a prompt alternative intervention, as the presence of CAA co‐pathology may lead to a faster progression of AD pathology and cognitive decline.

By comparing the different effects that NFTs, NPs, and CAA have on WM microstructure, our study underscores the importance of integrating these co‐pathologies to better characterize AD at advanced stages, beyond GM involvement. We further demonstrate that alterations in WM DTI metrics are correlated with the participants’ CDR scores and hippocampal volumes, mainly in the splenium of the corpus callosum and left superior corona radiata, supporting the concept that these WM changes may reflect a worsening of disease outcomes. We found changes mainly for diffusivities (MD, AxD, and RD) and less so for FA. However, this could be explained by its lower sensitivity to group effects in small samples.^7^ Indeed, increased MD and RD had already shown to better characterize the tau‐related WM integrity alterations, compared to FA.^17^ Previous research demonstrated that higher WM integrity, measured by global network efficiency and diffusion metrics, attenuated the effects of tau pathology on memory decline and was highly associated with hypertension, low‐density lipoprotein levels, and years of education.^58^ Therefore, focusing on improving these risk factors to enhance WM integrity could serve as a possible strategy for fostering WM resilience to pathology.

Our study has some limitations. A larger and more balanced multicenter study should be considered to confirm these findings. Confirming possible NFT–CAA–related hemisphere asymmetric WM integrity changes in amyloid‐positive participants, and how it could result in differential clinical phenotypes would be clinically very relevant. Although we recognize the lack of inter‐center data harmonization as a study limitation, the application of harmonization methods, such as ComBat,^59^ was not feasible due to the limited sample size. Harmonization approaches necessitate a substantially larger sample size to reliably estimate site‐specific effects and avoid overfitting, particularly in the context of diffusion MRI.^59^, ^60^ Additionally, the current analysis did not account for potential confounding effects of other AD co‐pathologies, such as transactive DNA‐binding protein 43, neither the influence of apolipoprotein E alleles, due to incomplete data for some participants. The adjustment for the presence of LB co‐pathology, observed in only a few participants, did not have a major impact on the neuropathological group comparisons (Table S5 in supporting information). However, it would be valuable to explore potential LB co‐pathology group differences in WM integrity, given its relevance to AD heterogeneity.^61^ The scope of this study was limited to the CERAD semiquantitative assessment of neuritic plaques because the Thal phase, which reflects the distribution of amyloid deposition including both diffuse and neuritic plaques,^62^ was not available for this cohort. In addition, DTI approaches have relevant weaknesses to be noted, because DTI cannot accurately characterize the underlying tissue microstructure in highly heterogeneous regions, such as voxels containing crossing fibers, and lacks high specificity to the underlying biological process, such as axonal and myelin damage or inflammation.^7^, ^63^ Therefore, complementing these DTI analysis with other advanced diffusion MRI techniques, such as by capturing non‐Gaussian diffusivity using diffusion kurtosis imaging^64^ in combination with axonal biophysical modeling, would provide greater sensitivity and specificity to the biological underpinnings of diffusivity changes.^63^

In summary, with this work we demonstrated that advanced stages of NFT accumulation lead to an increase in DTI‐assessed diffusivities along different WM ROIs in AD, and that the severity of CAA co‐pathology plays an important role in the observed NFT effects, while also contributing to increased diffusivities across other ROIs.

## CONFLICT OF INTEREST STATEMENT

T.G.O. has been a consultant for Sonae and Guidepoint, has received fees as a speaker from Eisai, and has had conference fees covered from Roche and Lilly. The remaining authors have no disclosures to report. Author disclosures are available in the supporting information.

## CONSENT STATEMENT

All participants provided written informed consent at each local ADRC, and the study protocols were approved by the respective institutional review boards. The participants that contributed to the Neuropathology Data Set gave consent for autopsy.

## Supporting information



Supporting Information

Supporting Information
