# Supplementary material for: White matter microstructure is differentially impacted by cerebral amyloid angiopathy, neurofibrillary tangles, and neuritic plaque co‐pathology
Source: Alzheimers Dement. 2025 Oct 14;21(10):e70637. doi: 10.1002/alz.70637 (PMC12519507; doi:10.1002/alz.70637)
Supplement: Supplementary file 1 — Supporting Information [file ALZ-21-e70637-s001.docx]

**Supplementary Figures**

**
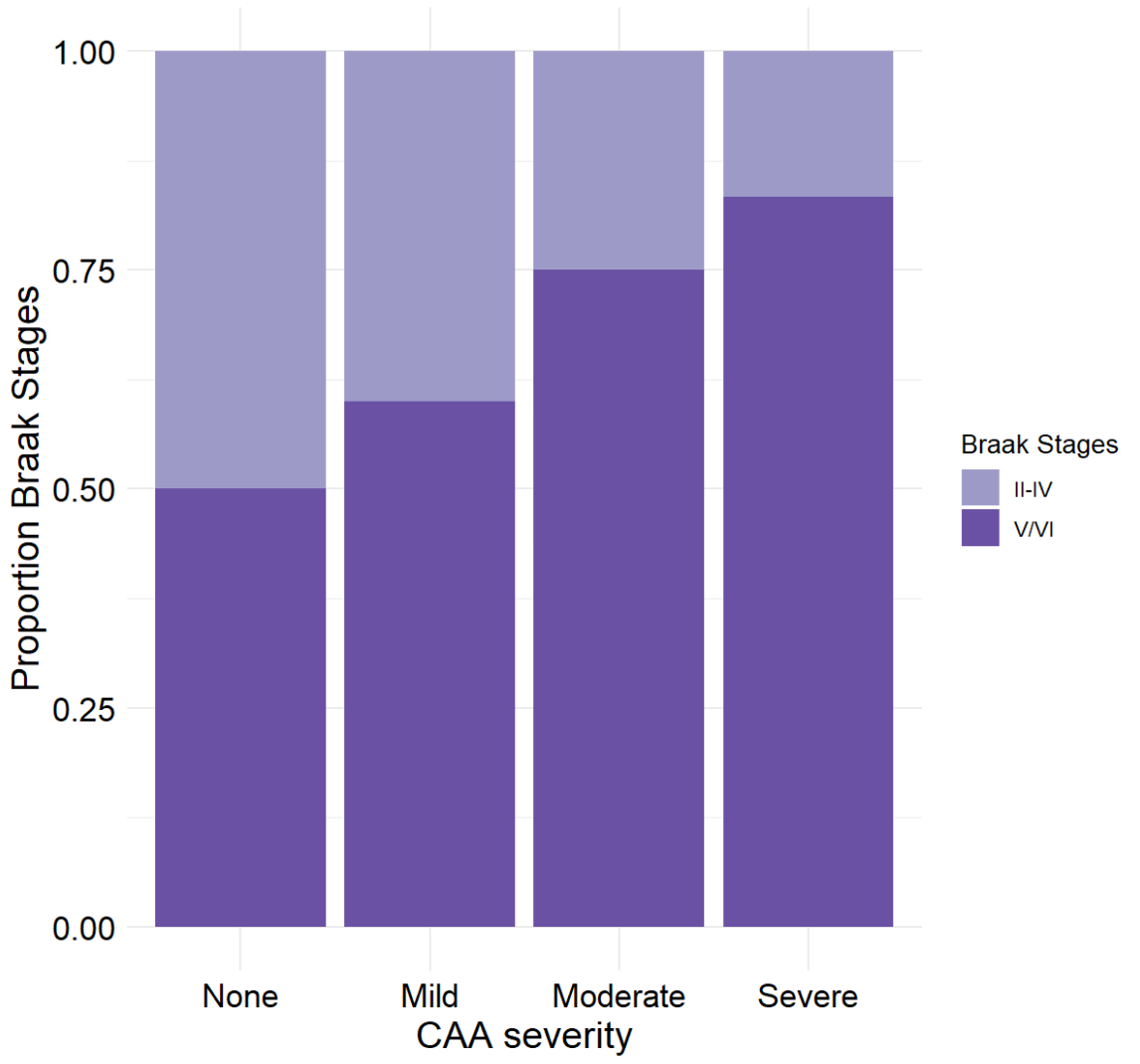
**

**Supplementary Figure 1 – Proportion of Braak stages for the different CAA severity groups.** Supplementary Figure 1 illustrates the distribution of Braak stages across each CAA group. The proportion of individuals in Braak stages V/VI increased with the severity of CAA, representing the most common stages in the mild (60%), moderate (75%), and severe (83%) NP groups.


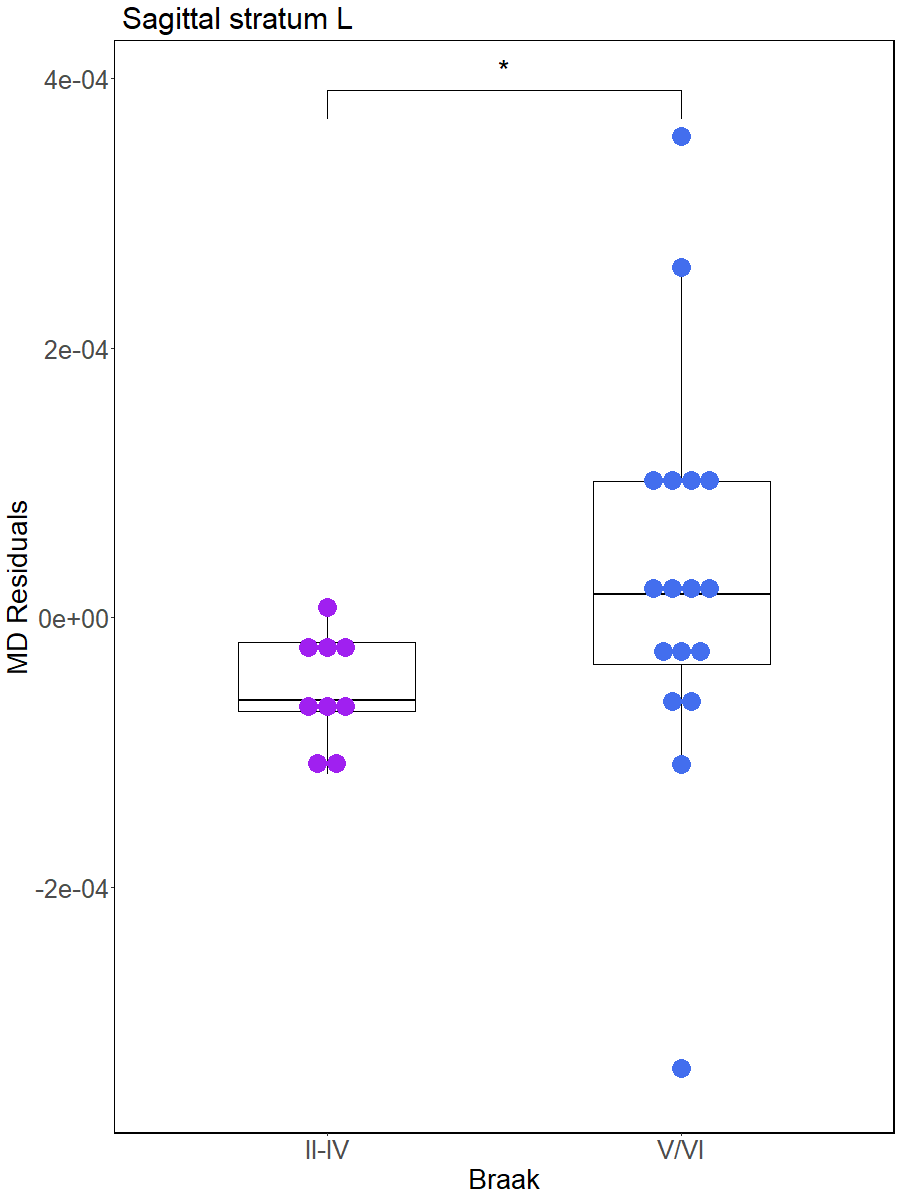

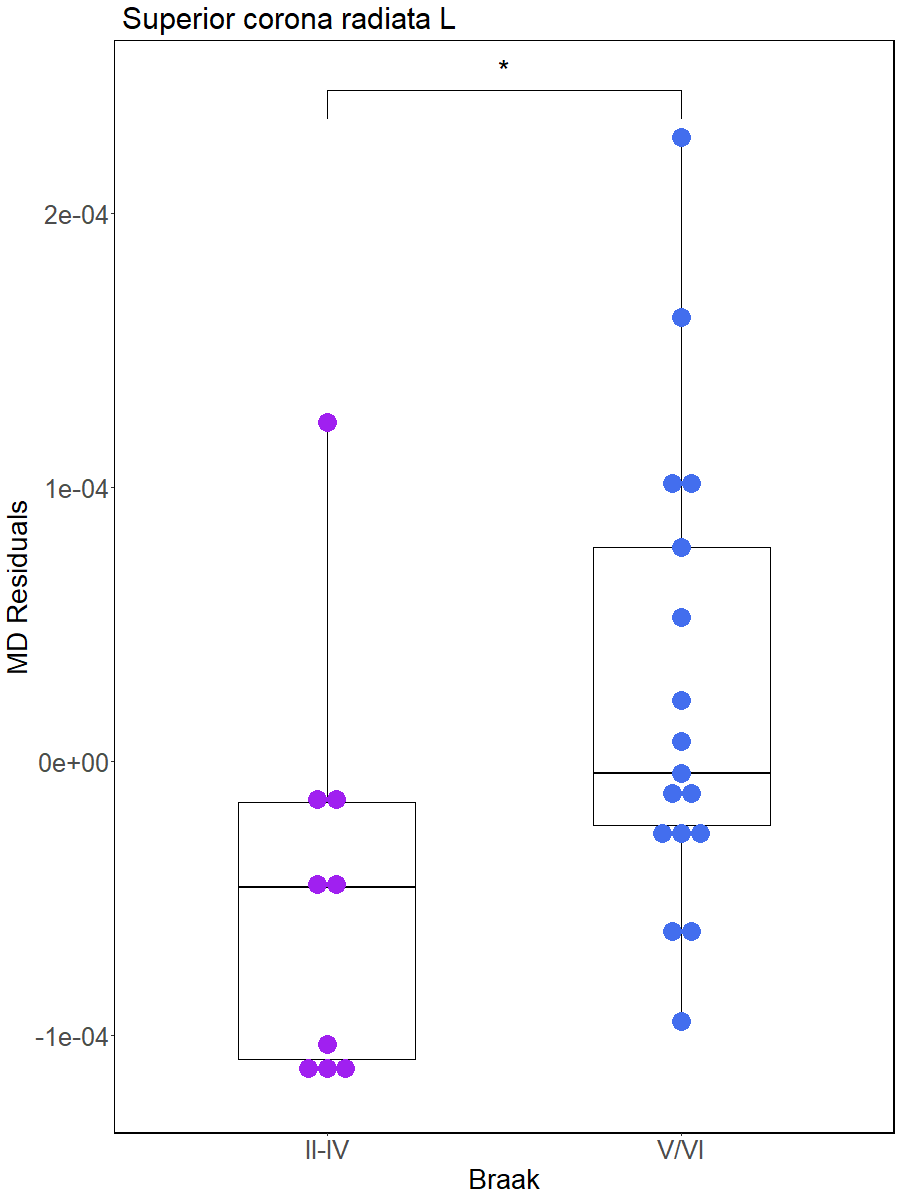

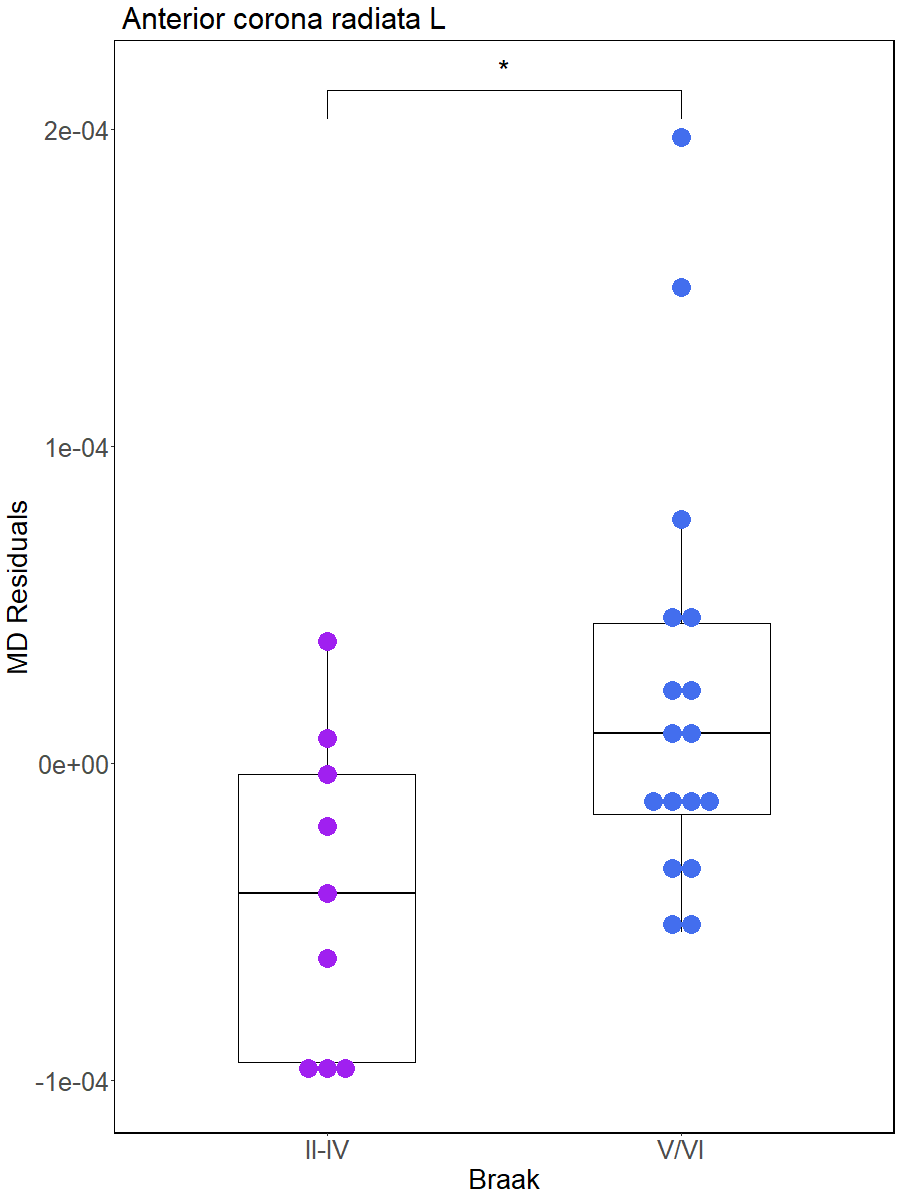

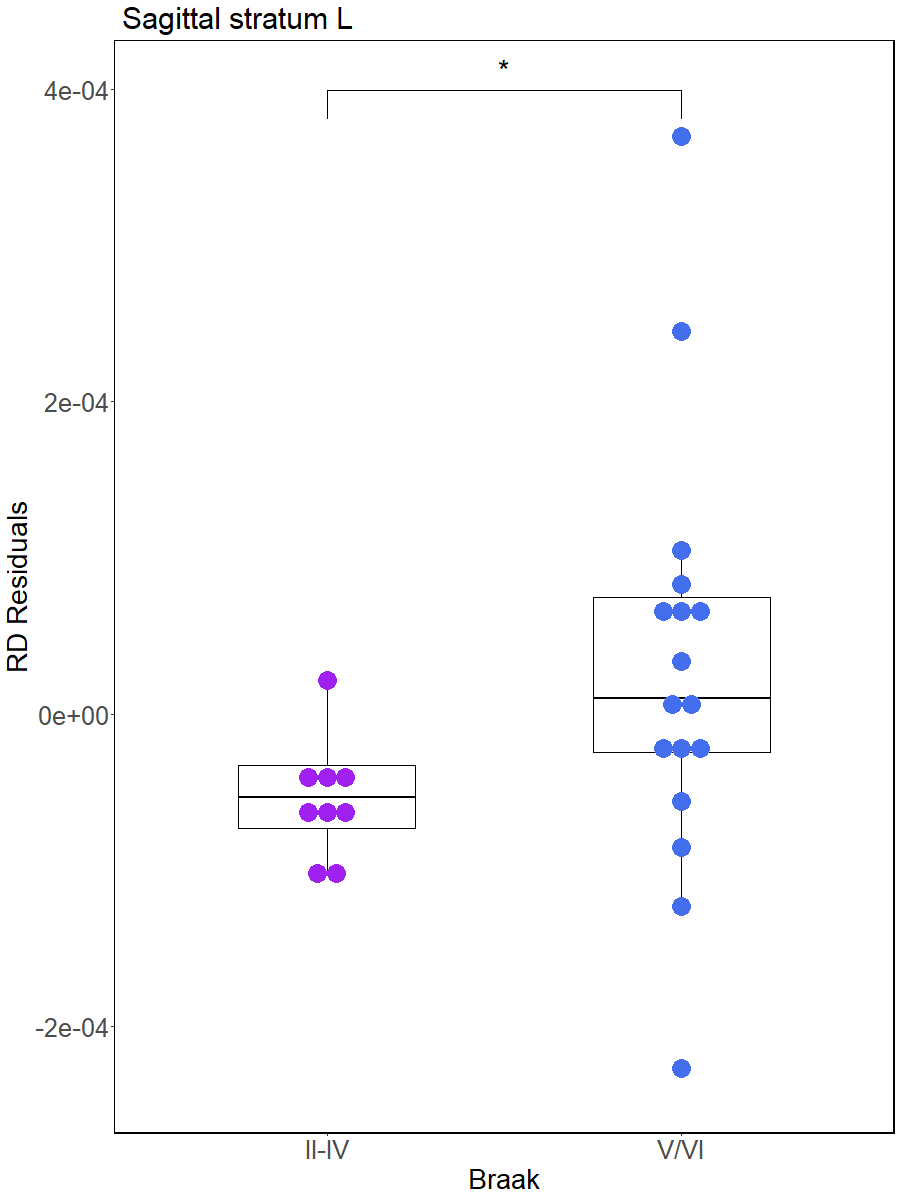

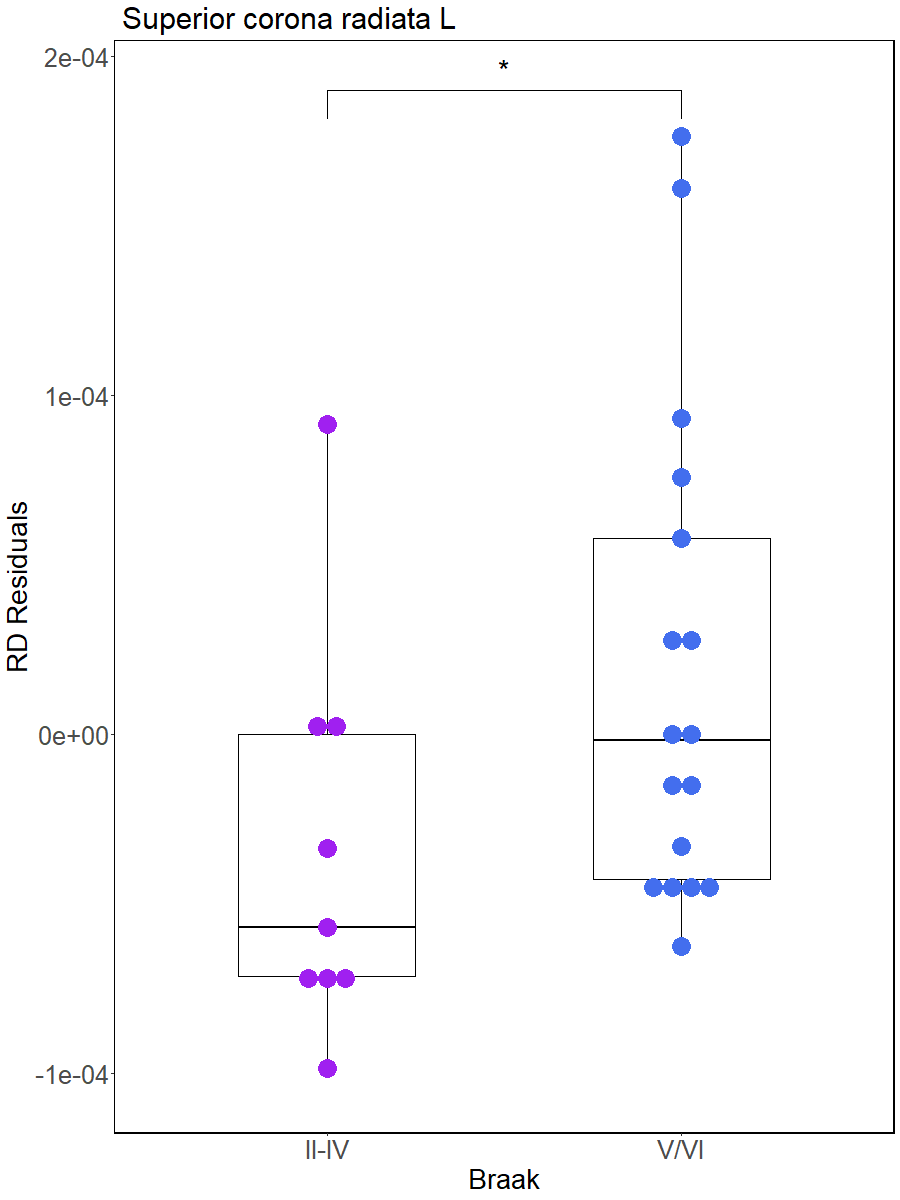

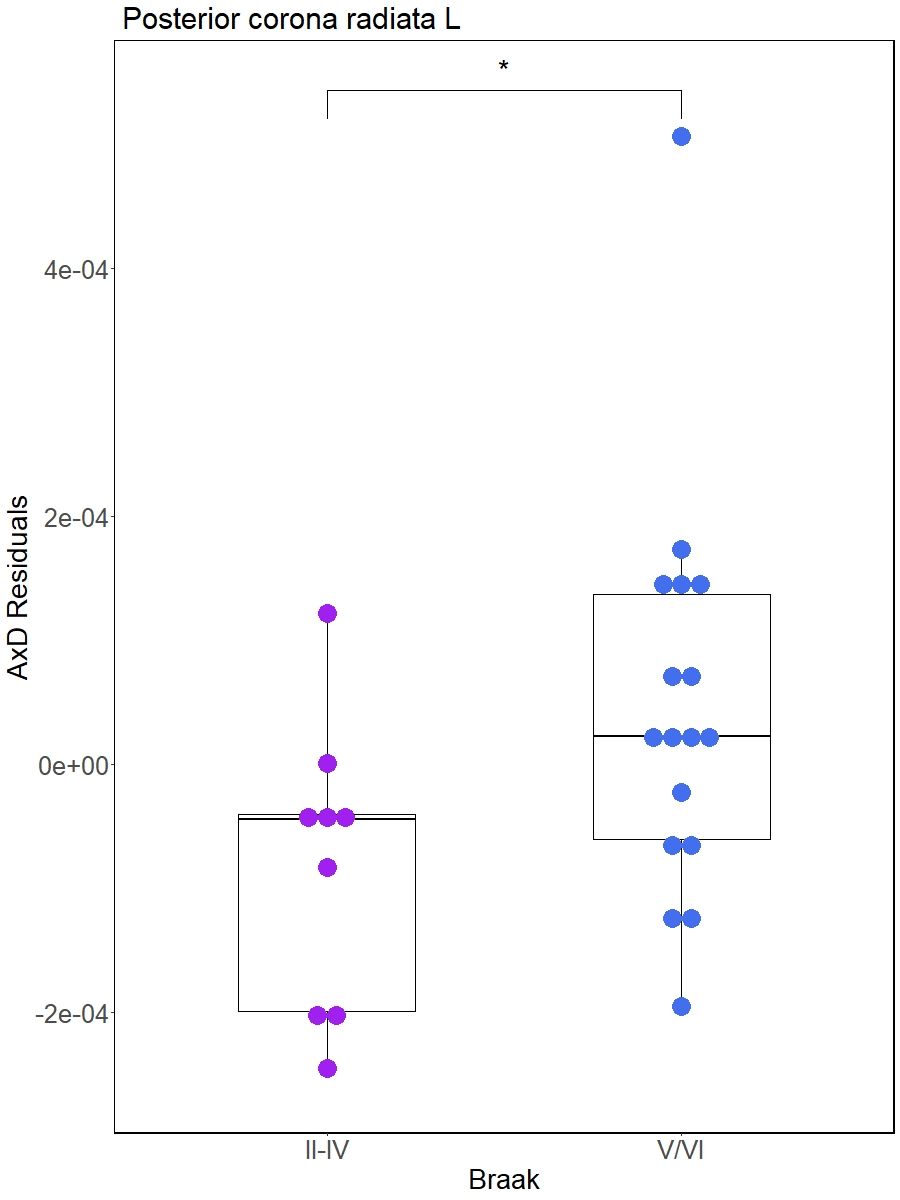

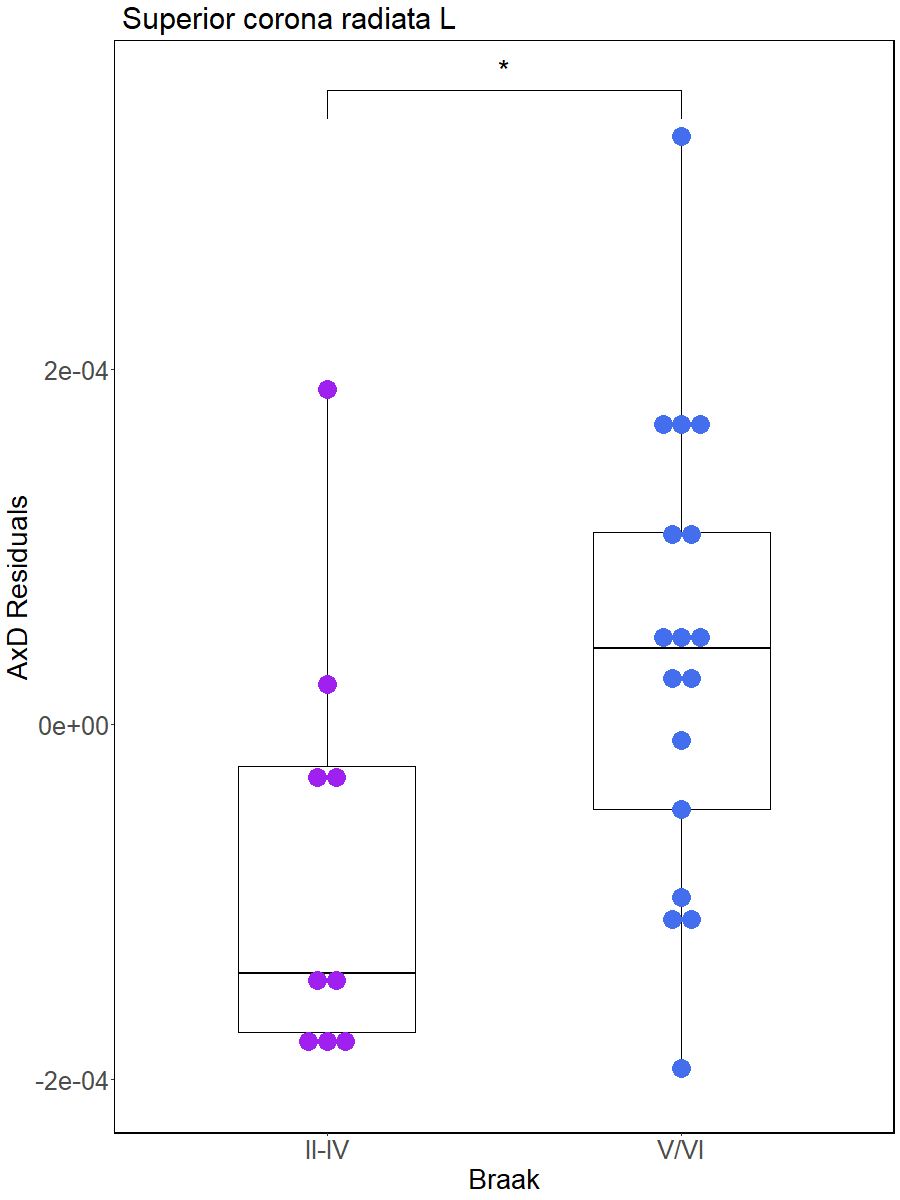

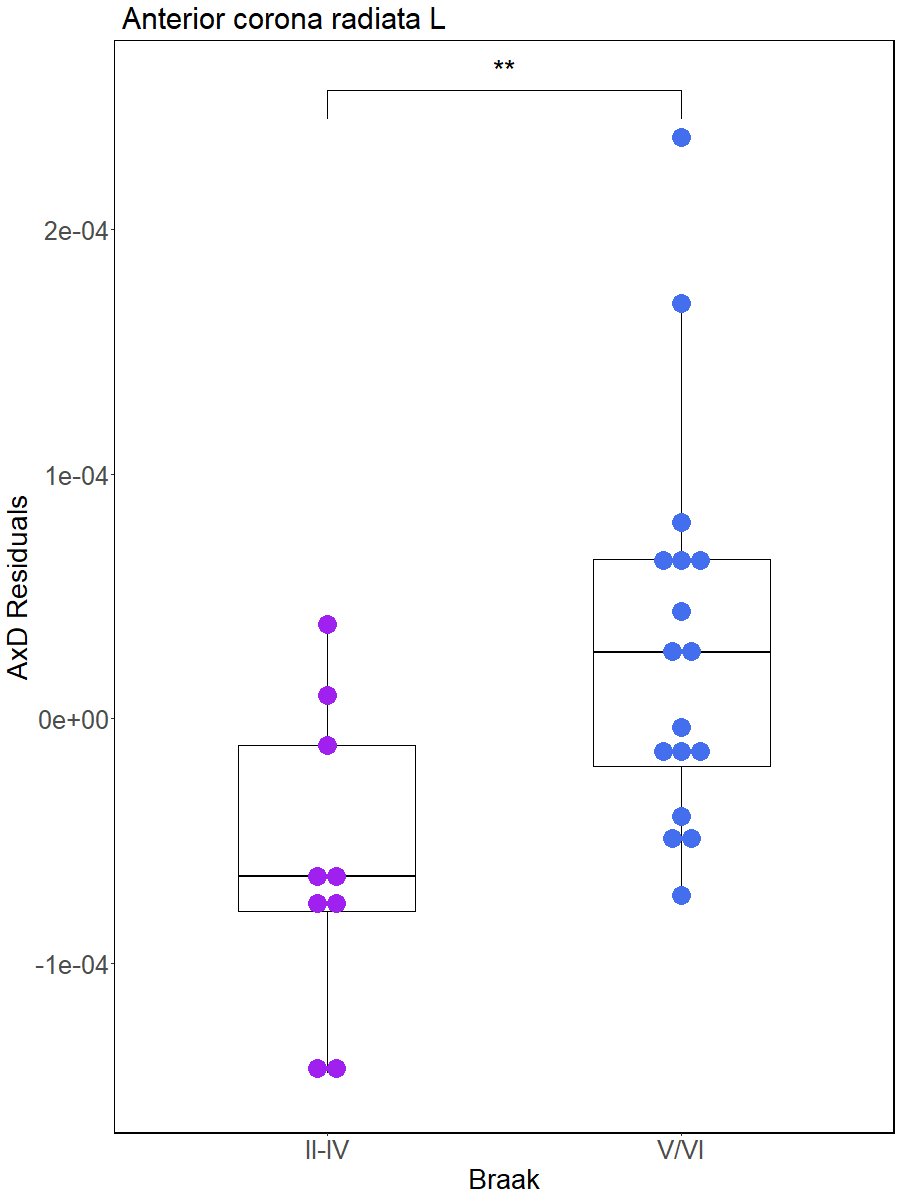

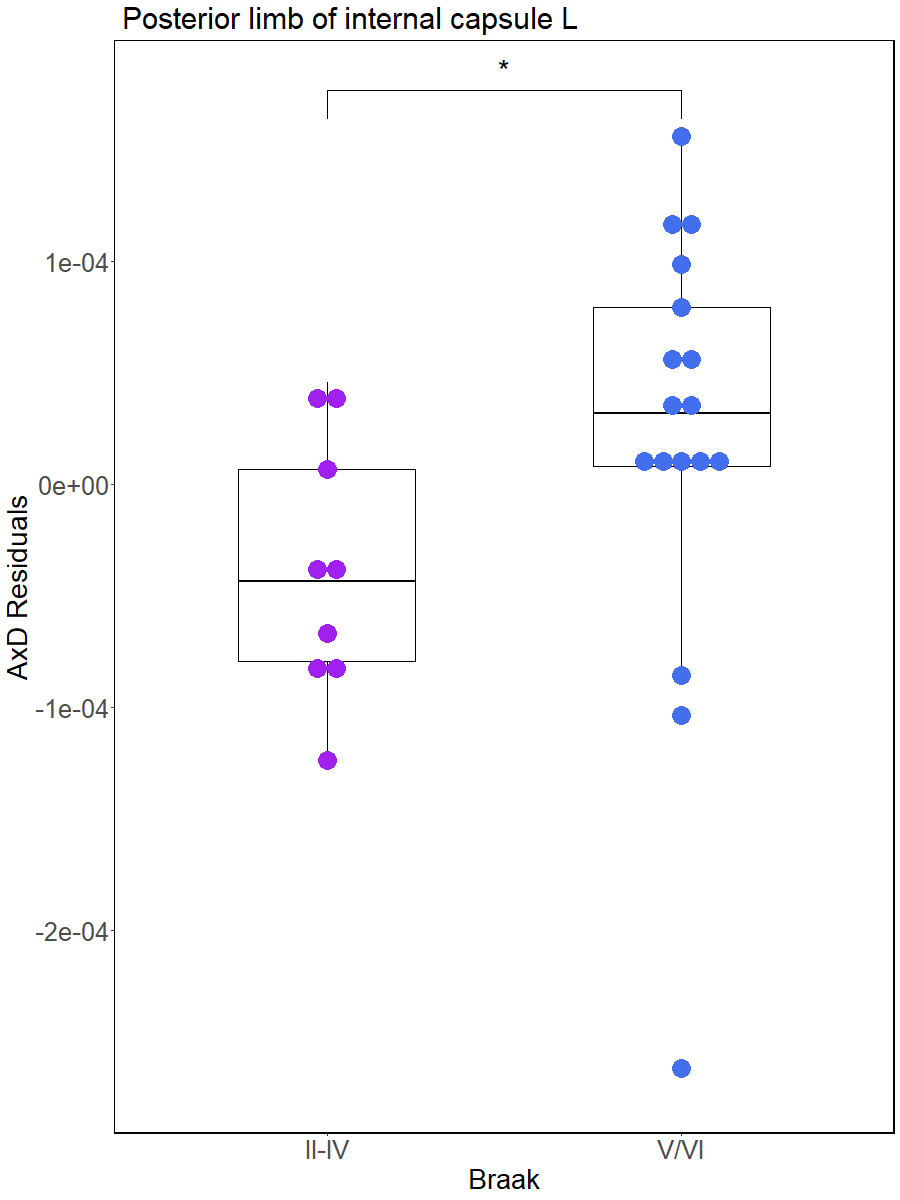

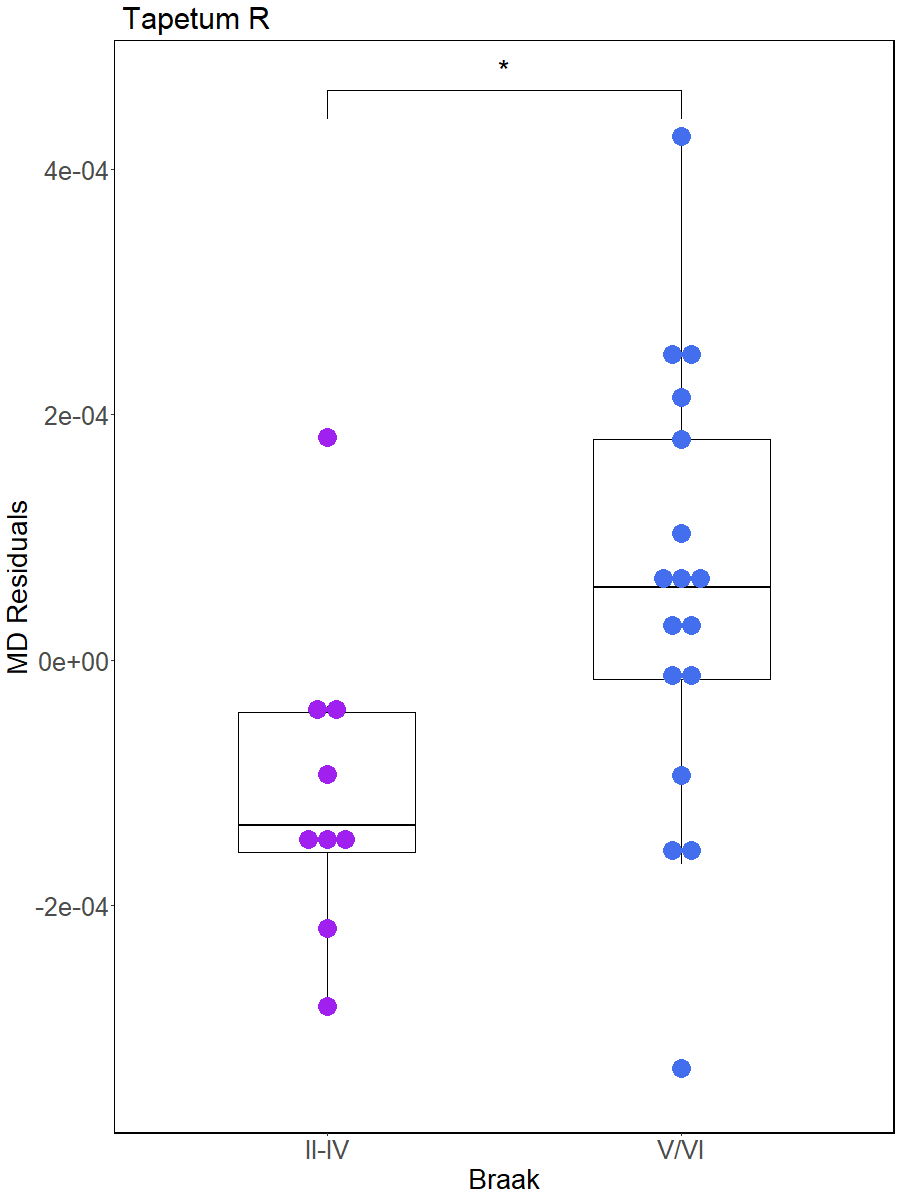

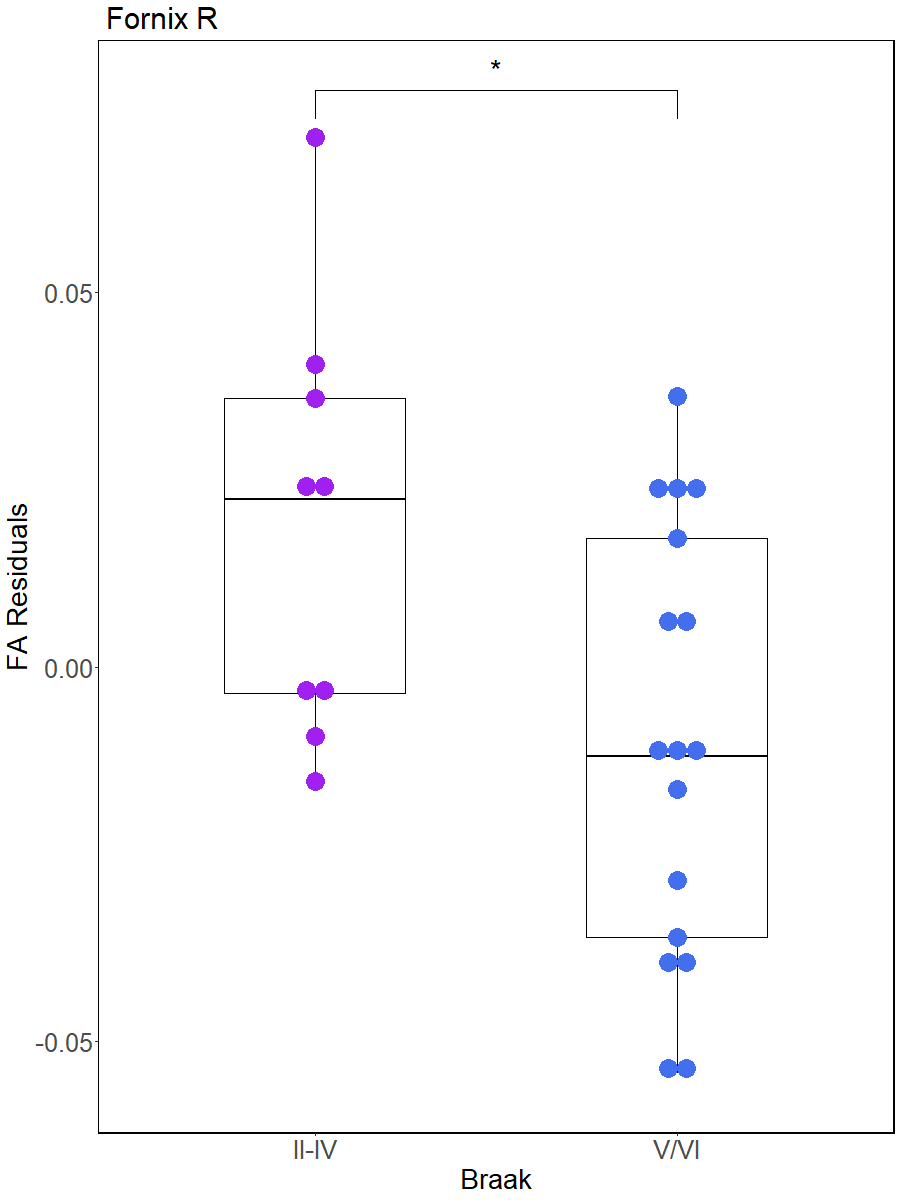

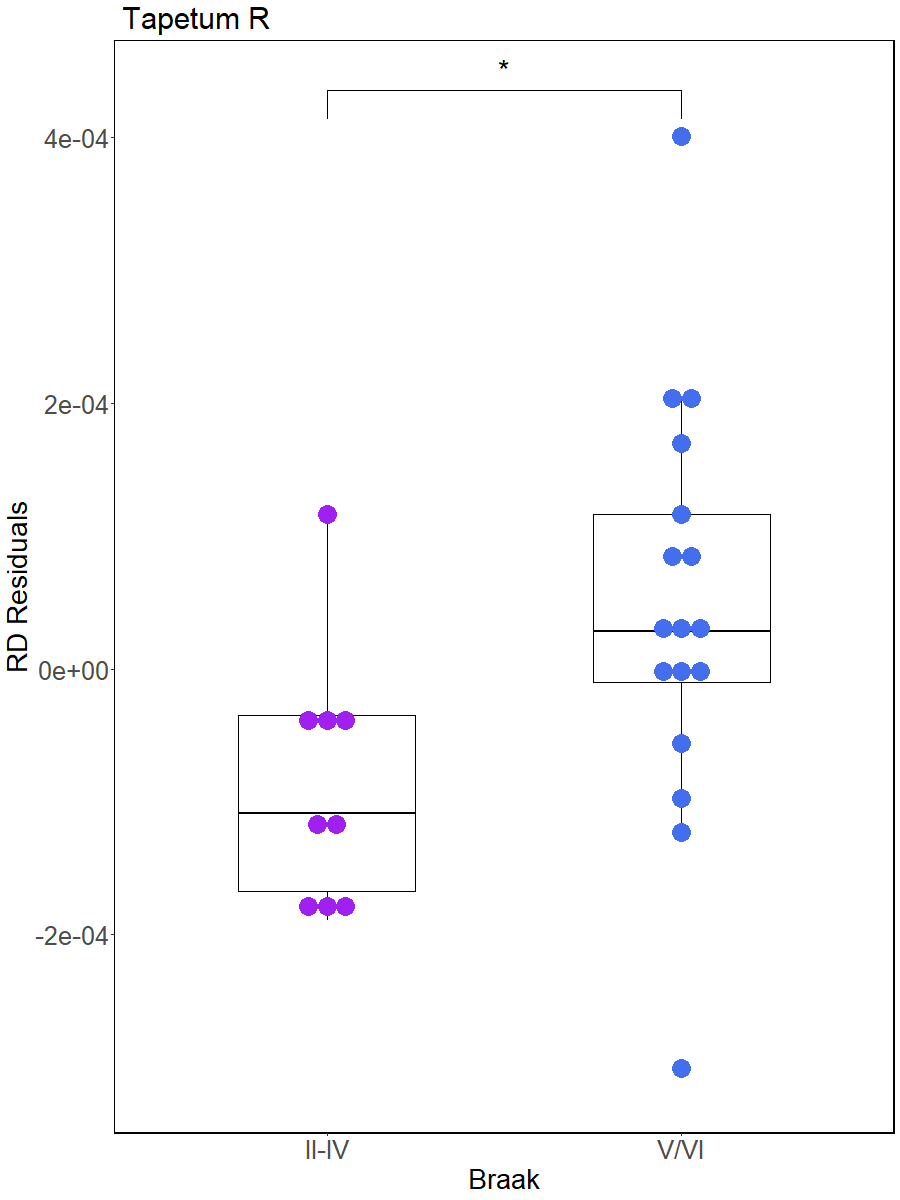

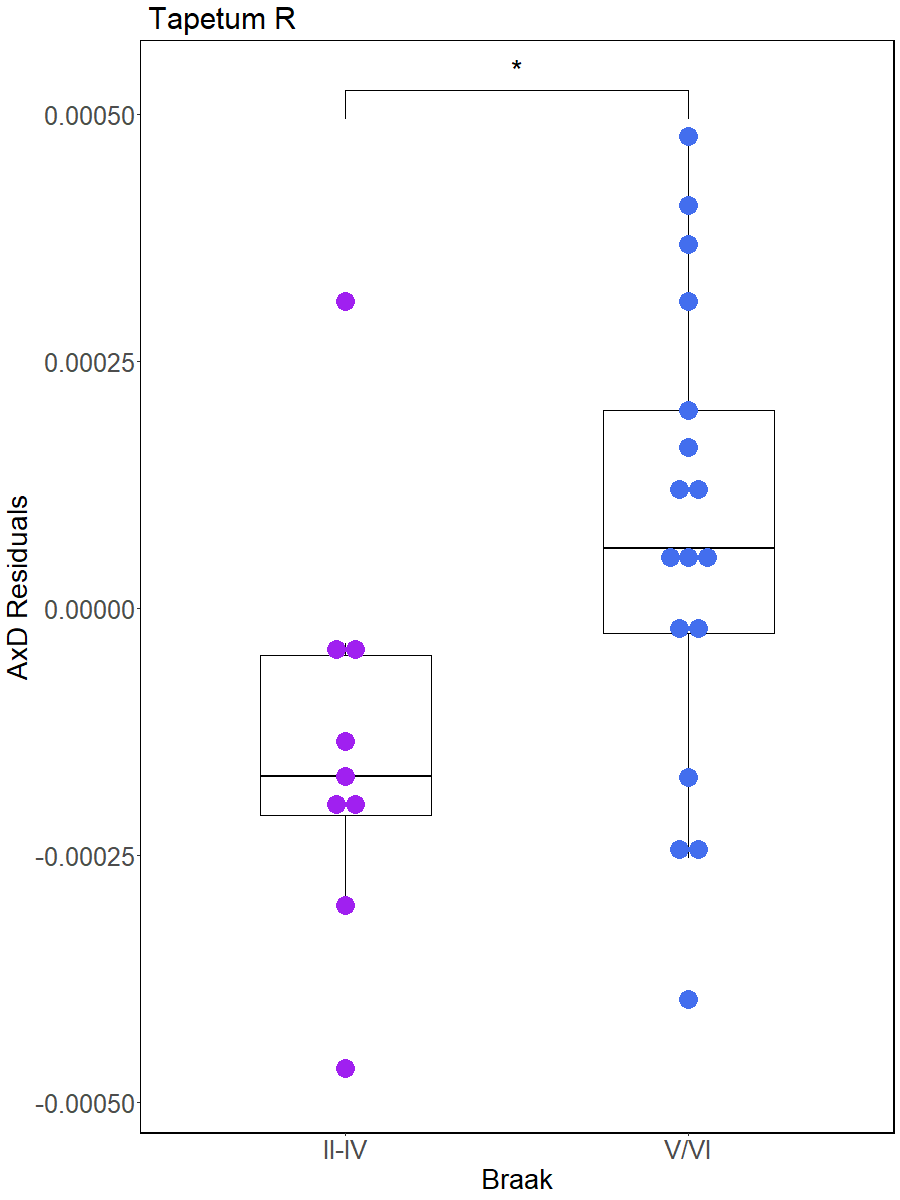

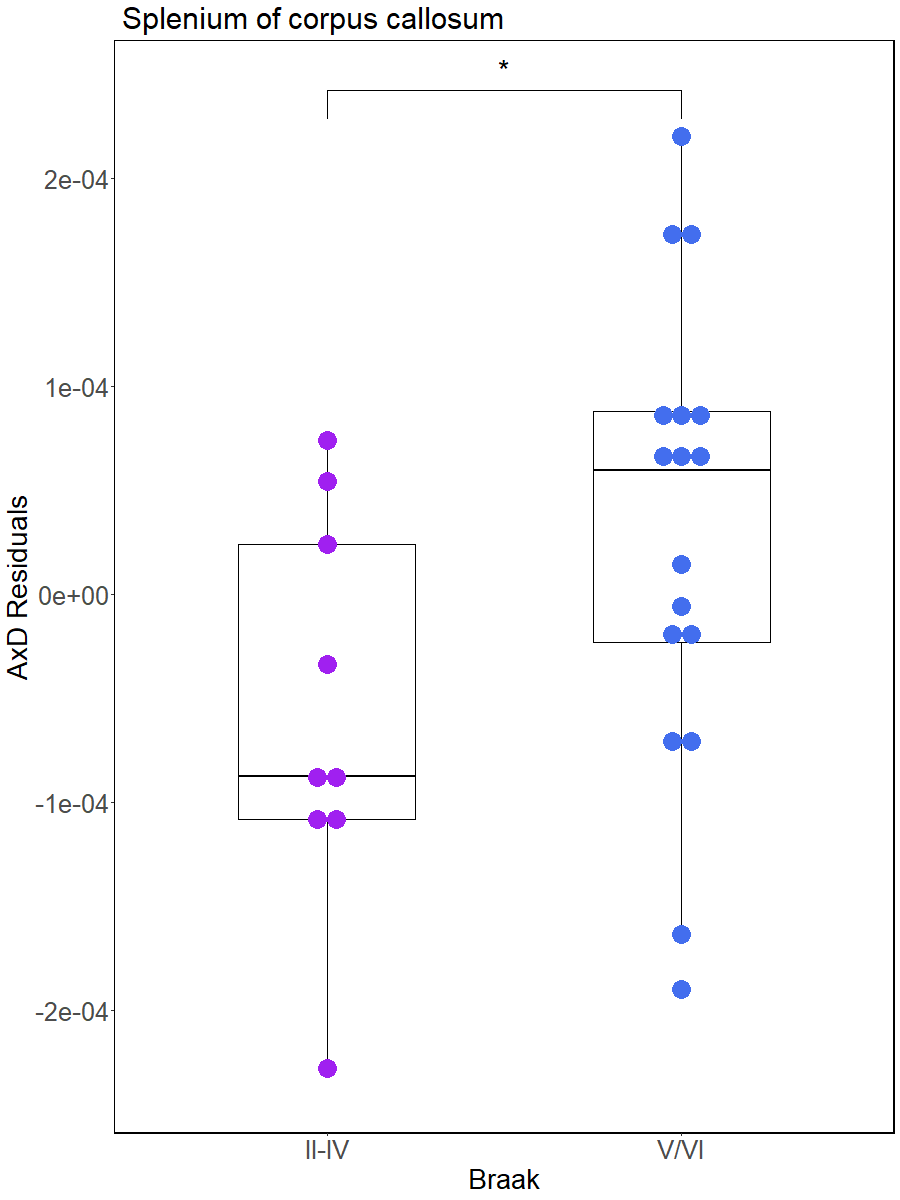


**Supplementary Figure 2 - Differences in regional WM DTI metrics between Braak stages.** Supplementary Figure 2 shows the comparisons of DTI metrics linear model residuals for age, sex, b-values and MRI-to-death interval, between Braak stages II-IV and V/VI. The JHU-ICBM-DTI-48 white-matter atlas was used to extract regional DTI metrics for each subject. Only plots for the regions and respective scalars showing statistically significant group differences (p < 0.05, Wilcoxon test) are shown. None of these comparisons remained statistically significant after Benjamini-Hochberg false discovery rate corrections. Statistical significance is denoted by p <0.05 and p<0.01.


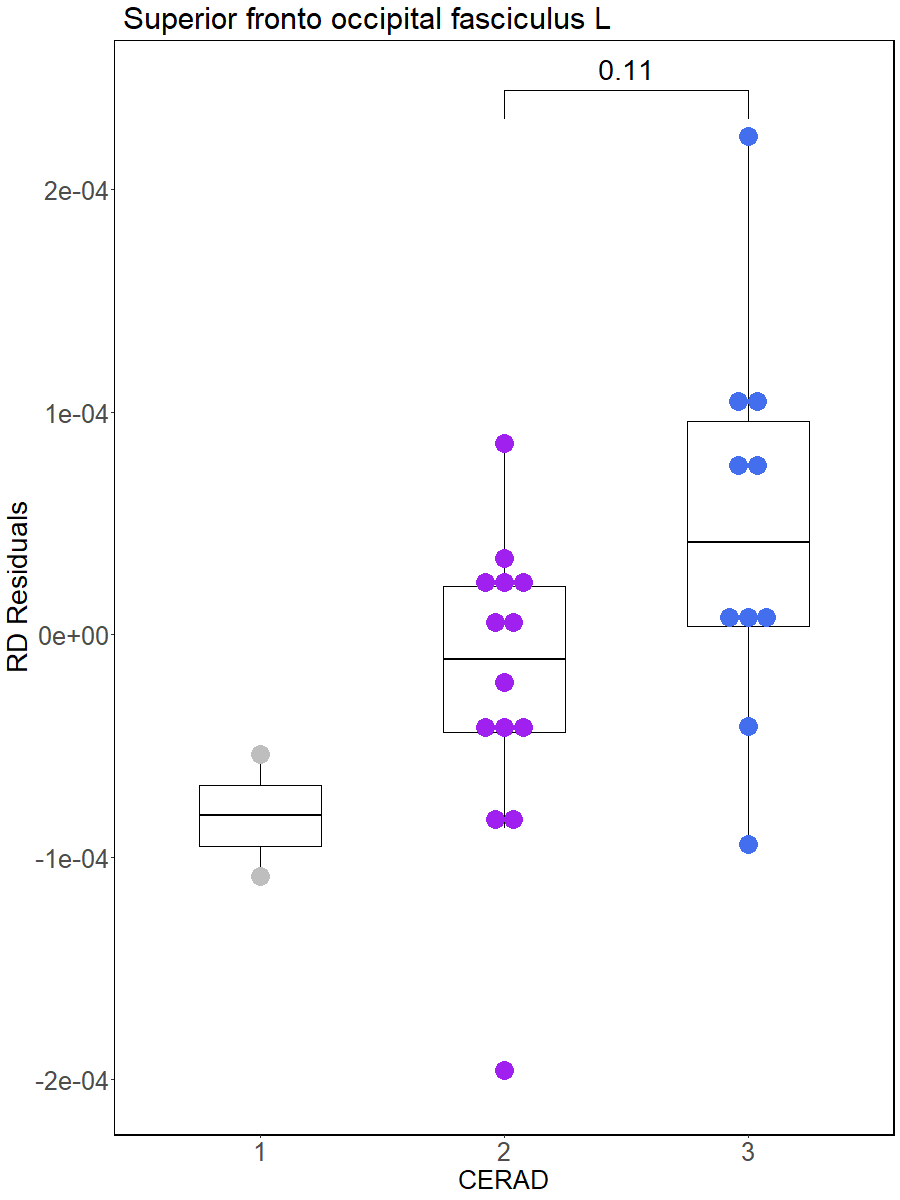

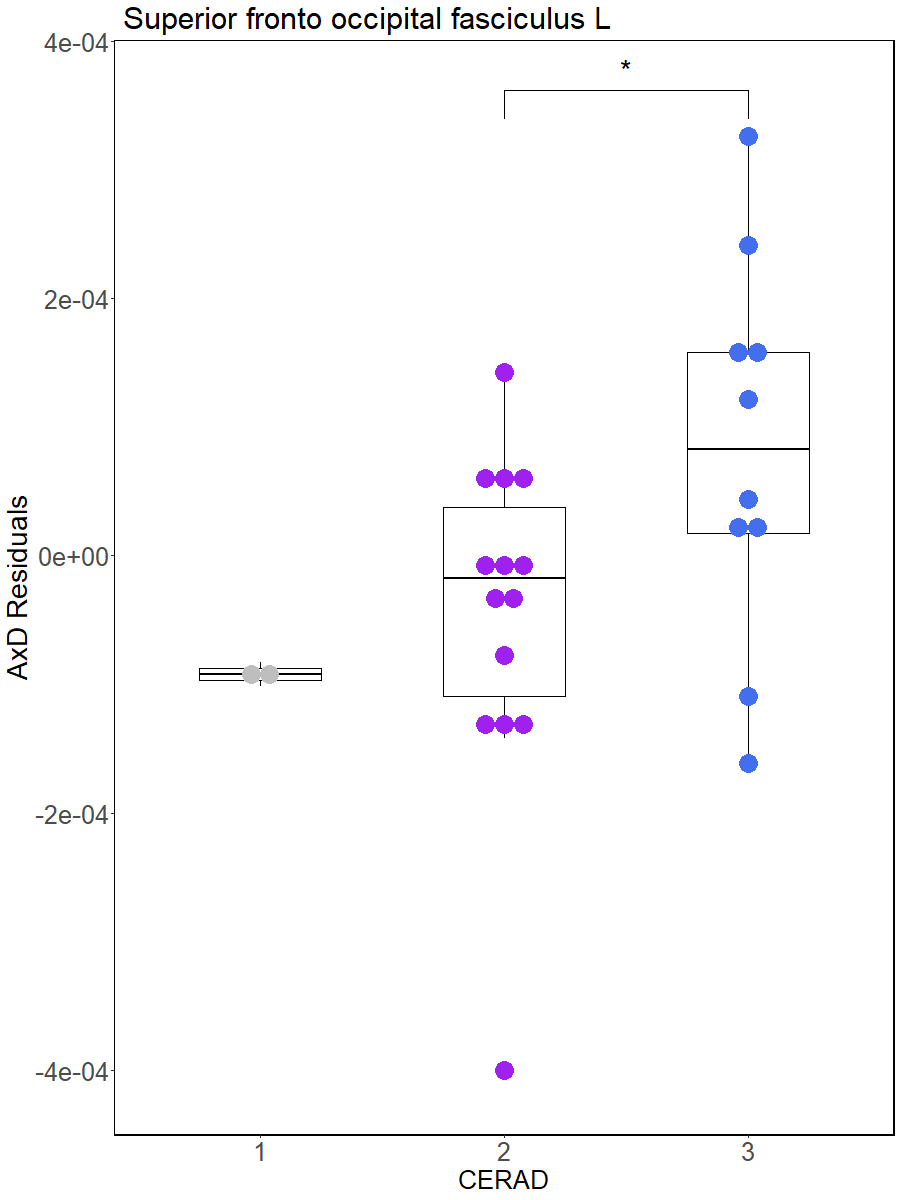

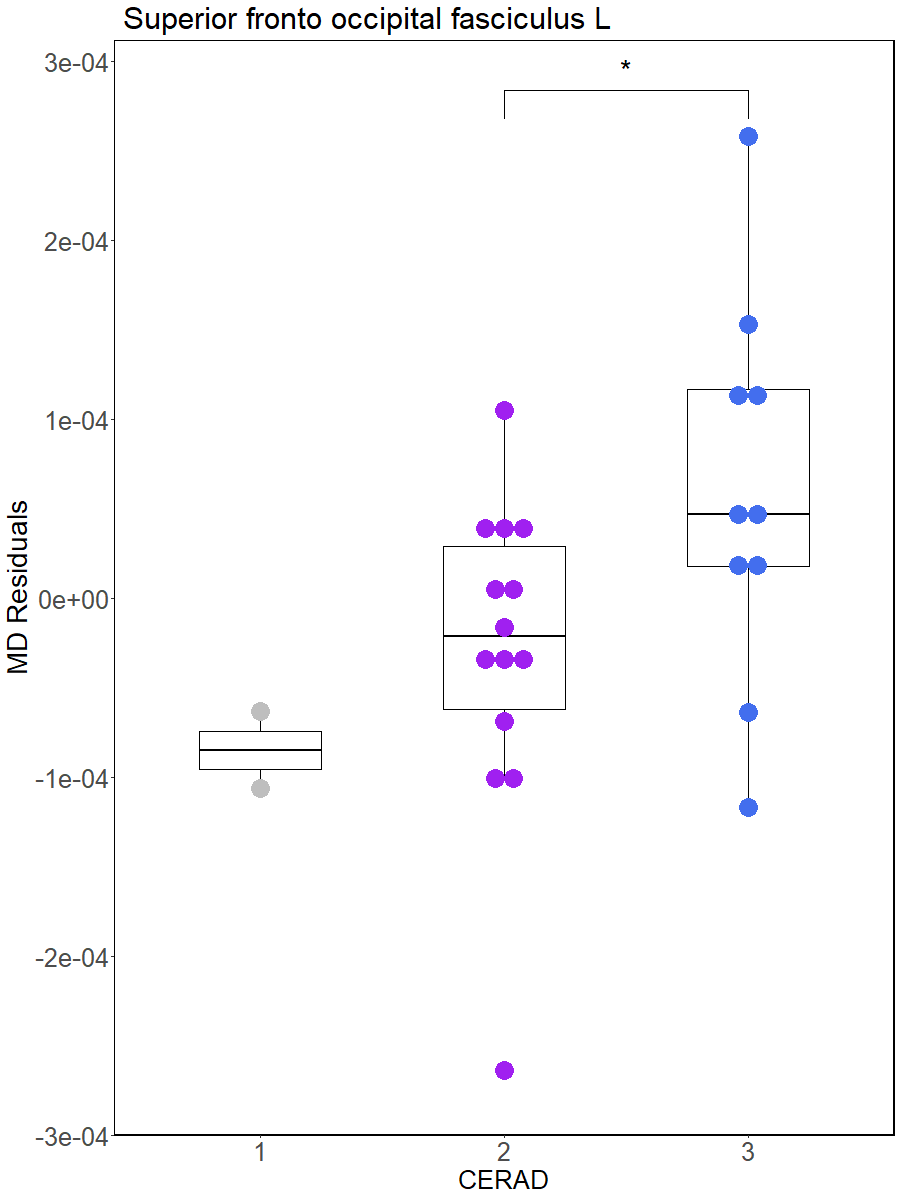

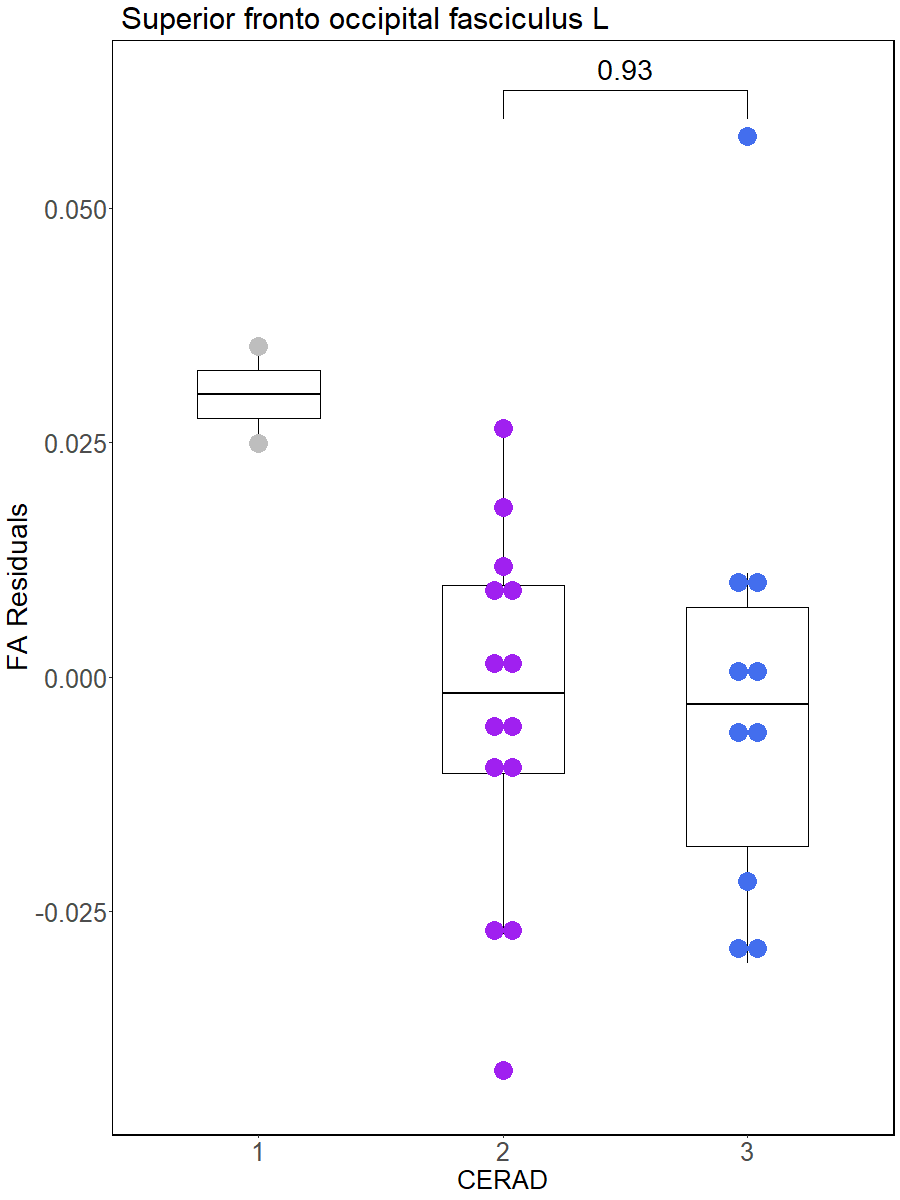


**Supplementary Figure 3 – Differences in frontal-occipital fasciculus DTI metrics between CERAD scores.** Supplementary Figure 3 shows the comparisons of DTI metrics linear model residuals for age, sex, b-values and MRI-to-death interval, between CERAD scores 2 and 3. The JHU-ICBM-DTI-48 white-matter atlas was used to extract regional DTI metrics for each subject. Only plots for the ROI showing statistically significant group differences (p < 0.05, Wilcoxon test) are presented. None of these comparisons remained statistically significant after Benjamini-Hochberg false discovery rate corrections. No comparisons were made with CERAD 1 due to the very low sample size. Statistical significance is denoted by p <0.05.

**Supplementary Figure 4- Differences in regional WM DTI metrics across CAA severity groups.** Supplementary Figure 4 shows the comparisons of DTI metrics linear model residuals for age, sex, b-values and MRI-to-death visit interval between groups with different CAA severities. The JHU-ICBM-DTI-48 white-matter atlas was used to extract regional DTI metrics for each subject. Only plots for the regions and respective scalars showing statistically significant group differences (p < 0.05 using the Kruskal-Wallis test followed by Dunn’s post-hoc test) are shown. Statistically significant comparisons are denoted by p <0.05 and p<0.01.


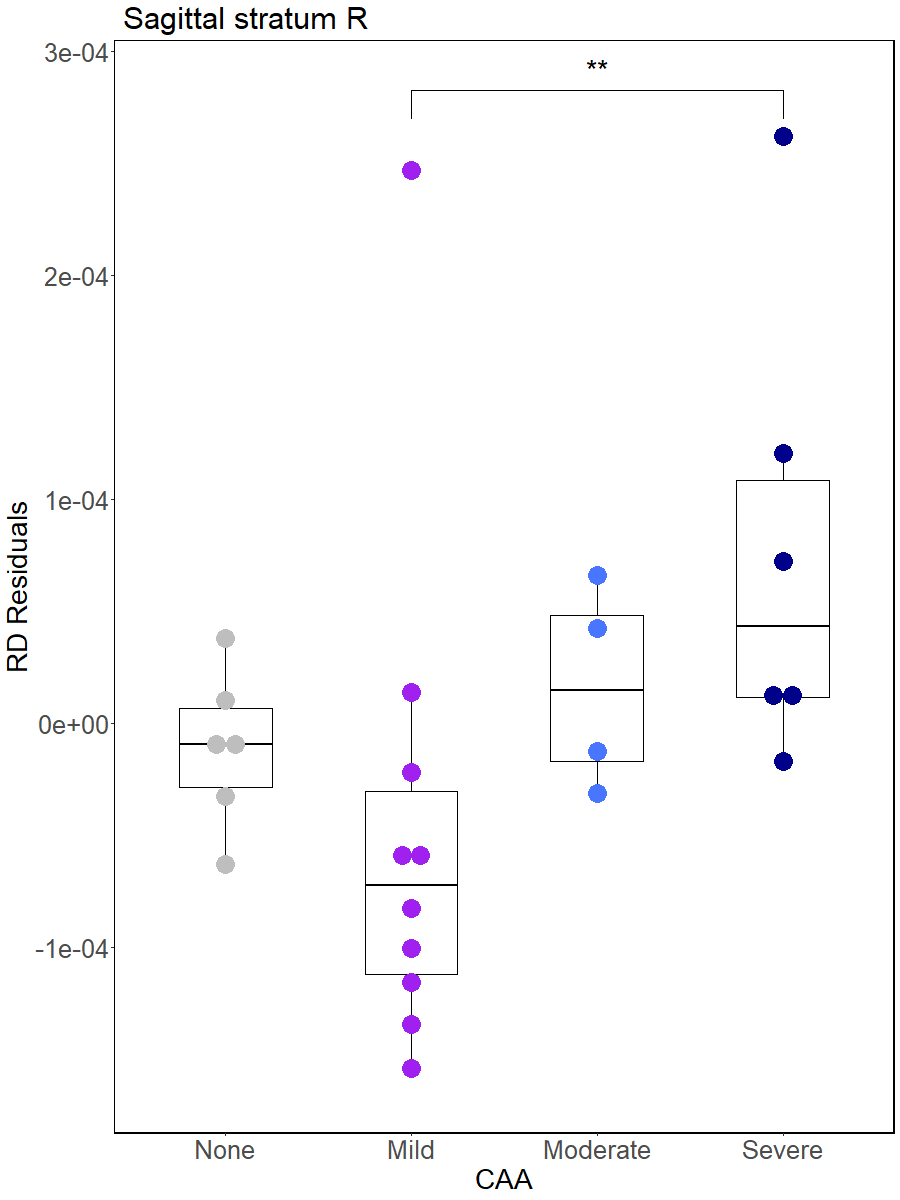

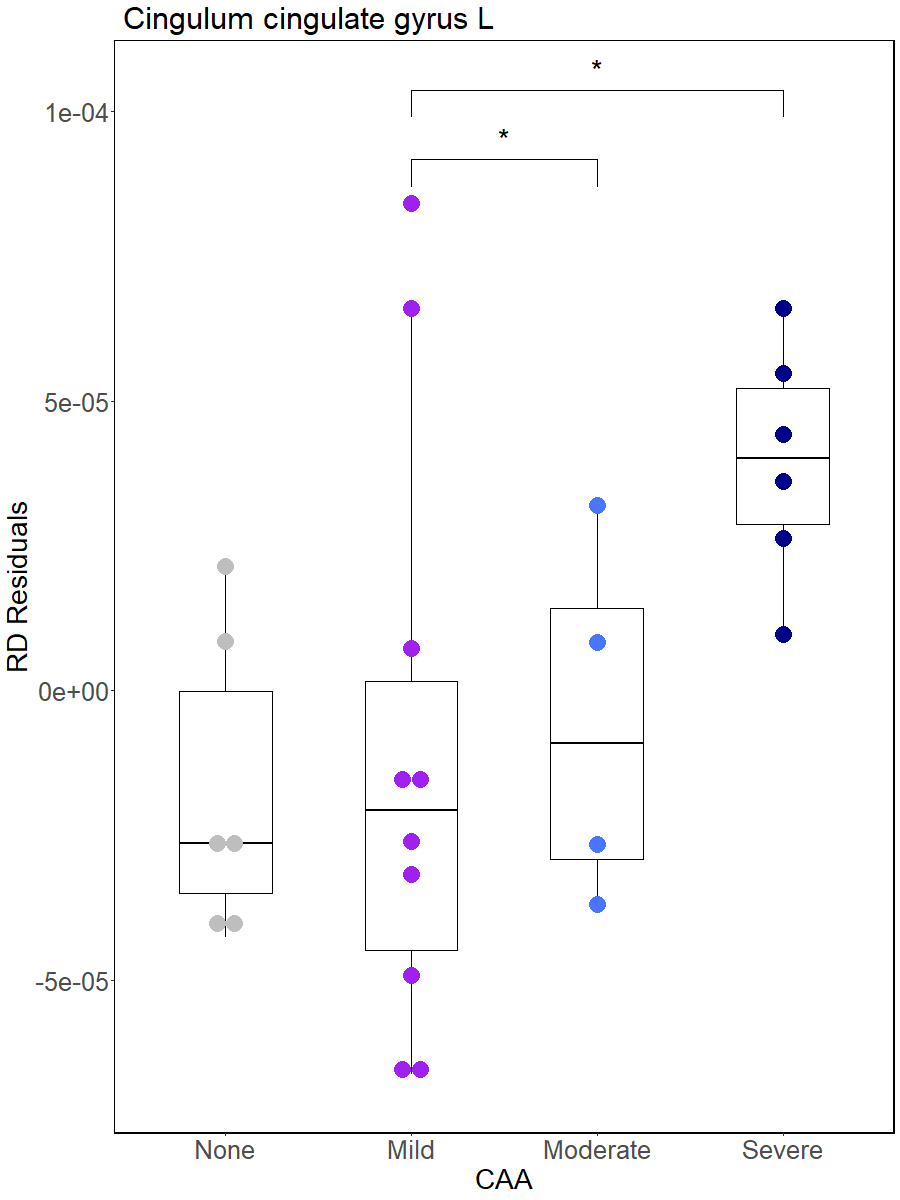

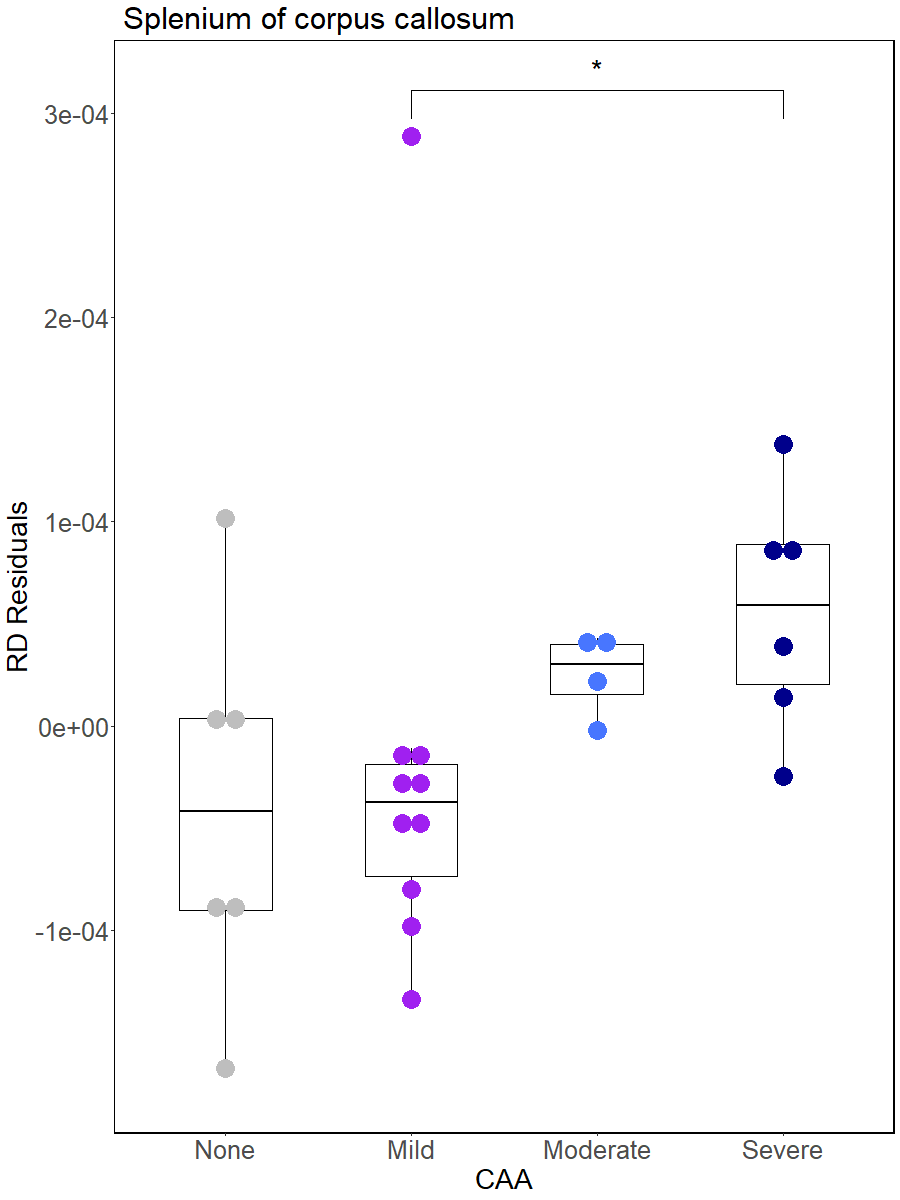

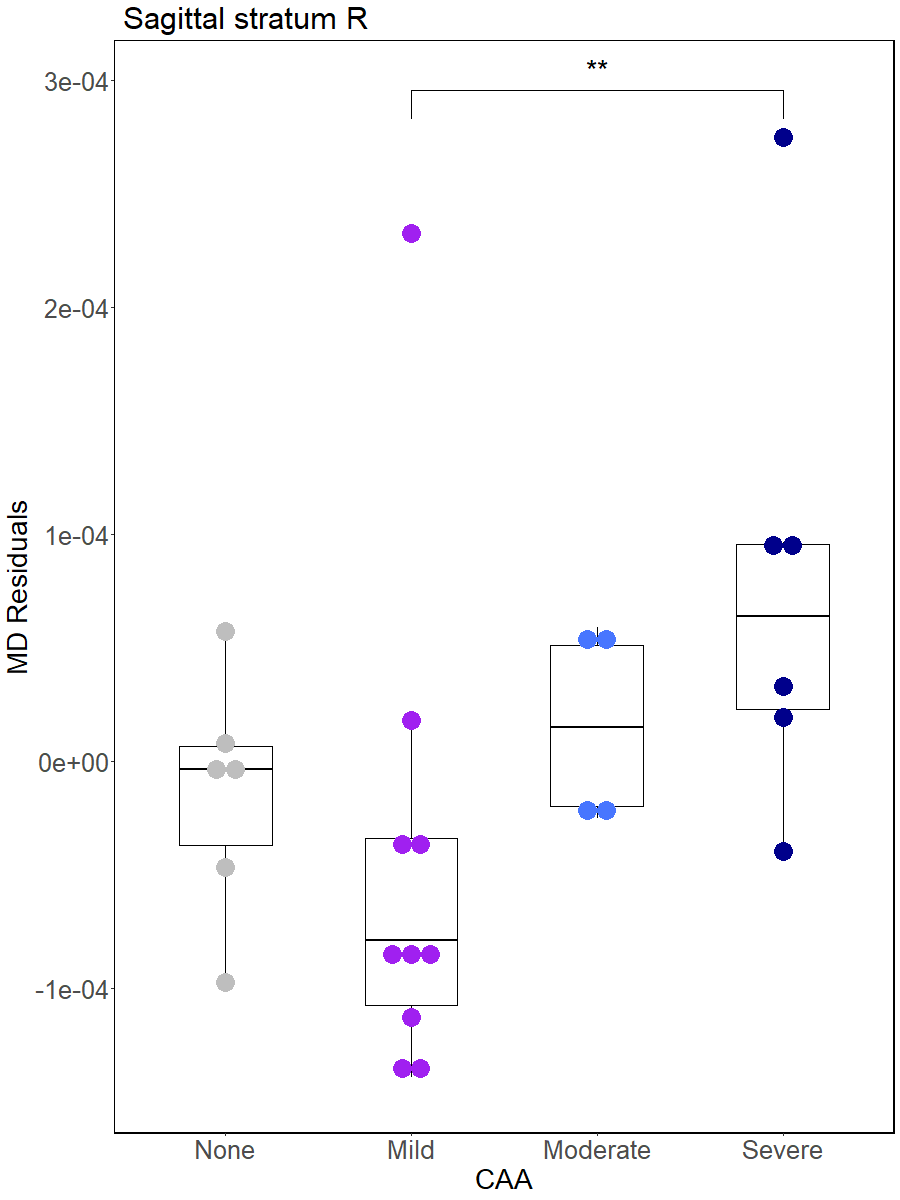

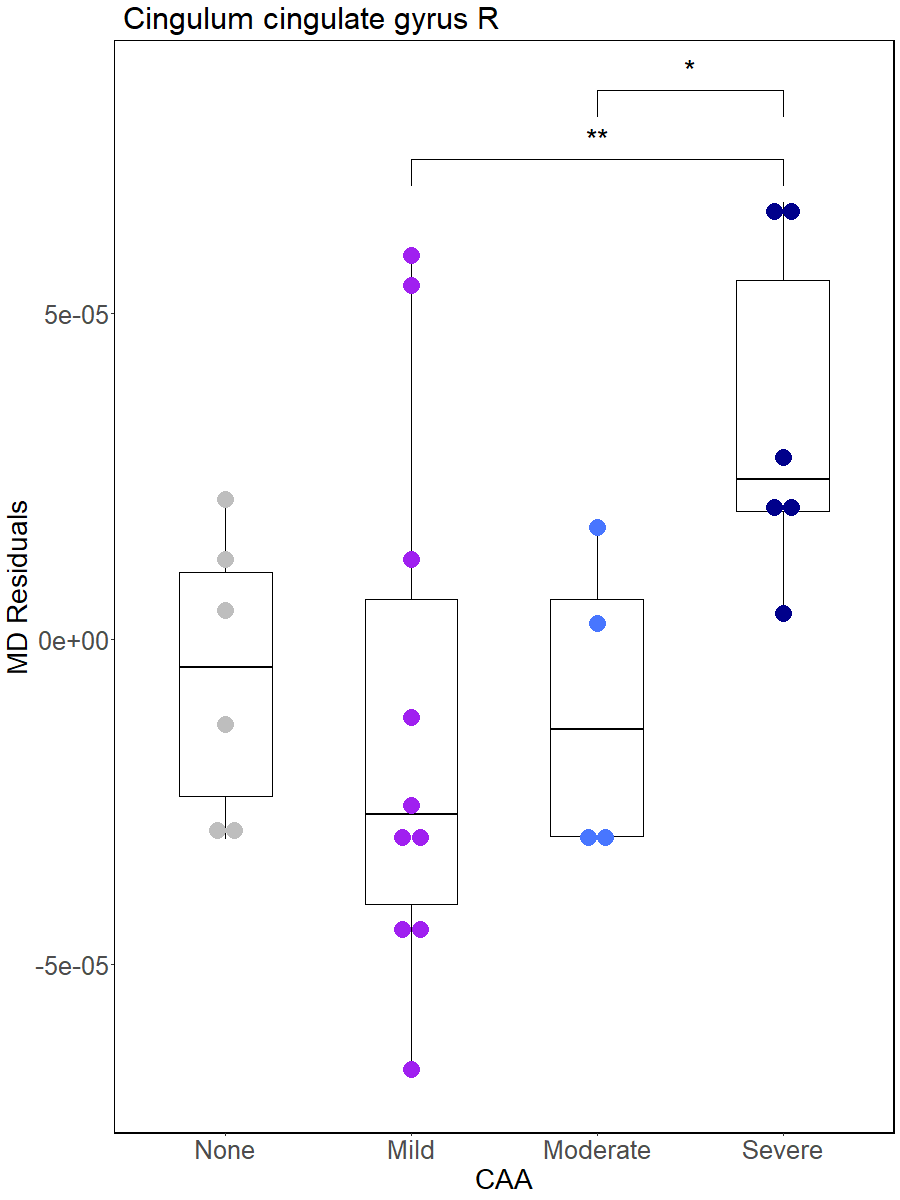

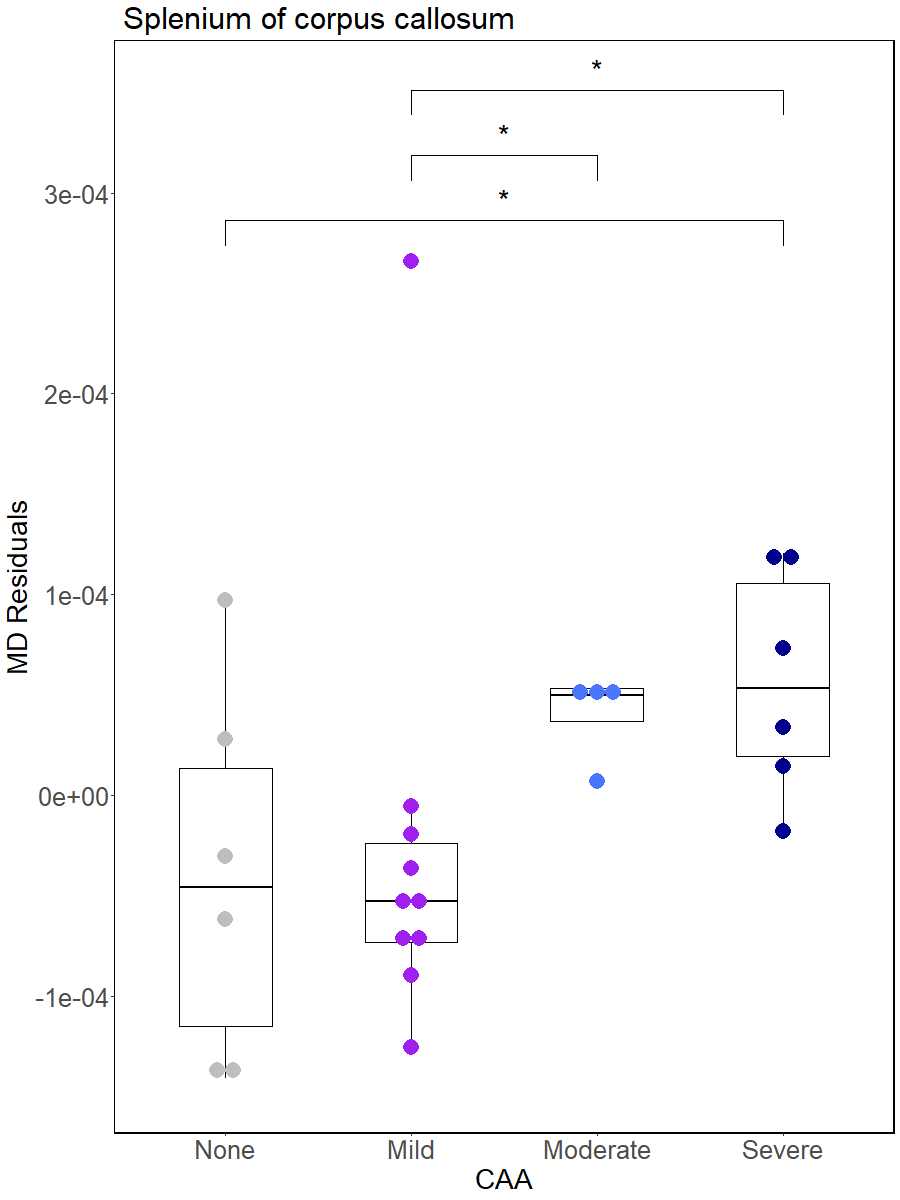


**
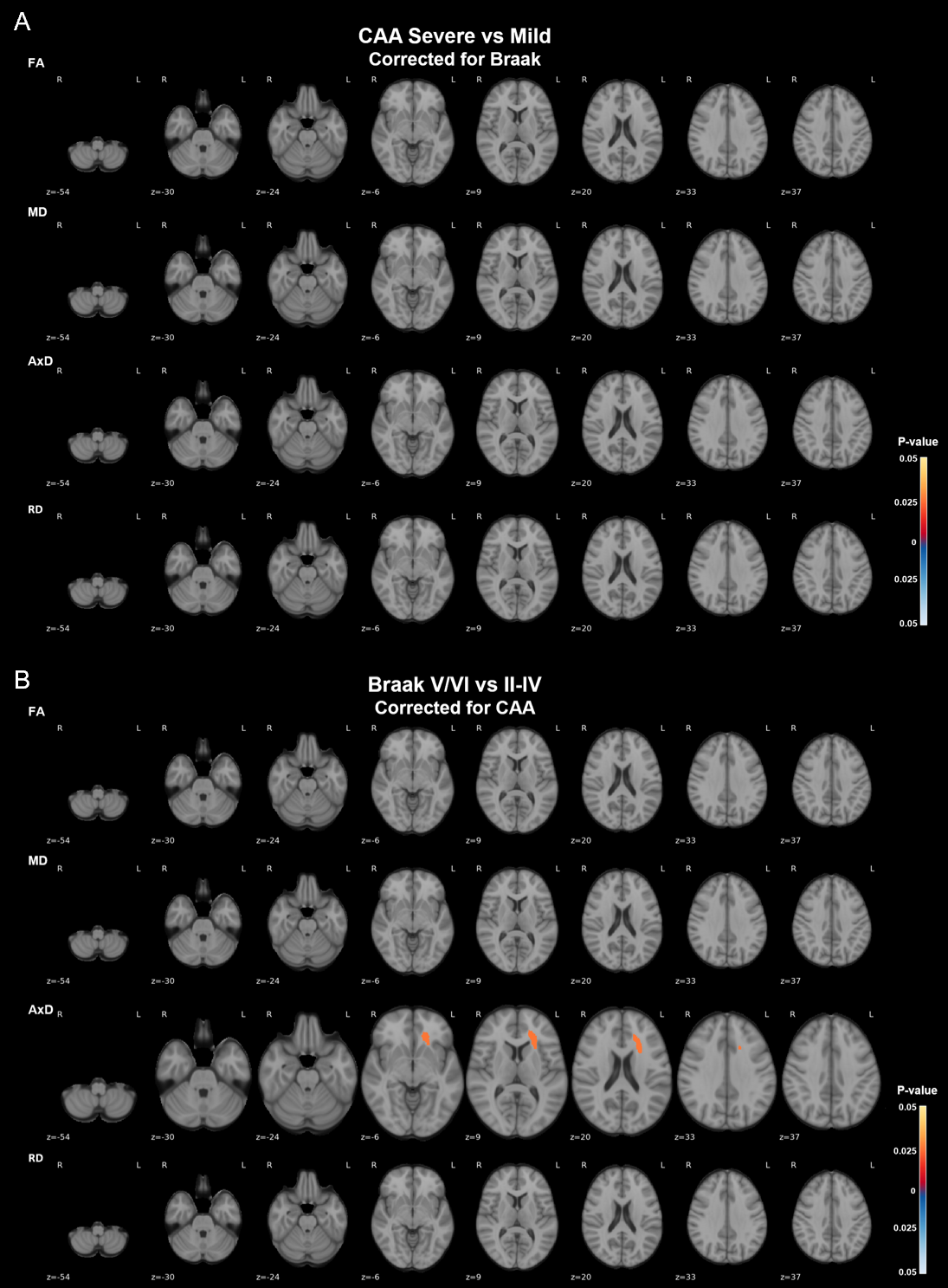
**

**Supplementary Figure 5 – Braak and CAA group regional white-matter DTI differences corrected for their co-pathology.** Supplementary figure 5 shows (A) the comparisons of DTI metrics linear model residuals for age, sex, b-values, MRI-to-death interval and Braak stage, between groups with mild and severe CAA. Only statistically significant comparisons (p < 0.05 with Kruskal-Wallis test followed by Dunn’s post-hoc test) are shown. (B) Differences in DTI metrics linear model residuals for age, sex, b-values, MRI-to-death interval and CAA severity, between Braak stages II-IV and V/VI. Only statistically significant comparisons (p < 0.05 with Wilcoxon test) are shown. The JHU-ICBM-DTI-48 white-matter atlas was used to extract regional DTI metrics for each subject, represented in axial views overlaid on the MNI 152 T1 template. The color bars show the p-values and the direction of the statistical differences, with reds for Severe CAA > Mild CAA (A) and Braak V/VI > Braak II-IV (B), and blues vice versa.

**Supplementary Tables**

**Supplementary Table 1 –** **MRI Scanner Specifications and DTI Acquisition Parameters.**

| **Subject** | **Center** |  | **Field Strength**  **(T)** | **Manufacter** | **Manufacter´s**  **Model name** | **b-value**  **(s/mm²)** | **Gradient Directions**  **(N)** | **b0**  **(N)** |
| --- | --- | --- | --- | --- | --- | --- | --- | --- |
| S01 | 1 |  | 3.0 | Siemens | Trio Tim | 1000 | 30 | 5 |
| S02 | 2 |  | 3.0 | Siemens | Trio Tim | 1000 | 80 | 12 |
| S03 | 3 |  | 3.0 | Siemens | Unknown | 1000 | 48 | 5 |
| S04 | 4 |  | 3.0 | GE | DISCOVERY MR 750 | 1000 | 30 | 2 |
| S05 | 4 |  | 1.5 | GE | Signa HDx | 1000 | 51 | 1 |
| S06 | 4 |  | 3.0 | GE | DISCOVERY MR 750 | 1000 | 30 | 2 |
| S07 | 4 |  | 3.0 | GE | DISCOVERY MR 750 | 1000 | 30 | 2 |
| S08 | 4 |  | 3.0 | GE | DISCOVERY MR 750 | 1000 | 30 | 2 |
| S09 | 5 |  | 3.0 | GE | DISCOVERY MR 750 | 1300 | 40 | 8 |
| S10 | 5 |  | 3.0 | GE | DISCOVERY MR 750 | 1300 | 40 | 8 |
| S11 | 5 |  | 3.0 | GE | DISCOVERY MR 750 | 1300 | 40 | 8 |
| S12 | 5 |  | 3.0 | GE | DISCOVERY MR 750 | 1300 | 40 | 8 |
| S13 | 5 |  | 3.0 | GE | DISCOVERY MR 750 | 1300 | 40 | 8 |
| S14 | 5 |  | 3.0 | GE | DISCOVERY MR 750 | 1300 | 40 | 8 |
| S15 | 5 |  | 3.0 | GE | DISCOVERY MR 750 | 1300 | 40 | 8 |
| S16 | 5 |  | 3.0 | GE | DISCOVERY MR 750 | 1300 | 40 | 8 |
| S17 | 5 |  | 3.0 | GE | DISCOVERY MR 750 | 1300 | 40 | 8 |
| S18 | 5 |  | 3.0 | GE | DISCOVERY MR 750 | 1300 | 40 | 8 |
| S19 | 5 |  | 3.0 | GE | DISCOVERY MR 750 | 1300 | 40 | 8 |
| S20 | 5 |  | 3.0 | GE | DISCOVERY MR 750 | 1300 | 40 | 8 |
| S21 | 5 |  | 3.0 | GE | DISCOVERY MR 750 | 1300 | 40 | 8 |
| S22 | 5 |  | 3.0 | GE | DISCOVERY MR 750 | 1300 | 40 | 8 |
| S23 | 5 |  | 3.0 | GE | DISCOVERY MR 750 | 1300 | 40 | 8 |
| S24 | 5 |  | 3.0 | GE | DISCOVERY MR 750 | 1300 | 40 | 8 |
| S25 | 5 |  | 3.0 | GE | DISCOVERY MR 750 | 1300 | 40 | 8 |
| S26 | 6 |  | 3.0 | GE | Unknown | 1000 | 25 | 1 |

**Supplementary Table** **2 –** **WM region-of-interest analyses demonstrated alterations in DTI metrics between different pathological group.**

|  |  | **CERAD**  **2 vs 3** | | | **Braak**  **II-IV vs V/VI** | | | **CAA** | | | | | | | | | | | | | |  |
| --- | --- | --- | --- | --- | --- | --- | --- | --- | --- | --- | --- | --- | --- | --- | --- | --- | --- | --- | --- | --- | --- | --- |
|  |  |  |  |  |  |  |  |  | | **0 vs 1** | | **0 vs 2** | | **0 vs 3** | | **1 vs 2** | | **1 vs 3** | | **2 vs 3** | | |
| **Region** | Scalar | | pval | adjp | | pval | adjp | | pval | pval | adjp | pval | adjp | pval | adjp | pval | adjp | pval | adjp | pval | adjp | |
| **ACR L** | FA | | 0.977 | 0.977 | | 0.874 | 0.956 | | 0.358 | - | - | - | - | - | - | - | - | - | - | - | - | |
|  | MD | | 0.752 | 1 | | 0.021 | 0.232 | | 0.18 | - | - | - | - | - | - | - | - | - | - | - | - | |
|  | AxD | | 0.437 | 0.849 | | 0.006 | 0.224 | | 0.193 | - | - | - | - | - | - | - | - | - | - | - | - | |
|  | RD | | 1 | 1 | | 0.085 | 0.269 | | 0.3 | - | - | - | - | - | - | - | - | - | - | - | - | |
| **ACR R** | FA | | 0.752 | 0.94 | | 0.396 | 0.956 | | 0.148 | - | - | - | - | - | - | - | - | - | - | - | - | |
|  | MD | | 1 | 1 | | 0.148 | 0.288 | | 0.399 | - | - | - | - | - | - | - | - | - | - | - | - | |
|  | AxD | | 0.841 | 0.977 | | 0.148 | 0.305 | | 0.473 | - | - | - | - | - | - | - | - | - | - | - | - | |
|  | RD | | 0.977 | 1 | | 0.181 | 0.353 | | 0.362 | - | - | - | - | - | - | - | - | - | - | - | - | |
| **ALIC L** | FA | | 0.472 | 0.919 | | 0.792 | 0.956 | | 0.95 | - | - | - | - | - | - | - | - | - | - | - | - | |
|  | MD | | 0.403 | 1 | | 0.792 | 0.792 | | 0.302 | - | - | - | - | - | - | - | - | - | - | - | - | |
|  | AxD | | 0.138 | 0.849 | | 0.287 | 0.457 | | 0.202 | - | - | - | - | - | - | - | - | - | - | - | - | |
|  | RD | | 0.625 | 1 | | 0.958 | 0.986 | | 0.488 | - | - | - | - | - | - | - | - | - | - | - | - | |
| **ALIC R** | FA | | 0.666 | 0.919 | | 0.597 | 0.956 | | 0.987 | - | - | - | - | - | - | - | - | - | - | - | - | |
|  | MD | | 0.546 | 1 | | 0.711 | 0.792 | | 0.602 | - | - | - | - | - | - | - | - | - | - | - | - | |
|  | AxD | | 0.472 | 0.868 | | 0.426 | 0.533 | | 0.488 | - | - | - | - | - | - | - | - | - | - | - | - | |
|  | RD | | 0.886 | 1 | | 0.56 | 0.633 | | 0.728 | - | - | - | - | - | - | - | - | - | - | - | - | |
| **BCC** | FA | | 0.259 | 0.919 | | 0.874 | 0.956 | | 0.486 | - | - | - | - | - | - | - | - | - | - | - | - | |
|  | MD | | 0.666 | 1 | | 0.2 | 0.318 | | 0.19 | - | - | - | - | - | - | - | - | - | - | - | - | |
|  | AxD | | 0.437 | 0.849 | | 0.133 | 0.305 | | 0.403 | - | - | - | - | - | - | - | - | - | - | - | - | |
|  | RD | | 0.709 | 1 | | 0.181 | 0.353 | | 0.162 | - | - | - | - | - | - | - | - | - | - | - | - | |
| **CCG L** | FA | | 0.931 | 0.977 | | 0.133 | 0.956 | | 0.156 | - | - | - | - | - | - | - | - | - | - | - | - | |
|  | MD | | 0.235 | 1 | | 0.339 | 0.456 | | 0.05 | - | - | - | - | - | - | - | - | - | - | - | - | |
|  | AxD | | 0.235 | 0.849 | | 0.958 | 0.986 | | 0.387 | - | - | - | - | - | - | - | - | - | - | - | - | |
|  | RD | | 0.709 | 1 | | 0.085 | 0.269 | | 0.045 | 0.826 | 0.826 | 0.661 | 0.991 | 0.774 | 0.929 | 0.014 | 0.042 | 0.012 | 0.07 | 0.079 | 0.158 | |
| **CCG R** | FA | | 0.138 | 0.919 | | 0.792 | 0.956 | | 0.669 | - | - | - | - | - | - | - | - | - | - | - | - | |
|  | MD | | 0.472 | 1 | | 0.525 | 0.613 | | 0.047 | 0.494 | 0.741 | 0.685 | 0.822 | 0.877 | 0.877 | 0.07 | 0.14 | 0.007 | 0.04 | 0.043 | 0.128 | |
|  | AxD | | 0.977 | 0.977 | | 0.874 | 0.927 | | 0.087 | - | - | - | - | - | - | - | - | - | - | - | - | |
|  | RD | | 0.403 | 1 | | 0.491 | 0.593 | | 0.219 | - | - | - | - | - | - | - | - | - | - | - | - | |
| **CH L** | FA | | 0.192 | 0.919 | | 0.396 | 0.956 | | 0.836 | - | - | - | - | - | - | - | - | - | - | - | - | |
|  | MD | | 0.709 | 1 | | 0.085 | 0.267 | | 0.215 | - | - | - | - | - | - | - | - | - | - | - | - | |
|  | AxD | | 0.886 | 0.977 | | 0.263 | 0.439 | | 0.286 | - | - | - | - | - | - | - | - | - | - | - | - | |
|  | RD | | 0.472 | 1 | | 0.058 | 0.269 | | 0.191 | - | - | - | - | - | - | - | - | - | - | - | - | |
| **CHR** | FA | | 0.546 | 0.919 | | 0.597 | 0.956 | | 0.407 | - | - | - | - | - | - | - | - | - | - | - | - | |
|  | MD | | 0.752 | 1 | | 0.107 | 0.267 | | 0.322 | - | - | - | - | - | - | - | - | - | - | - | - | |
|  | AxD | | 0.212 | 0.849 | | 0.107 | 0.305 | | 0.615 | - | - | - | - | - | - | - | - | - | - | - | - | |
|  | RD | | 0.796 | 1 | | 0.241 | 0.383 | | 0.172 | - | - | - | - | - | - | - | - | - | - | - | - | |
| **CT L** | FA | | 0.886 | 0.977 | | 0.525 | 0.956 | | 0.965 | - | - | - | - | - | - | - | - | - | - | - | - | |
|  | MD | | 1 | 1 | | 0.148 | 0.288 | | 0.786 | - | - | - | - | - | - | - | - | - | - | - | - | |
|  | AxD | | 0.752 | 0.977 | | 0.367 | 0.513 | | 0.905 | - | - | - | - | - | - | - | - | - | - | - | - | |
|  | RD | | 0.841 | 1 | | 0.058 | 0.269 | | 0.894 | - | - | - | - | - | - | - | - | - | - | - | - | |
| **CT R** | FA | | 0.285 | 0.919 | | 0.133 | 0.956 | | 0.982 | - | - | - | - | - | - | - | - | - | - | - | - | |
|  | MD | | 0.403 | 1 | | 0.751 | 0.792 | | 0.866 | - | - | - | - | - | - | - | - | - | - | - | - | |
|  | AxD | | 0.312 | 0.849 | | 1 | 1 | | 0.708 | - | - | - | - | - | - | - | - | - | - | - | - | |
|  | RD | | 0.508 | 1 | | 0.339 | 0.474 | | 0.915 | - | - | - | - | - | - | - | - | - | - | - | - | |
| **EC L** | FA | | 0.437 | 0.919 | | 1 | 1 | | 0.993 | - | - | - | - | - | - | - | - | - | - | - | - | |
|  | MD | | 0.585 | 1 | | 0.095 | 0.267 | | 0.957 | - | - | - | - | - | - | - | - | - | - | - | - | |
|  | AxD | | 0.285 | 0.849 | | 0.066 | 0.257 | | 0.933 | - | - | - | - | - | - | - | - | - | - | - | - | |
|  | RD | | 0.585 | 1 | | 0.181 | 0.353 | | 0.988 | - | - | - | - | - | - | - | - | - | - | - | - | |
| **EC R** | FA | | 0.437 | 0.919 | | 0.672 | 0.956 | | 0.704 | - | - | - | - | - | - | - | - | - | - | - | - | |
|  | MD | | 0.546 | 1 | | 0.525 | 0.613 | | 0.952 | - | - | - | - | - | - | - | - | - | - | - | - | |
|  | AxD | | 0.341 | 0.849 | | 0.396 | 0.513 | | 0.951 | - | - | - | - | - | - | - | - | - | - | - | - | |
|  | RD | | 0.886 | 1 | | 0.525 | 0.613 | | 0.822 | - | - | - | - | - | - | - | - | - | - | - | - | |
| **Fx L** | FA | | 0.666 | 0.919 | | 0.2 | 0.956 | | 0.183 | - | - | - | - | - | - | - | - | - | - | - | - | |
|  | MD | | 0.546 | 1 | | 0.066 | 0.232 | | 0.441 | - | - | - | - | - | - | - | - | - | - | - | - | |
|  | AxD | | 0.341 | 0.849 | | 0.133 | 0.305 | | 0.245 | - | - | - | - | - | - | - | - | - | - | - | - | |
|  | RD | | 0.625 | 1 | | 0.066 | 0.269 | | 0.365 | - | - | - | - | - | - | - | - | - | - | - | - | |
| **Fx R** | FA | | 0.709 | 0.919 | | 0.034 | 0.956 | | 0.056 | - | - | - | - | - | - | - | - | - | - | - | - | |
|  | MD | | 0.341 | 1 | | 0.2 | 0.318 | | 0.238 | - | - | - | - | - | - | - | - | - | - | - | - | |
|  | AxD | | 0.341 | 0.849 | | 0.525 | 0.593 | | 0.182 | - | - | - | - | - | - | - | - | - | - | - | - | |
|  | RD | | 0.371 | 1 | | 0.075 | 0.269 | | 0.215 | - | - | - | - | - | - | - | - | - | - | - | - | |
| **GCC** | FA | | 0.508 | 0.919 | | 0.874 | 0.956 | | 0.626 | - | - | - | - | - | - | - | - | - | - | - | - | |
|  | MD | | 0.709 | 1 | | 0.312 | 0.437 | | 0.267 | - | - | - | - | - | - | - | - | - | - | - | - | |
|  | AxD | | 0.709 | 0.977 | | 0.312 | 0.475 | | 0.102 | - | - | - | - | - | - | - | - | - | - | - | - | |
|  | RD | | 0.841 | 1 | | 0.426 | 0.533 | | 0.514 | - | - | - | - | - | - | - | - | - | - | - | - | |
| **PCR L** | FA | | 0.931 | 0.977 | | 0.634 | 0.956 | | 0.774 | - | - | - | - | - | - | - | - | - | - | - | - | |
|  | MD | | 0.752 | 1 | | 0.066 | 0.232 | | 0.242 | - | - | - | - | - | - | - | - | - | - | - | - | |
|  | AxD | | 0.796 | 0.977 | | 0.029 | 0.255 | | 0.323 | - | - | - | - | - | - | - | - | - | - | - | - | |
|  | RD | | 0.666 | 1 | | 0.12 | 0.299 | | 0.294 | - | - | - | - | - | - | - | - | - | - | - | - | |
| **PCR R** | FA | | 0.585 | 0.919 | | 0.751 | 0.956 | | 0.492 | - | - | - | - | - | - | - | - | - | - | - | - | |
|  | MD | | 0.977 | 1 | | 0.181 | 0.318 | | 0.2 | - | - | - | - | - | - | - | - | - | - | - | - | |
|  | AxD | | 0.977 | 0.977 | | 0.241 | 0.439 | | 0.146 | - | - | - | - | - | - | - | - | - | - | - | - | |
|  | RD | | 0.625 | 1 | | 0.367 | 0.475 | | 0.291 | - | - | - | - | - | - | - | - | - | - | - | - | |
| **PLIC L** | FA | | 0.312 | 0.919 | | 0.916 | 0.971 | | 0.629 | - | - | - | - | - | - | - | - | - | - | - | - | |
|  | MD | | 0.084 | 1 | | 0.058 | 0.232 | | 0.61 | - | - | - | - | - | - | - | - | - | - | - | - | |
|  | AxD | | 0.074 | 0.849 | | 0.039 | 0.255 | | 0.41 | - | - | - | - | - | - | - | - | - | - | - | - | |
|  | RD | | 0.403 | 1 | | 0.339 | 0.474 | | 0.832 | - | - | - | - | - | - | - | - | - | - | - | - | |
| **PLIC R** | FA | | 0.138 | 0.919 | | 0.874 | 0.956 | | 0.654 | - | - | - | - | - | - | - | - | - | - | - | - | |
|  | MD | | 0.341 | 1 | | 0.263 | 0.384 | | 0.461 | - | - | - | - | - | - | - | - | - | - | - | - | |
|  | AxD | | 0.096 | 0.849 | | 0.133 | 0.305 | | 0.337 | - | - | - | - | - | - | - | - | - | - | - | - | |
|  | RD | | 0.752 | 1 | | 0.916 | 0.971 | | 0.678 | - | - | - | - | - | - | - | - | - | - | - | - | |
| **PTR L** | FA | | 0.886 | 0.977 | | 0.396 | 0.956 | | 0.13 | - | - | - | - | - | - | - | - | - | - | - | - | |
|  | MD | | 0.886 | 1 | | 0.263 | 0.384 | | 0.134 | - | - | - | - | - | - | - | - | - | - | - | - | |
|  | AxD | | 0.977 | 0.977 | | 0.339 | 0.494 | | 0.304 | - | - | - | - | - | - | - | - | - | - | - | - | |
|  | RD | | 0.977 | 1 | | 0.22 | 0.383 | | 0.101 | - | - | - | - | - | - | - | - | - | - | - | - | |
| **PTR R** | FA | | 0.709 | 0.919 | | 0.525 | 0.956 | | 0.106 | - | - | - | - | - | - | - | - | - | - | - | - | |
|  | MD | | 0.931 | 1 | | 0.426 | 0.533 | | 0.2 | - | - | - | - | - | - | - | - | - | - | - | - | |
|  | AxD | | 0.796 | 0.977 | | 0.491 | 0.573 | | 0.246 | - | - | - | - | - | - | - | - | - | - | - | - | |
|  | RD | | 0.931 | 1 | | 0.339 | 0.474 | | 0.171 | - | - | - | - | - | - | - | - | - | - | - | - | |
| **RPIC L** | FA | | 0.212 | 0.919 | | 0.597 | 0.956 | | 0.71 | - | - | - | - | - | - | - | - | - | - | - | - | |
|  | MD | | 1 | 1 | | 0.107 | 0.267 | | 0.166 | - | - | - | - | - | - | - | - | - | - | - | - | |
|  | AxD | | 0.437 | 0.849 | | 0.095 | 0.303 | | 0.403 | - | - | - | - | - | - | - | - | - | - | - | - | |
|  | RD | | 0.403 | 1 | | 0.22 | 0.383 | | 0.237 | - | - | - | - | - | - | - | - | - | - | - | - | |
| **RPIC R** | FA | | 0.508 | 0.919 | | 0.312 | 0.956 | | 0.642 | - | - | - | - | - | - | - | - | - | - | - | - | |
|  | MD | | 0.508 | 1 | | 0.133 | 0.288 | | 0.324 | - | - | - | - | - | - | - | - | - | - | - | - | |
|  | AxD | | 0.371 | 0.849 | | 0.396 | 0.513 | | 0.539 | - | - | - | - | - | - | - | - | - | - | - | - | |
|  | RD | | 0.977 | 1 | | 0.12 | 0.299 | | 0.334 | - | - | - | - | - | - | - | - | - | - | - | - | |
| **SS L** | FA | | 0.625 | 0.919 | | 0.181 | 0.956 | | 0.21 | - | - | - | - | - | - | - | - | - | - | - | - | |
|  | MD | | 0.796 | 1 | | 0.029 | 0.232 | | 0.33 | - | - | - | - | - | - | - | - | - | - | - | - | |
|  | AxD | | 0.508 | 0.868 | | 0.051 | 0.255 | | 0.652 | - | - | - | - | - | - | - | - | - | - | - | - | |
|  | RD | | 0.931 | 1 | | 0.029 | 0.269 | | 0.2 | - | - | - | - | - | - | - | - | - | - | - | - | |
| **SS R** | FA | | 0.709 | 0.919 | | 0.312 | 0.956 | | 0.225 | - | - | - | - | - | - | - | - | - | - | - | - | |
|  | MD | | 0.977 | 1 | | 0.12 | 0.279 | | 0.027 | 0.265 | 0.398 | 0.448 | 0.537 | 0.072 | 0.215 | 0.113 | 0.226 | 0.004 | 0.023 | 0.51 | 0.51 | |
|  | AxD | | 0.931 | 0.977 | | 0.263 | 0.439 | | 0.103 | - | - | - | - | - | - | - | - | - | - | - | - | |
|  | RD | | 0.977 | 1 | | 0.058 | 0.269 | | 0.021 | 0.227 | 0.341 | 0.468 | 0.562 | 0.065 | 0.195 | 0.113 | 0.226 | 0.003 | 0.017 | 0.489 | 0.489 | |
| **SCC** | FA | | 0.285 | 0.919 | | 0.458 | 0.956 | | 0.636 | - | - | - | - | - | - | - | - | - | - | - | - | |
|  | MD | | 0.841 | 1 | | 0.066 | 0.232 | | 0.024 | 0.906 | 0.906 | 0.082 | 0.123 | 0.045 | 0.091 | 0.031 | 0.094 | 0.012 | 0.07 | 0.853 | 1 | |
|  | AxD | | 0.546 | 0.868 | | 0.045 | 0.255 | | 0.184 | - | - | - | - | - | - | - | - | - | - | - | - | |
|  | RD | | 0.709 | 1 | | 0.133 | 0.311 | | 0.041 | 0.774 | 0.929 | 0.133 | 0.2 | 0.059 | 0.118 | 0.054 | 0.163 | 0.015 | 0.088 | 0.826 | 0.826 | |
| **SCR L** | FA | | 0.977 | 0.977 | | 0.792 | 0.956 | | 0.695 | - | - | - | - | - | - | - | - | - | - | - | - | |
|  | MD | | 0.796 | 1 | | 0.025 | 0.232 | | 0.313 | - | - | - | - | - | - | - | - | - | - | - | - | |
|  | AxD | | 0.437 | 0.849 | | 0.039 | 0.255 | | 0.404 | - | - | - | - | - | - | - | - | - | - | - | - | |
|  | RD | | 0.977 | 1 | | 0.045 | 0.269 | | 0.365 | - | - | - | - | - | - | - | - | - | - | - | - | |
| **SCR R** | FA | | 0.625 | 0.919 | | 0.711 | 0.956 | | 0.911 | - | - | - | - | - | - | - | - | - | - | - | - | |
|  | MD | | 0.886 | 1 | | 0.164 | 0.302 | | 0.152 | - | - | - | - | - | - | - | - | - | - | - | - | |
|  | AxD | | 0.709 | 0.977 | | 0.148 | 0.305 | | 0.214 | - | - | - | - | - | - | - | - | - | - | - | - | |
|  | RD | | 0.886 | 1 | | 0.241 | 0.383 | | 0.174 | - | - | - | - | - | - | - | - | - | - | - | - | |
| **SFOF L** | FA | | 0.931 | 0.977 | | 0.426 | 0.956 | | 0.132 | - | - | - | - | - | - | - | - | - | - | - | - | |
|  | MD | | 0.031 | 1 | | 0.051 | 0.232 | | 0.481 | - | - | - | - | - | - | - | - | - | - | - | - | |
|  | AxD | | 0.048 | 0.849 | | 0.085 | 0.296 | | 0.31 | - | - | - | - | - | - | - | - | - | - | - | - | |
|  | RD | | 0.108 | 1 | | 0.095 | 0.278 | | 0.465 | - | - | - | - | - | - | - | - | - | - | - | - | |
| **SFOF R** | FA | | 0.508 | 0.919 | | 0.874 | 0.956 | | 0.557 | - | - | - | - | - | - | - | - | - | - | - | - | |
|  | MD | | 0.371 | 1 | | 0.751 | 0.792 | | 0.286 | - | - | - | - | - | - | - | - | - | - | - | - | |
|  | AxD | | 0.212 | 0.849 | | 0.672 | 0.735 | | 0.222 | - | - | - | - | - | - | - | - | - | - | - | - | |
|  | RD | | 0.546 | 1 | | 0.672 | 0.735 | | 0.441 | - | - | - | - | - | - | - | - | - | - | - | - | |
| **SLF L** | FA | | 0.259 | 0.919 | | 0.263 | 0.956 | | 0.163 | - | - | - | - | - | - | - | - | - | - | - | - | |
|  | MD | | 0.437 | 1 | | 0.426 | 0.533 | | 0.331 | - | - | - | - | - | - | - | - | - | - | - | - | |
|  | AxD | | 0.796 | 0.977 | | 0.22 | 0.427 | | 0.491 | - | - | - | - | - | - | - | - | - | - | - | - | |
|  | RD | | 0.371 | 1 | | 0.367 | 0.475 | | 0.278 | - | - | - | - | - | - | - | - | - | - | - | - | |
| **SLF R** | FA | | 0.259 | 0.919 | | 1 | 1 | | 0.536 | - | - | - | - | - | - | - | - | - | - | - | - | |
|  | MD | | 0.285 | 1 | | 0.792 | 0.792 | | 0.094 | - | - | - | - | - | - | - | - | - | - | - | - | |
|  | AxD | | 0.546 | 0.868 | | 0.458 | 0.553 | | 0.079 | - | - | - | - | - | - | - | - | - | - | - | - | |
|  | RD | | 0.285 | 1 | | 1 | 1 | | 0.133 | - | - | - | - | - | - | - | - | - | - | - | - | |
| **Tp L** | FA | | 0.472 | 0.919 | | 0.792 | 0.956 | | 0.604 | - | - | - | - | - | - | - | - | - | - | - | - | |
|  | MD | | 0.709 | 1 | | 0.058 | 0.232 | | 0.348 | - | - | - | - | - | - | - | - | - | - | - | - | |
|  | AxD | | 0.841 | 0.977 | | 0.066 | 0.257 | | 0.379 | - | - | - | - | - | - | - | - | - | - | - | - | |
|  | RD | | 0.625 | 1 | | 0.051 | 0.269 | | 0.39 | - | - | - | - | - | - | - | - | - | - | - | - | |
| **Tp R** | FA | | 0.403 | 0.919 | | 0.367 | 0.956 | | 0.931 | - | - | - | - | - | - | - | - | - | - | - | - | |
|  | MD | | 0.212 | 1 | | 0.025 | 0.232 | | 0.065 | - | - | - | - | - | - | - | - | - | - | - | - | |
|  | AxD | | 0.312 | 0.849 | | 0.029 | 0.255 | | 0.11 | - | - | - | - | - | - | - | - | - | - | - | - | |
|  | RD | | 0.154 | 1 | | 0.011 | 0.269 | | 0.072 | - | - | - | - | - | - | - | - | - | - | - | - | |

Statistical analyses of regional DTI metrics linear model residuals for age, sex, MRI-to-death interval and b-values were performed using the Wilcoxon test for the CERAD scores 2 and 3 and for Braak pathology stages II-IV and V/VI, while the Kruskal-Wallis test followed by Dunn's post-hoc test for pairwise comparisons was applied to the CAA groups. To account for multiple comparisons, Benjamini-Hochberg false discovery rate corrections were applied.The JHU-ICBM-DTI-48 WM atlas was used to extract regional DTI metrics for each subject. ROIs abbreviations: ACR - Anterior Corona Radiata, ALIC - Anterior limb of internal capsule, BCC - Body of corpus callosum, CCG - Cingulum cingulate gyrus, CH - Cingulum hippocampus, CT - Corticospinal tract, EC – External capsule, Fx - Fornix, GCC - Genu of corpus callosum, PCR - Posterior corona radiata, PLIC - Posterior limb of internal capsule, PTR - Posterior thalamic radiation, RPIC - Retrolenticular part of internal capsule, SS - Sagittal stratum, SCC - Splenium of corpus callosum, SCR - Superior corona radiata, SFOF - Superior fronto occipital fasciculus, SLF - Superior longitudinal fasciculus, Tp - Tapetum.

**Supplementary Table 3 – WM region-of-interest BRAAK group analyses corrected for the CAA pathology, and vice-versa.**

|  |  | **Braak**  **II-IV vs V/VI** | | **CAA** | | | | | | | | | | | | |
| --- | --- | --- | --- | --- | --- | --- | --- | --- | --- | --- | --- | --- | --- | --- | --- | --- |
|  |  |  |  |  | 0 vs 1 | | 0 vs 2 | | 0 vs 3 | | 1 vs 2 | | 1 vs 3 | | 2 vs 3 | |
| **Region** | Scalar | pval | adjp | pval | pval | adjp | pval | adjp | pval | adjp | pval | adjp | pval | adjp | pval | adjp |
| **ACR L** | FA | 0.916 | 1 | 0.461 | - | - | - | - | - | - | - | - | - | - | - | - |
|  | MD | 0.051 | 0.649 | 0.951 | - | - | - | - | - | - | - | - | - | - | - | - |
|  | AxD | 0.025 | 0.623 | 0.771 | - | - | - | - | - | - | - | - | - | - | - | - |
|  | RD | 0.181 | 0.641 | 0.969 | - | - | - | - | - | - | - | - | - | - | - | - |
| **ACR R** | FA | 0.367 | 1 | 0.21 | - | - | - | - | - | - | - | - | - | - | - | - |
|  | MD | 0.396 | 0.66 | 0.734 | - | - | - | - | - | - | - | - | - | - | - | - |
|  | AxD | 0.525 | 0.875 | 0.531 | - | - | - | - | - | - | - | - | - | - | - | - |
|  | RD | 0.312 | 0.668 | 0.814 | - | - | - | - | - | - | - | - | - | - | - | - |
| **ALIC L** | FA | 0.874 | 1 | 0.978 | - | - | - | - | - | - | - | - | - | - | - | - |
|  | MD | 1 | 1 | 0.25 | - | - | - | - | - | - | - | - | - | - | - | - |
|  | AxD | 0.634 | 0.888 | 0.105 | - | - | - | - | - | - | - | - | - | - | - | - |
|  | RD | 0.916 | 0.916 | 0.525 | - | - | - | - | - | - | - | - | - | - | - | - |
| **ALIC R** | FA | 0.458 | 1 | 0.999 | - | - | - | - | - | - | - | - | - | - | - | - |
|  | MD | 0.958 | 0.986 | 0.614 | - | - | - | - | - | - | - | - | - | - | - | - |
|  | AxD | 0.672 | 0.889 | 0.443 | - | - | - | - | - | - | - | - | - | - | - | - |
|  | RD | 0.711 | 0.754 | 0.473 | - | - | - | - | - | - | - | - | - | - | - | - |
| **BCC** | FA | 1 | 1 | 0.481 | - | - | - | - | - | - | - | - | - | - | - | - |
|  | MD | 0.367 | 0.66 | 0.294 | - | - | - | - | - | - | - | - | - | - | - | - |
|  | AxD | 0.263 | 0.67 | 0.232 | - | - | - | - | - | - | - | - | - | - | - | - |
|  | RD | 0.458 | 0.668 | 0.2 | - | - | - | - | - | - | - | - | - | - | - | - |
| **CCG L** | FA | 0.263 | 1 | 0.64 | - | - | - | - | - | - | - | - | - | - | - | - |
|  | MD | 0.339 | 0.66 | 0.155 | - | - | - | - | - | - | - | - | - | - | - | - |
|  | AxD | 0.711 | 0.889 | 0.413 | - | - | - | - | - | - | - | - | - | - | - | - |
|  | RD | 0.181 | 0.641 | 0.153 | - | - | - | - | - | - | - | - | - | - | - | - |
| **CCG R** | FA | 0.751 | 1 | 0.766 | - | - | - | - | - | - | - | - | - | - | - | - |
|  | MD | 0.672 | 0.811 | 0.137 | - | - | - | - | - | - | - | - | - | - | - | - |
|  | AxD | 0.874 | 0.956 | 0.1 | - | - | - | - | - | - | - | - | - | - | - | - |
|  | RD | 0.56 | 0.754 | 0.261 | - | - | - | - | - | - | - | - | - | - | - | - |
| **CH L** | FA | 0.426 | 1 | 0.861 | - | - | - | - | - | - | - | - | - | - | - | - |
|  | MD | 0.367 | 0.66 | 0.224 | - | - | - | - | - | - | - | - | - | - | - | - |
|  | AxD | 0.525 | 0.875 | 0.289 | - | - | - | - | - | - | - | - | - | - | - | - |
|  | RD | 0.263 | 0.668 | 0.194 | - | - | - | - | - | - | - | - | - | - | - | - |
| **CHR** | FA | 0.711 | 1 | 0.48 | - | - | - | - | - | - | - | - | - | - | - | - |
|  | MD | 0.426 | 0.668 | 0.388 | - | - | - | - | - | - | - | - | - | - | - | - |
|  | AxD | 0.2 | 0.641 | 0.946 | - | - | - | - | - | - | - | - | - | - | - | - |
|  | RD | 0.597 | 0.754 | 0.152 | - | - | - | - | - | - | - | - | - | - | - | - |
| **CT L** | FA | 0.458 | 1 | 0.692 | - | - | - | - | - | - | - | - | - | - | - | - |
|  | MD | 0.2 | 0.649 | 0.707 | - | - | - | - | - | - | - | - | - | - | - | - |
|  | AxD | 0.525 | 0.875 | 0.86 | - | - | - | - | - | - | - | - | - | - | - | - |
|  | RD | 0.107 | 0.641 | 0.789 | - | - | - | - | - | - | - | - | - | - | - | - |
| **CT R** | FA | 0.12 | 1 | 0.673 | - | - | - | - | - | - | - | - | - | - | - | - |
|  | MD | 0.958 | 0.986 | 0.604 | - | - | - | - | - | - | - | - | - | - | - | - |
|  | AxD | 0.711 | 0.889 | 0.535 | - | - | - | - | - | - | - | - | - | - | - | - |
|  | RD | 0.426 | 0.668 | 0.564 | - | - | - | - | - | - | - | - | - | - | - | - |
| **EC L** | FA | 1 | 1 | 0.991 | - | - | - | - | - | - | - | - | - | - | - | - |
|  | MD | 0.287 | 0.66 | 0.729 | - | - | - | - | - | - | - | - | - | - | - | - |
|  | AxD | 0.133 | 0.641 | 0.711 | - | - | - | - | - | - | - | - | - | - | - | - |
|  | RD | 0.396 | 0.668 | 0.77 | - | - | - | - | - | - | - | - | - | - | - | - |
| **EC R** | FA | 0.634 | 1 | 0.952 | - | - | - | - | - | - | - | - | - | - | - | - |
|  | MD | 0.597 | 0.746 | 0.915 | - | - | - | - | - | - | - | - | - | - | - | - |
|  | AxD | 0.597 | 0.888 | 0.853 | - | - | - | - | - | - | - | - | - | - | - | - |
|  | RD | 0.634 | 0.754 | 0.941 | - | - | - | - | - | - | - | - | - | - | - | - |
| **Fx L** | FA | 0.458 | 1 | 0.372 | - | - | - | - | - | - | - | - | - | - | - | - |
|  | MD | 0.241 | 0.649 | 0.299 | - | - | - | - | - | - | - | - | - | - | - | - |
|  | AxD | 0.339 | 0.741 | 0.198 | - | - | - | - | - | - | - | - | - | - | - | - |
|  | RD | 0.22 | 0.641 | 0.484 | - | - | - | - | - | - | - | - | - | - | - | - |
| **Fx R** | FA | 0.085 | 1 | 0.168 | - | - | - | - | - | - | - | - | - | - | - | - |
|  | MD | 0.312 | 0.66 | 0.264 | - | - | - | - | - | - | - | - | - | - | - | - |
|  | AxD | 0.916 | 0.958 | 0.304 | - | - | - | - | - | - | - | - | - | - | - | - |
|  | RD | 0.133 | 0.641 | 0.375 | - | - | - | - | - | - | - | - | - | - | - | - |
| **GCC** | FA | 0.751 | 1 | 0.893 | - | - | - | - | - | - | - | - | - | - | - | - |
|  | MD | 0.525 | 0.707 | 0.217 | - | - | - | - | - | - | - | - | - | - | - | - |
|  | AxD | 0.426 | 0.875 | 0.043 | 0.505 | 0.757 | 0.045 | 0.089 | 0.006 | 0.033 | 0.734 | 0.881 | 0.774 | 0.774 | 0.021 | 0.062 |
|  | RD | 0.634 | 0.754 | 0.383 | - | - | - | - | - | - | - | - | - | - | - | - |
| **PCR L** | FA | 0.751 | 1 | 0.764 | - | - | - | - | - | - | - | - | - | - | - | - |
|  | MD | 0.12 | 0.649 | 0.883 | - | - | - | - | - | - | - | - | - | - | - | - |
|  | AxD | 0.085 | 0.623 | 0.875 | - | - | - | - | - | - | - | - | - | - | - | - |
|  | RD | 0.181 | 0.641 | 0.766 | - | - | - | - | - | - | - | - | - | - | - | - |
| **PCR R** | FA | 0.711 | 1 | 0.482 | - | - | - | - | - | - | - | - | - | - | - | - |
|  | MD | 0.458 | 0.668 | 0.393 | - | - | - | - | - | - | - | - | - | - | - | - |
|  | AxD | 0.634 | 0.888 | 0.353 | - | - | - | - | - | - | - | - | - | - | - | - |
|  | RD | 0.458 | 0.668 | 0.502 | - | - | - | - | - | - | - | - | - | - | - | - |
| **PLIC L** | FA | 0.916 | 1 | 0.685 | - | - | - | - | - | - | - | - | - | - | - | - |
|  | MD | 0.263 | 0.658 | 0.741 | - | - | - | - | - | - | - | - | - | - | - | - |
|  | AxD | 0.085 | 0.623 | 0.221 | - | - | - | - | - | - | - | - | - | - | - | - |
|  | RD | 0.287 | 0.668 | 0.653 | - | - | - | - | - | - | - | - | - | - | - | - |
| **PLIC R** | FA | 0.916 | 1 | 0.702 | - | - | - | - | - | - | - | - | - | - | - | - |
|  | MD | 0.56 | 0.726 | 0.887 | - | - | - | - | - | - | - | - | - | - | - | - |
|  | AxD | 0.22 | 0.641 | 0.348 | - | - | - | - | - | - | - | - | - | - | - | - |
|  | RD | 0.711 | 0.754 | 0.767 | - | - | - | - | - | - | - | - | - | - | - | - |
| **PTR L** | FA | 0.339 | 1 | 0.262 | - | - | - | - | - | - | - | - | - | - | - | - |
|  | MD | 0.458 | 0.668 | 0.403 | - | - | - | - | - | - | - | - | - | - | - | - |
|  | AxD | 0.792 | 0.924 | 0.611 | - | - | - | - | - | - | - | - | - | - | - | - |
|  | RD | 0.426 | 0.668 | 0.569 | - | - | - | - | - | - | - | - | - | - | - | - |
| **PTR R** | FA | 0.458 | 1 | 0.192 | - | - | - | - | - | - | - | - | - | - | - | - |
|  | MD | 0.751 | 0.848 | 0.563 | - | - | - | - | - | - | - | - | - | - | - | - |
|  | AxD | 0.958 | 0.958 | 0.475 | - | - | - | - | - | - | - | - | - | - | - | - |
|  | RD | 0.672 | 0.754 | 0.362 | - | - | - | - | - | - | - | - | - | - | - | - |
| **RPIC L** | FA | 0.672 | 1 | 0.898 | - | - | - | - | - | - | - | - | - | - | - | - |
|  | MD | 0.241 | 0.649 | 0.477 | - | - | - | - | - | - | - | - | - | - | - | - |
|  | AxD | 0.287 | 0.67 | 0.538 | - | - | - | - | - | - | - | - | - | - | - | - |
|  | RD | 0.458 | 0.668 | 0.644 | - | - | - | - | - | - | - | - | - | - | - | - |
| **RPIC R** | FA | 0.339 | 1 | 0.643 | - | - | - | - | - | - | - | - | - | - | - | - |
|  | MD | 0.22 | 0.649 | 0.74 | - | - | - | - | - | - | - | - | - | - | - | - |
|  | AxD | 0.634 | 0.888 | 0.675 | - | - | - | - | - | - | - | - | - | - | - | - |
|  | RD | 0.181 | 0.641 | 0.913 | - | - | - | - | - | - | - | - | - | - | - | - |
| **SS L** | FA | 0.312 | 1 | 0.767 | - | - | - | - | - | - | - | - | - | - | - | - |
|  | MD | 0.164 | 0.649 | 0.584 | - | - | - | - | - | - | - | - | - | - | - | - |
|  | AxD | 0.287 | 0.67 | 0.858 | - | - | - | - | - | - | - | - | - | - | - | - |
|  | RD | 0.148 | 0.641 | 0.351 | - | - | - | - | - | - | - | - | - | - | - | - |
| **SS R** | FA | 0.312 | 1 | 0.348 | - | - | - | - | - | - | - | - | - | - | - | - |
|  | MD | 0.396 | 0.66 | 0.134 | - | - | - | - | - | - | - | - | - | - | - | - |
|  | AxD | 0.958 | 0.958 | 0.314 | - | - | - | - | - | - | - | - | - | - | - | - |
|  | RD | 0.396 | 0.668 | 0.085 | - | - | - | - | - | - | - | - | - | - | - | - |
| **SCC** | FA | 0.711 | 1 | 0.937 | - | - | - | - | - | - | - | - | - | - | - | - |
|  | MD | 0.133 | 0.649 | 0.134 | - | - | - | - | - | - | - | - | - | - | - | - |
|  | AxD | 0.22 | 0.641 | 0.142 | - | - | - | - | - | - | - | - | - | - | - | - |
|  | RD | 0.312 | 0.668 | 0.363 | - | - | - | - | - | - | - | - | - | - | - | - |
| **SCR L** | FA | 0.792 | 1 | 0.659 | - | - | - | - | - | - | - | - | - | - | - | - |
|  | MD | 0.085 | 0.649 | 0.933 | - | - | - | - | - | - | - | - | - | - | - | - |
|  | AxD | 0.075 | 0.623 | 0.868 | - | - | - | - | - | - | - | - | - | - | - | - |
|  | RD | 0.2 | 0.641 | 0.865 | - | - | - | - | - | - | - | - | - | - | - | - |
| **SCR R** | FA | 0.916 | 1 | 0.886 | - | - | - | - | - | - | - | - | - | - | - | - |
|  | MD | 0.241 | 0.649 | 0.523 | - | - | - | - | - | - | - | - | - | - | - | - |
|  | AxD | 0.22 | 0.641 | 0.492 | - | - | - | - | - | - | - | - | - | - | - | - |
|  | RD | 0.426 | 0.668 | 0.585 | - | - | - | - | - | - | - | - | - | - | - | - |
| **SFOF L** | FA | 0.396 | 1 | 0.216 | - | - | - | - | - | - | - | - | - | - | - | - |
|  | MD | 0.181 | 0.649 | 0.095 | - | - | - | - | - | - | - | - | - | - | - | - |
|  | AxD | 0.22 | 0.641 | 0.082 | - | - | - | - | - | - | - | - | - | - | - | - |
|  | RD | 0.22 | 0.641 | 0.18 | - | - | - | - | - | - | - | - | - | - | - | - |
| **SFOF R** | FA | 0.874 | 1 | 0.486 | - | - | - | - | - | - | - | - | - | - | - | - |
|  | MD | 0.751 | 0.848 | 0.302 | - | - | - | - | - | - | - | - | - | - | - | - |
|  | AxD | 0.792 | 0.924 | 0.194 | - | - | - | - | - | - | - | - | - | - | - | - |
|  | RD | 0.672 | 0.754 | 0.368 | - | - | - | - | - | - | - | - | - | - | - | - |
| **SLF L** | FA | 0.491 | 1 | 0.557 | - | - | - | - | - | - | - | - | - | - | - | - |
|  | MD | 0.491 | 0.687 | 0.713 | - | - | - | - | - | - | - | - | - | - | - | - |
|  | AxD | 0.525 | 0.875 | 0.634 | - | - | - | - | - | - | - | - | - | - | - | - |
|  | RD | 0.597 | 0.754 | 0.55 | - | - | - | - | - | - | - | - | - | - | - | - |
| **SLF R** | FA | 1 | 1 | 0.552 | - | - | - | - | - | - | - | - | - | - | - | - |
|  | MD | 0.833 | 0.911 | 0.203 | - | - | - | - | - | - | - | - | - | - | - | - |
|  | AxD | 0.874 | 0.956 | 0.266 | - | - | - | - | - | - | - | - | - | - | - | - |
|  | RD | 0.916 | 0.916 | 0.258 | - | - | - | - | - | - | - | - | - | - | - | - |
| **Tp L** | FA | 0.525 | 1 | 0.565 | - | - | - | - | - | - | - | - | - | - | - | - |
|  | MD | 0.095 | 0.649 | 0.183 | - | - | - | - | - | - | - | - | - | - | - | - |
|  | AxD | 0.107 | 0.623 | 0.294 | - | - | - | - | - | - | - | - | - | - | - | - |
|  | RD | 0.095 | 0.641 | 0.146 | - | - | - | - | - | - | - | - | - | - | - | - |
| **Tp R** | FA | 0.367 | 1 | 0.874 | - | - | - | - | - | - | - | - | - | - | - | - |
|  | MD | 0.075 | 0.649 | 0.304 | - | - | - | - | - | - | - | - | - | - | - | - |
|  | AxD | 0.095 | 0.623 | 0.383 | - | - | - | - | - | - | - | - | - | - | - | - |
|  | RD | 0.095 | 0.641 | 0.371 | - | - | - | - | - | - | - | - | - | - | - | - |

Statistical analyses of regional DTI metrics linear model residuals for age, sex, MRI-to-death interval, b-values and Braak stage (for CAA group comparisons) or CAA severity (for Braak group comparisons) were performed using the Wilcoxon test between Braak stages II-IV and V/VI, and the Kruskal-Wallis test followed by Dunn's post-hoc test for the CAA groups. To account for multiple comparisons, Benjamini-Hochberg false discovery rate corrections were applied. The JHU-ICBM-DTI-48 WM atlas was used to extract regional DTI metrics for each subject. ROIs abbreviations: ACR - Anterior Corona Radiata, ALIC - Anterior limb of internal capsule, BCC - Body of corpus callosum, CCG - Cingulum cingulate gyrus, CH - Cingulum hippocampus, CT - Corticospinal tract, EC – External capsule, Fx - Fornix, GCC - Genu of corpus callosum, PCR - Posterior corona radiata, PLIC - Posterior limb of internal capsule, PTR - Posterior thalamic radiation, RPIC - Retrolenticular part of internal capsule, SS - Sagittal stratum, SCC - Splenium of corpus callosum, SCR - Superior corona radiata, SFOF - Superior fronto occipital fasciculus, SLF - Superior longitudinal fasciculus, Tp - Tapetum

**Supplementary Table 4 – Correlations between regional DTI scalars and CDR scores.**

| **Region** | **Scalar** | **ρ** | **pval** | **adjpval** |
| --- | --- | --- | --- | --- |
| **Anterior corona radiata L** | FA | 0.100 | 0.628 | 0.84 |
|  | MD | 0.437 | 0.026 | 0.161 |
|  | AxD | 0.455 | 0.02 | 0.086 |
|  | RD | 0.375 | 0.059 | 0.233 |
| **Anterior corona radiata R** | FA | 0.126 | 0.538 | 0.812 |
|  | MD | 0.354 | 0.076 | 0.243 |
|  | AxD | 0.417 | 0.034 | 0.106 |
|  | RD | 0.303 | 0.132 | 0.332 |
| **Anterior limb of internal capsule L** | FA | -0.009 | 0.964 | 0.988 |
|  | MD | 0.211 | 0.3 | 0.353 |
|  | AxD | 0.267 | 0.187 | 0.281 |
|  | RD | 0.173 | 0.399 | 0.537 |
| **Anterior limb of internal capsule R** | FA | -0.014 | 0.946 | 0.988 |
|  | MD | 0.210 | 0.302 | 0.353 |
|  | AxD | 0.273 | 0.176 | 0.281 |
|  | RD | 0.228 | 0.262 | 0.483 |
| **Body of corpus callosum** | FA | -0.294 | 0.144 | 0.721 |
|  | MD | 0.464 | 0.017 | 0.161 |
|  | AxD | 0.412 | 0.036 | 0.106 |
|  | RD | 0.443 | 0.024 | 0.233 |
| **Cingulum cingulate gyrus L** | FA | -0.223 | 0.273 | 0.812 |
|  | MD | 0.263 | 0.195 | 0.273 |
|  | AxD | 0.096 | 0.64 | 0.64 |
|  | RD | 0.271 | 0.181 | 0.377 |
| **Cingulum cingulate gyrus R** | FA | -0.181 | 0.378 | 0.812 |
|  | MD | 0.134 | 0.513 | 0.544 |
|  | AxD | -0.107 | 0.602 | 0.639 |
|  | RD | 0.204 | 0.317 | 0.52 |
| **Cingulum hippocampus L** | FA | 0.190 | 0.352 | 0.812 |
|  | MD | 0.346 | 0.083 | 0.243 |
|  | AxD | 0.328 | 0.102 | 0.223 |
|  | RD | 0.325 | 0.105 | 0.332 |
| **Cingulum hippocampus R** | FA | 0.371 | 0.062 | 0.543 |
|  | MD | 0.276 | 0.172 | 0.273 |
|  | AxD | 0.414 | 0.035 | 0.106 |
|  | RD | 0.115 | 0.576 | 0.65 |
| **Corticospinal tract L** | FA | -0.223 | 0.273 | 0.812 |
|  | MD | 0.285 | 0.158 | 0.273 |
|  | AxD | 0.206 | 0.312 | 0.377 |
|  | RD | 0.393 | 0.047 | 0.233 |
| **Corticospinal tract R** | FA | -0.121 | 0.557 | 0.812 |
|  | MD | 0.281 | 0.164 | 0.273 |
|  | AxD | 0.255 | 0.209 | 0.292 |
|  | RD | 0.270 | 0.183 | 0.377 |
| **External capsule L** | FA | -0.152 | 0.458 | 0.812 |
|  | MD | 0.148 | 0.469 | 0.513 |
|  | AxD | 0.173 | 0.399 | 0.465 |
|  | RD | 0.137 | 0.504 | 0.609 |
| **External capsule R** | FA | -0.083 | 0.688 | 0.84 |
|  | MD | 0.087 | 0.672 | 0.672 |
|  | AxD | 0.101 | 0.624 | 0.64 |
|  | RD | 0.094 | 0.648 | 0.687 |
| **Fornix L** | FA | -0.008 | 0.97 | 0.988 |
|  | MD | 0.292 | 0.147 | 0.273 |
|  | AxD | 0.273 | 0.178 | 0.281 |
|  | RD | 0.294 | 0.145 | 0.339 |
| **Fornix R** | FA | 0.080 | 0.696 | 0.84 |
|  | MD | 0.325 | 0.105 | 0.243 |
|  | AxD | 0.248 | 0.222 | 0.297 |
|  | RD | 0.310 | 0.123 | 0.332 |
| **Genu of corpus callosum** | FA | -0.206 | 0.312 | 0.812 |
|  | MD | 0.430 | 0.028 | 0.161 |
|  | AxD | 0.438 | 0.025 | 0.099 |
|  | RD | 0.429 | 0.029 | 0.233 |
| **Posterior corona radiata L** | FA | 0.335 | 0.094 | 0.66 |
|  | MD | 0.247 | 0.224 | 0.29 |
|  | AxD | 0.355 | 0.075 | 0.175 |
|  | RD | 0.177 | 0.388 | 0.537 |
| **Posterior corona radiata R** | FA | 0.449 | 0.021 | 0.495 |
|  | MD | 0.337 | 0.092 | 0.243 |
|  | AxD | 0.406 | 0.04 | 0.107 |
|  | RD | 0.166 | 0.419 | 0.543 |
| **Posterior limb of internal capsule L** | FA | 0.127 | 0.537 | 0.812 |
|  | MD | 0.438 | 0.025 | 0.161 |
|  | AxD | 0.465 | 0.017 | 0.086 |
|  | RD | 0.186 | 0.362 | 0.537 |
| **Posterior limb of internal capsule R** | FA | 0.143 | 0.487 | 0.812 |
|  | MD | 0.240 | 0.238 | 0.297 |
|  | AxD | 0.369 | 0.064 | 0.159 |
|  | RD | 0.035 | 0.867 | 0.867 |
| **Posterior thalamic radiation L** | FA | 0.135 | 0.511 | 0.812 |
|  | MD | 0.256 | 0.206 | 0.278 |
|  | AxD | 0.244 | 0.229 | 0.297 |
|  | RD | 0.207 | 0.311 | 0.52 |
| **Posterior thalamic radiation R** | FA | 0.103 | 0.616 | 0.84 |
|  | MD | 0.286 | 0.156 | 0.273 |
|  | AxD | 0.284 | 0.159 | 0.281 |
|  | RD | 0.200 | 0.327 | 0.52 |
| **Retrolenticular part of internal capsule L** | FA | 0.072 | 0.726 | 0.847 |
|  | MD | 0.322 | 0.109 | 0.243 |
|  | AxD | 0.472 | 0.015 | 0.086 |
|  | RD | 0.119 | 0.564 | 0.65 |
| **Retrolenticular part of internal capsule R** | FA | 0.221 | 0.279 | 0.812 |
|  | MD | 0.386 | 0.051 | 0.21 |
|  | AxD | 0.462 | 0.018 | 0.086 |
|  | RD | 0.108 | 0.599 | 0.655 |
| **Sagittal stratum L** | FA | -0.197 | 0.335 | 0.812 |
|  | MD | 0.382 | 0.054 | 0.21 |
|  | AxD | 0.269 | 0.184 | 0.281 |
|  | RD | 0.395 | 0.046 | 0.233 |
| **Sagittal stratum R** | FA | -0.090 | 0.662 | 0.84 |
|  | MD | 0.296 | 0.143 | 0.273 |
|  | AxD | 0.219 | 0.283 | 0.354 |
|  | RD | 0.303 | 0.133 | 0.332 |
| **Splenium of corpus callosum** | FA | -0.173 | 0.397 | 0.812 |
|  | MD | 0.473 | 0.015 | 0.161 |
|  | AxD | 0.533 | 0.005 | 0.086 |
|  | RD | 0.374 | 0.06 | 0.233 |
| **Superior corona radiata L** | FA | 0.250 | 0.219 | 0.812 |
|  | MD | 0.477 | 0.014 | 0.161 |
|  | AxD | 0.529 | 0.005 | 0.086 |
|  | RD | 0.402 | 0.042 | 0.233 |
| **Superior corona radiata R** | FA | 0.411 | 0.037 | 0.495 |
|  | MD | 0.345 | 0.084 | 0.243 |
|  | AxD | 0.496 | 0.01 | 0.086 |
|  | RD | 0.178 | 0.383 | 0.537 |
| **Superior fronto occipital fasciculus L** | FA | 0.003 | 0.988 | 0.988 |
|  | MD | 0.320 | 0.111 | 0.243 |
|  | AxD | 0.321 | 0.11 | 0.227 |
|  | RD | 0.375 | 0.059 | 0.233 |
| **Superior fronto occipital fasciculus R** | FA | 0.316 | 0.116 | 0.674 |
|  | MD | 0.190 | 0.351 | 0.397 |
|  | AxD | 0.264 | 0.193 | 0.281 |
|  | RD | 0.051 | 0.804 | 0.827 |
| **Superior longitudinal fasciculus L** | FA | -0.401 | 0.042 | 0.495 |
|  | MD | 0.266 | 0.19 | 0.273 |
|  | AxD | 0.113 | 0.583 | 0.638 |
|  | RD | 0.316 | 0.116 | 0.332 |
| **Superior longitudinal fasciculus R** | FA | -0.049 | 0.813 | 0.918 |
|  | MD | 0.091 | 0.658 | 0.672 |
|  | AxD | 0.164 | 0.424 | 0.479 |
|  | RD | 0.152 | 0.458 | 0.573 |
| **Tapetum L** | FA | 0.126 | 0.538 | 0.812 |
|  | MD | 0.265 | 0.19 | 0.273 |
|  | AxD | 0.281 | 0.164 | 0.281 |
|  | RD | 0.229 | 0.26 | 0.483 |
| **Tapetum R** | FA | 0.134 | 0.514 | 0.812 |
|  | MD | 0.421 | 0.032 | 0.161 |
|  | AxD | 0.496 | 0.01 | 0.086 |
|  | RD | 0.395 | 0.046 | 0.233 |

Spearman's correlations were performed between the residuals of the linear model of regional DTI metrics and CDR global scores, controlling for age, sex, MRI-to-clinical visit interval and b-values.

**Supplementary Table 5 –** **WM region-of-interest group analyses corrected for Lewy Body co-pathology.**

|  |  | **CERAD**  **2 vs 3** | | **BRAAK**  **II-IV vs V/VI** | | **CAA** | | | | | | | | | | | | |
| --- | --- | --- | --- | --- | --- | --- | --- | --- | --- | --- | --- | --- | --- | --- | --- | --- | --- | --- |
|  |  |  |  |  |  |  | 0 vs 1 | | 0 vs 2 | | 0 vs 3 | | 1 vs 2 | | 1 vs 3 | | 2 vs 3 | |
| **Region** | Scalar | pval | adjp | pval | adjp | pval | pval | adjp | pval | adjp | pval | adjp | pval | adjp | pval | adjp | pval | adjp |
| **ACR L** | FA | 0.977 | 1 | 0.958 | 0.986 | 0.444 | - | - | - | - | - | - | - | - | - | - | - | - |
|  | MD | 0.796 | 1 | 0.025 | 0.22 | 0.159 | - | - | - | - | - | - | - | - | - | - | - | - |
|  | AxD | 0.437 | 0.853 | 0.005 | 0.185 | 0.197 | - | - | - | - | - | - | - | - | - | - | - | - |
|  | RD | 0.977 | 0.977 | 0.075 | 0.238 | 0.258 | - | - | - | - | - | - | - | - | - | - | - | - |
| **ACR R** | FA | 0.709 | 0.919 | 0.367 | 0.986 | 0.129 | - | - | - | - | - | - | - | - | - | - | - | - |
|  | MD | 1 | 1 | 0.164 | 0.274 | 0.39 | - | - | - | - | - | - | - | - | - | - | - | - |
|  | AxD | 0.796 | 0.969 | 0.164 | 0.359 | 0.473 | - | - | - | - | - | - | - | - | - | - | - | - |
|  | RD | 0.931 | 0.977 | 0.22 | 0.383 | 0.351 | - | - | - | - | - | - | - | - | - | - | - | - |
| **ALIC L** | FA | 0.472 | 0.889 | 0.751 | 0.986 | 0.949 | - | - | - | - | - | - | - | - | - | - | - | - |
|  | MD | 0.403 | 1 | 0.792 | 0.792 | 0.334 | - | - | - | - | - | - | - | - | - | - | - | - |
|  | AxD | 0.172 | 0.853 | 0.396 | 0.513 | 0.172 | - | - | - | - | - | - | - | - | - | - | - | - |
|  | RD | 0.546 | 0.977 | 0.958 | 0.958 | 0.512 | - | - | - | - | - | - | - | - | - | - | - | - |
| **ALIC R** | FA | 0.666 | 0.919 | 0.458 | 0.986 | 0.994 | - | - | - | - | - | - | - | - | - | - | - | - |
|  | MD | 0.546 | 1 | 0.711 | 0.732 | 0.64 | - | - | - | - | - | - | - | - | - | - | - | - |
|  | AxD | 0.472 | 0.853 | 0.426 | 0.514 | 0.505 | - | - | - | - | - | - | - | - | - | - | - | - |
|  | RD | 0.886 | 0.977 | 0.56 | 0.633 | 0.73 | - | - | - | - | - | - | - | - | - | - | - | - |
| **BCC** | FA | 0.312 | 0.889 | 0.958 | 0.986 | 0.496 | - | - | - | - | - | - | - | - | - | - | - | - |
|  | MD | 0.625 | 1 | 0.107 | 0.22 | 0.192 | - | - | - | - | - | - | - | - | - | - | - | - |
|  | AxD | 0.546 | 0.853 | 0.133 | 0.311 | 0.383 | - | - | - | - | - | - | - | - | - | - | - | - |
|  | RD | 0.752 | 0.977 | 0.181 | 0.374 | 0.131 | - | - | - | - | - | - | - | - | - | - | - | - |
| **CCG L** | FA | 0.977 | 1 | 0.133 | 0.986 | 0.183 | - | - | - | - | - | - | - | - | - | - | - | - |
|  | MD | 0.235 | 1 | 0.312 | 0.42 | 0.064 | - | - | - | - | - | - | - | - | - | - | - | - |
|  | AxD | 0.235 | 0.853 | 0.916 | 0.971 | 0.362 | - | - | - | - | - | - | - | - | - | - | - | - |
|  | RD | 0.666 | 0.977 | 0.085 | 0.238 | 0.047 | 0.839 | 1 | 0.761 | 1 | 0.877 | 0.877 | 0.016 | 0.047 | 0.012 | 0.075 | 0.063 | 0.127 |
| **CCG R** | FA | 0.138 | 0.889 | 0.792 | 0.986 | 0.669 | - | - | - | - | - | - | - | - | - | - | - | - |
|  | MD | 0.546 | 1 | 0.491 | 0.573 | 0.053 | - | - | - | - | - | - | - | - | - | - | - | - |
|  | AxD | 0.886 | 0.969 | 0.958 | 0.986 | 0.067 | - | - | - | - | - | - | - | - | - | - | - | - |
|  | RD | 0.403 | 0.977 | 0.426 | 0.533 | 0.199 | - | - | - | - | - | - | - | - | - | - | - | - |
| **CH L** | FA | 0.235 | 0.889 | 0.367 | 0.986 | 0.825 | - | - | - | - | - | - | - | - | - | - | - | - |
|  | MD | 0.841 | 1 | 0.12 | 0.22 | 0.19 | - | - | - | - | - | - | - | - | - | - | - | - |
|  | AxD | 0.886 | 0.969 | 0.241 | 0.383 | 0.261 | - | - | - | - | - | - | - | - | - | - | - | - |
|  | RD | 0.546 | 0.977 | 0.058 | 0.238 | 0.128 | - | - | - | - | - | - | - | - | - | - | - | - |
| **CHR** | FA | 0.371 | 0.889 | 0.458 | 0.986 | 0.358 | - | - | - | - | - | - | - | - | - | - | - | - |
|  | MD | 0.709 | 1 | 0.107 | 0.22 | 0.246 | - | - | - | - | - | - | - | - | - | - | - | - |
|  | AxD | 0.212 | 0.853 | 0.107 | 0.288 | 0.615 | - | - | - | - | - | - | - | - | - | - | - | - |
|  | RD | 0.709 | 0.977 | 0.241 | 0.383 | 0.107 | - | - | - | - | - | - | - | - | - | - | - | - |
| **CT L** | FA | 0.841 | 0.981 | 0.491 | 0.986 | 0.969 | - | - | - | - | - | - | - | - | - | - | - | - |
|  | MD | 0.977 | 1 | 0.095 | 0.22 | 0.762 | - | - | - | - | - | - | - | - | - | - | - | - |
|  | AxD | 0.752 | 0.969 | 0.367 | 0.513 | 0.905 | - | - | - | - | - | - | - | - | - | - | - | - |
|  | RD | 0.796 | 0.977 | 0.039 | 0.238 | 0.862 | - | - | - | - | - | - | - | - | - | - | - | - |
| **CT R** | FA | 0.285 | 0.889 | 0.148 | 0.986 | 0.967 | - | - | - | - | - | - | - | - | - | - | - | - |
|  | MD | 0.508 | 1 | 0.597 | 0.674 | 0.81 | - | - | - | - | - | - | - | - | - | - | - | - |
|  | AxD | 0.625 | 0.875 | 1 | 1 | 0.601 | - | - | - | - | - | - | - | - | - | - | - | - |
|  | RD | 0.796 | 0.977 | 0.367 | 0.494 | 0.803 | - | - | - | - | - | - | - | - | - | - | - | - |
| **EC L** | FA | 0.403 | 0.889 | 1 | 1 | 0.999 | - | - | - | - | - | - | - | - | - | - | - | - |
|  | MD | 0.585 | 1 | 0.095 | 0.22 | 0.957 | - | - | - | - | - | - | - | - | - | - | - | - |
|  | AxD | 0.285 | 0.853 | 0.066 | 0.257 | 0.933 | - | - | - | - | - | - | - | - | - | - | - | - |
|  | RD | 0.546 | 0.977 | 0.164 | 0.359 | 0.993 | - | - | - | - | - | - | - | - | - | - | - | - |
| **EC R** | FA | 0.371 | 0.889 | 0.491 | 0.986 | 0.744 | - | - | - | - | - | - | - | - | - | - | - | - |
|  | MD | 0.546 | 1 | 0.458 | 0.553 | 0.969 | - | - | - | - | - | - | - | - | - | - | - | - |
|  | AxD | 0.312 | 0.853 | 0.426 | 0.514 | 0.956 | - | - | - | - | - | - | - | - | - | - | - | - |
|  | RD | 0.796 | 0.977 | 0.525 | 0.613 | 0.835 | - | - | - | - | - | - | - | - | - | - | - | - |
| **Fx L** | FA | 0.709 | 0.919 | 0.181 | 0.986 | 0.178 | - | - | - | - | - | - | - | - | - | - | - | - |
|  | MD | 0.666 | 1 | 0.133 | 0.233 | 0.291 | - | - | - | - | - | - | - | - | - | - | - | - |
|  | AxD | 0.437 | 0.853 | 0.181 | 0.374 | 0.218 | - | - | - | - | - | - | - | - | - | - | - | - |
|  | RD | 0.585 | 0.977 | 0.085 | 0.238 | 0.312 | - | - | - | - | - | - | - | - | - | - | - | - |
| **Fx R** | FA | 0.709 | 0.919 | 0.025 | 0.874 | 0.064 | - | - | - | - | - | - | - | - | - | - | - | - |
|  | MD | 0.285 | 1 | 0.12 | 0.22 | 0.351 | - | - | - | - | - | - | - | - | - | - | - | - |
|  | AxD | 0.212 | 0.853 | 0.458 | 0.534 | 0.192 | - | - | - | - | - | - | - | - | - | - | - | - |
|  | RD | 0.403 | 0.977 | 0.039 | 0.238 | 0.211 | - | - | - | - | - | - | - | - | - | - | - | - |
| **GCC** | FA | 0.666 | 0.919 | 0.792 | 0.986 | 0.678 | - | - | - | - | - | - | - | - | - | - | - | - |
|  | MD | 0.666 | 1 | 0.263 | 0.369 | 0.307 | - | - | - | - | - | - | - | - | - | - | - | - |
|  | AxD | 0.585 | 0.853 | 0.22 | 0.383 | 0.109 | - | - | - | - | - | - | - | - | - | - | - | - |
|  | RD | 0.841 | 0.977 | 0.491 | 0.593 | 0.572 | - | - | - | - | - | - | - | - | - | - | - | - |
| **PCR L** | FA | 0.931 | 1 | 0.711 | 0.986 | 0.77 | - | - | - | - | - | - | - | - | - | - | - | - |
|  | MD | 0.886 | 1 | 0.045 | 0.22 | 0.242 | - | - | - | - | - | - | - | - | - | - | - | - |
|  | AxD | 0.886 | 0.969 | 0.034 | 0.235 | 0.266 | - | - | - | - | - | - | - | - | - | - | - | - |
|  | RD | 0.752 | 0.977 | 0.095 | 0.238 | 0.246 | - | - | - | - | - | - | - | - | - | - | - | - |
| **PCR R** | FA | 0.472 | 0.889 | 0.672 | 0.986 | 0.476 | - | - | - | - | - | - | - | - | - | - | - | - |
|  | MD | 0.931 | 1 | 0.2 | 0.304 | 0.18 | - | - | - | - | - | - | - | - | - | - | - | - |
|  | AxD | 0.977 | 1 | 0.2 | 0.383 | 0.12 | - | - | - | - | - | - | - | - | - | - | - | - |
|  | RD | 0.625 | 0.977 | 0.396 | 0.513 | 0.268 | - | - | - | - | - | - | - | - | - | - | - | - |
| **PLIC L** | FA | 0.371 | 0.889 | 0.916 | 0.986 | 0.571 | - | - | - | - | - | - | - | - | - | - | - | - |
|  | MD | 0.084 | 1 | 0.066 | 0.22 | 0.614 | - | - | - | - | - | - | - | - | - | - | - | - |
|  | AxD | 0.096 | 0.853 | 0.051 | 0.255 | 0.375 | - | - | - | - | - | - | - | - | - | - | - | - |
|  | RD | 0.371 | 0.977 | 0.287 | 0.402 | 0.77 | - | - | - | - | - | - | - | - | - | - | - | - |
| **PLIC R** | FA | 0.138 | 0.889 | 0.958 | 0.986 | 0.69 | - | - | - | - | - | - | - | - | - | - | - | - |
|  | MD | 0.508 | 1 | 0.241 | 0.351 | 0.401 | - | - | - | - | - | - | - | - | - | - | - | - |
|  | AxD | 0.108 | 0.853 | 0.12 | 0.299 | 0.312 | - | - | - | - | - | - | - | - | - | - | - | - |
|  | RD | 0.752 | 0.977 | 0.833 | 0.883 | 0.675 | - | - | - | - | - | - | - | - | - | - | - | - |
| **PTR L** | FA | 1 | 1 | 0.367 | 0.986 | 0.18 | - | - | - | - | - | - | - | - | - | - | - | - |
|  | MD | 1 | 1 | 0.2 | 0.304 | 0.119 | - | - | - | - | - | - | - | - | - | - | - | - |
|  | AxD | 1 | 1 | 0.312 | 0.475 | 0.272 | - | - | - | - | - | - | - | - | - | - | - | - |
|  | RD | 0.977 | 0.977 | 0.2 | 0.383 | 0.112 | - | - | - | - | - | - | - | - | - | - | - | - |
| **PTR R** | FA | 0.752 | 0.94 | 0.491 | 0.986 | 0.105 | - | - | - | - | - | - | - | - | - | - | - | - |
|  | MD | 0.931 | 1 | 0.396 | 0.495 | 0.193 | - | - | - | - | - | - | - | - | - | - | - | - |
|  | AxD | 0.841 | 0.969 | 0.367 | 0.513 | 0.209 | - | - | - | - | - | - | - | - | - | - | - | - |
|  | RD | 0.977 | 0.977 | 0.287 | 0.402 | 0.156 | - | - | - | - | - | - | - | - | - | - | - | - |
| **RPIC L** | FA | 0.212 | 0.889 | 0.597 | 0.986 | 0.71 | - | - | - | - | - | - | - | - | - | - | - | - |
|  | MD | 1 | 1 | 0.12 | 0.22 | 0.17 | - | - | - | - | - | - | - | - | - | - | - | - |
|  | AxD | 0.437 | 0.853 | 0.095 | 0.288 | 0.399 | - | - | - | - | - | - | - | - | - | - | - | - |
|  | RD | 0.341 | 0.977 | 0.263 | 0.401 | 0.268 | - | - | - | - | - | - | - | - | - | - | - | - |
| **RPIC R** | FA | 0.508 | 0.889 | 0.339 | 0.986 | 0.62 | - | - | - | - | - | - | - | - | - | - | - | - |
|  | MD | 0.546 | 1 | 0.107 | 0.22 | 0.415 | - | - | - | - | - | - | - | - | - | - | - | - |
|  | AxD | 0.371 | 0.853 | 0.396 | 0.513 | 0.527 | - | - | - | - | - | - | - | - | - | - | - | - |
|  | RD | 0.886 | 0.977 | 0.075 | 0.238 | 0.385 | - | - | - | - | - | - | - | - | - | - | - | - |
| **SS L** | FA | 0.709 | 0.919 | 0.148 | 0.986 | 0.159 | - | - | - | - | - | - | - | - | - | - | - | - |
|  | MD | 0.796 | 1 | 0.039 | 0.22 | 0.358 | - | - | - | - | - | - | - | - | - | - | - | - |
|  | AxD | 0.546 | 0.853 | 0.058 | 0.255 | 0.625 | - | - | - | - | - | - | - | - | - | - | - | - |
|  | RD | 0.931 | 0.977 | 0.029 | 0.238 | 0.2 | - | - | - | - | - | - | - | - | - | - | - | - |
| **SS R** | FA | 0.709 | 0.919 | 0.22 | 0.986 | 0.149 | - | - | - | - | - | - | - | - | - | - | - | - |
|  | MD | 0.931 | 1 | 0.107 | 0.22 | 0.025 | 0.241 | 0.361 | 0.736 | 0.736 | 0.164 | 0.328 | 0.097 | 0.29 | 0.002 | 0.015 | 0.251 | 0.301 |
|  | AxD | 0.931 | 0.988 | 0.241 | 0.383 | 0.102 | - | - | - | - | - | - | - | - | - | - | - | - |
|  | RD | 0.977 | 0.977 | 0.085 | 0.238 | 0.015 | 0.218 | 0.327 | 0.532 | 0.532 | 0.079 | 0.237 | 0.089 | 0.179 | 0.002 | 0.01 | 0.371 | 0.445 |
| **SCC** | FA | 0.259 | 0.889 | 0.56 | 0.986 | 0.596 | - | - | - | - | - | - | - | - | - | - | - | - |
|  | MD | 0.931 | 1 | 0.075 | 0.22 | 0.021 | 0.993 | 0.993 | 0.071 | 0.106 | 0.048 | 0.096 | 0.024 | 0.071 | 0.011 | 0.066 | 0.826 | 0.992 |
|  | AxD | 0.341 | 0.853 | 0.029 | 0.235 | 0.131 | - | - | - | - | - | - | - | - | - | - | - | - |
|  | RD | 0.709 | 0.977 | 0.148 | 0.346 | 0.041 | 0.774 | 0.774 | 0.147 | 0.22 | 0.067 | 0.133 | 0.05 | 0.149 | 0.013 | 0.079 | 0.761 | 0.914 |
| **SCR L** | FA | 0.977 | 1 | 0.792 | 0.986 | 0.734 | - | - | - | - | - | - | - | - | - | - | - | - |
|  | MD | 0.796 | 1 | 0.021 | 0.22 | 0.315 | - | - | - | - | - | - | - | - | - | - | - | - |
|  | AxD | 0.585 | 0.853 | 0.029 | 0.235 | 0.396 | - | - | - | - | - | - | - | - | - | - | - | - |
|  | RD | 0.977 | 0.977 | 0.051 | 0.238 | 0.283 | - | - | - | - | - | - | - | - | - | - | - | - |
| **SCR R** | FA | 0.508 | 0.889 | 0.833 | 0.986 | 0.934 | - | - | - | - | - | - | - | - | - | - | - | - |
|  | MD | 0.796 | 1 | 0.107 | 0.22 | 0.165 | - | - | - | - | - | - | - | - | - | - | - | - |
|  | AxD | 0.472 | 0.853 | 0.107 | 0.288 | 0.241 | - | - | - | - | - | - | - | - | - | - | - | - |
|  | RD | 0.886 | 0.977 | 0.22 | 0.383 | 0.208 | - | - | - | - | - | - | - | - | - | - | - | - |
| **SFOF L** | FA | 0.841 | 0.981 | 0.458 | 0.986 | 0.183 | - | - | - | - | - | - | - | - | - | - | - | - |
|  | MD | 0.064 | 1 | 0.085 | 0.22 | 0.484 | - | - | - | - | - | - | - | - | - | - | - | - |
|  | AxD | 0.064 | 0.853 | 0.095 | 0.288 | 0.292 | - | - | - | - | - | - | - | - | - | - | - | - |
|  | RD | 0.108 | 0.977 | 0.095 | 0.238 | 0.533 | - | - | - | - | - | - | - | - | - | - | - | - |
| **SFOF R** | FA | 0.508 | 0.889 | 0.874 | 0.986 | 0.542 | - | - | - | - | - | - | - | - | - | - | - | - |
|  | MD | 0.403 | 1 | 0.672 | 0.732 | 0.273 | - | - | - | - | - | - | - | - | - | - | - | - |
|  | AxD | 0.259 | 0.853 | 0.672 | 0.735 | 0.191 | - | - | - | - | - | - | - | - | - | - | - | - |
|  | RD | 0.752 | 0.977 | 0.672 | 0.735 | 0.281 | - | - | - | - | - | - | - | - | - | - | - | - |
| **SLF L** | FA | 0.259 | 0.889 | 0.263 | 0.986 | 0.163 | - | - | - | - | - | - | - | - | - | - | - | - |
|  | MD | 0.437 | 1 | 0.396 | 0.495 | 0.269 | - | - | - | - | - | - | - | - | - | - | - | - |
|  | AxD | 0.709 | 0.954 | 0.241 | 0.383 | 0.474 | - | - | - | - | - | - | - | - | - | - | - | - |
|  | RD | 0.403 | 0.977 | 0.241 | 0.383 | 0.273 | - | - | - | - | - | - | - | - | - | - | - | - |
| **SLF R** | FA | 0.259 | 0.889 | 0.916 | 0.986 | 0.558 | - | - | - | - | - | - | - | - | - | - | - | - |
|  | MD | 0.371 | 1 | 0.711 | 0.732 | 0.091 | - | - | - | - | - | - | - | - | - | - | - | - |
|  | AxD | 0.437 | 0.853 | 0.597 | 0.674 | 0.037 | 0.248 | 0.371 | 0.295 | 0.354 | 0.895 | 0.895 | 0.174 | 0.348 | 0.007 | 0.045 | 0.024 | 0.071 |
|  | RD | 0.341 | 0.977 | 0.874 | 0.9 | 0.134 | - | - | - | - | - | - | - | - | - | - | - | - |
| **Tp L** | FA | 0.508 | 0.889 | 0.672 | 0.986 | 0.602 | - | - | - | - | - | - | - | - | - | - | - | - |
|  | MD | 0.625 | 1 | 0.051 | 0.22 | 0.427 | - | - | - | - | - | - | - | - | - | - | - | - |
|  | AxD | 0.546 | 0.853 | 0.045 | 0.255 | 0.438 | - | - | - | - | - | - | - | - | - | - | - | - |
|  | RD | 0.585 | 0.977 | 0.045 | 0.238 | 0.37 | - | - | - | - | - | - | - | - | - | - | - | - |
| **Tp R** | FA | 0.403 | 0.889 | 0.525 | 0.986 | 0.818 | - | - | - | - | - | - | - | - | - | - | - | - |
|  | MD | 0.192 | 1 | 0.018 | 0.22 | 0.073 | - | - | - | - | - | - | - | - | - | - | - | - |
|  | AxD | 0.341 | 0.853 | 0.029 | 0.235 | 0.078 | - | - | - | - | - | - | - | - | - | - | - | - |
|  | RD | 0.172 | 0.977 | 0.016 | 0.238 | 0.065 | - | - | - | - | - | - | - | - | - | - | - | - |

Statistical analyses of regional DTI metrics linear model residuals for age, sex, b-values, MRI-to-death interval and presence of Lewy Body were performed using the Wilcoxon test for the CERAD scores 2 and 3 and for Braak pathology stages II-IV and V/VI, while the Kruskal-Wallis test followed by Dunn's post-hoc test for pairwise comparisons was applied to the CAA groups. To account for multiple comparisons, Benjamini-Hochberg false discovery rate corrections were applied. The JHU-ICBM-DTI-48 WM atlas was used to extract regional DTI metrics for each subject. ROIs abbreviations: ACR - Anterior Corona Radiata, ALIC - Anterior limb of internal capsule, BCC - Body of corpus callosum, CCG - Cingulum cingulate gyrus, CH - Cingulum hippocampus, CT - Corticospinal tract, EC – External capsule, Fx - Fornix, GCC - Genu of corpus callosum, PCR - Posterior corona radiata, PLIC - Posterior limb of internal capsule, PTR - Posterior thalamic radiation, RPIC - Retrolenticular part of internal capsule, SS - Sagittal stratum, SCC - Splenium of corpus callosum, SCR - Superior corona radiata, SFOF - Superior fronto occipital fasciculus, SLF - Superior longitudinal fasciculus, Tp - Tapetum.
